# Psychological and behavioral mechanisms linking childhood trauma profiles to voice-related distress in schizophrenia spectrum disorders: latent class, mediation, and conditional process analyses

**DOI:** 10.1017/S0033291726104437

**Published:** 2026-06-08

**Authors:** Mads Juul Christensen, Anne Vingaard Olesen, Thomas Ward, Amy Hardy, Jan Mainz, Neil Thomas, Lisa Charlotte Smith, Lise Sandvig Mariegaard, Merete Nordentoft, Louise Birkedal Glenthøj, Ditte Lammers Vernal

**Affiliations:** 1 Department of Psychiatry, North Denmark Region, Aalborg University Hospital, Aalborg, Denmark; 2 Department of Clinical Medicine, The Faculty of Medicine, Aalborg University, Aalborg, Denmark; 3 Research Data and Biostatistics, North Denmark Region,Aalborg University Hospital, Aalborg, UK; 4 Department of Psychology, Institute of Psychiatry, Psychology & Neuroscience, King’s College London, London, United Kingdom; 5 South London & Maudsley NHS Foundation Trust, London, United Kingdom; 6 Department of Health Economics,University of Southern Denmark, Odense, Denmark; 7 Centre for Mental Health and Brain Sciences,Swinburne University of Technology, Melbourne, Australia; 8 Alfred Hospital, Melbourne, Australia; 9 VIRTU Research Group, Mental Health Center Copenhagen, ,Copenhagen University Hospital – Mental Health Services CPH, Denmark; 10 Department of Clinical Medicine,University of Copenhagen, Denmark; 11 Research Unit (CORE), Capital Region of Denmark,Mental Health Center Copenhagen, Denmark; 12 Department of Psychology, University of Copenhagen, Denmark

**Keywords:** voice-hearing, distressing voices, auditory verbal hallucinations, childhood trauma, trauma profiles, trauma-psychosis links, latent class analysis, mediation analysis, conditional process analysis, schizophrenia spectrum disorders, persecutory beliefs about voices, sleep disturbances

## Abstract

**Background:**

Childhood traumas increase the risk of psychosis and voice-hearing. While trauma profiles have been identified in voice-hearers, pathways linking these to voice-related distress remain unclear. This study examined between-group differences in mediation by psychological and behavioral variables in profile–distress associations, with potential moderation by gender.

**Methods:**

This cross-sectional study derived childhood trauma profiles via latent class analysis of Childhood Trauma Questionnaire (CTQ) scores from 266 voice-hearing Challenge trial participants with schizophrenia-spectrum diagnoses. Mediation analyses (structural equation modeling with bootstrapped 95% confidence intervals for indirect effects) tested between-group differences in indirect effects of negative voice content, persecutory beliefs about voices, voice power, voice relating style, negative self/other beliefs, emotion regulation, depression, and sleep disturbances in the association between childhood trauma profiles (exposure) and voice-related distress (outcome), with gender as a moderator. Hypotheses were preregistered on the Open Science Framework. Reporting followed AGReMA-guidelines.

**Results:**

Three childhood trauma profiles were established: (1) ‘variable severity’ (*n* = 160), (2) ‘severe neglect and emotional abuse’ (*n* = 84), and (3) ‘severe poly-trauma’ (*n* = 22). Significant between-group differences in indirect effects were observed for persecutory beliefs about voices (1 < 3), voice power (1 < 3), and sleep disturbances (1 < 2). Age-adjustment revealed a between-group difference in indirect effect of negative self-beliefs (1 < 3). No moderation by gender was found.

**Conclusions:**

This is the first investigation of mediators and moderators of childhood trauma profiles and voice-related distress in clinical voice-hearers. Findings suggest that trauma profiles may provide indicators of mediators potentially relevant to inform individualized formulation and therapy planning.

## Introduction

Childhood trauma is associated with increased risk of psychosis, with an estimated overall odds ratio of 2.80 (Zhou et al., 2025). Multiple and recurrent childhood trauma exert cumulative effects on the risk with a dose–response relationship (Flinn et al., 2025) arguably most researched for hallucinations and delusions (Bailey et al., 2018; Muenzenmaier et al., 2015). Evidence further suggests that distinct childhood trauma types are associated with different symptom dimensions (Alameda et al., 2021) and specific psychotic symptoms (Grindey & Bradshaw, 2022). Despite the extensive literature and estimates suggesting that childhood trauma is a primary contributing factor in 38% of global cases of schizophrenia spectrum disorders (SSD) (Dragioti et al., 2022), childhood trauma often remains undetected in mental health services (Read, Harper, Tucker, & Kennedy, 2018). Having a more granular understanding of how different trauma experiences impact on psychosis may facilitate it being incorporated into treatment.

Psychosis is more common in people with a history of trauma (Vila-Badia et al., 2021). People exposed to trauma may subsequently experience hallucinations, such as hearing voices (Daalman et al., 2012; McCarthy-Jones, 2011; Misiak, Moustafa, Kiejna, & Frydecka, 2016). Voice content and individuals’ interpretations of voices often appear meaningfully related to life experiences, including trauma (Peach et al., 2021; Van Den Berg et al., 2023). While voice-hearing is not inherently pathological (Baumeister, Sedgwick, Howes, & Peters, 2017; Corstens et al., 2014; Longden, 2017), it can involve significant shame (Volpato et al., 2022) and distress (Badcock, Graham, & Paulik, 2020; Larøi et al., 2012, 2019; Tsang et al., 2021), making voice-related distress a key focus of clinical assessment and intervention (Loizou et al., 2022; Toh et al., 2022). Notably, females report greater voice distress, overall voice severity, and more resistant responses to voices than males (Hayward, Slater, Berry, & Perona-Garcelán, 2016; Schlier et al., 2021; Toh et al., 2020), despite evidence of no gender differences in overall positive symptom severity (Carter et al., 2022).

Trauma research is complicated by substantial methodological heterogeneity, including variation in trauma conceptualization and operationalization (Denton et al., 2017; Sætren et al., 2024). Traumatic events are often categorized by type, with their co-occurrence rarely considered despite forming predictable clusters (Jacobs, Agho, Stevens, & Raphael, 2012; O’Donnell et al., 2017). Data-driven approaches (e.g. latent class or latent profile analysis) instead derive trauma typologies from observed patterns within a sample (Roesch, Villodas, & Villodas, 2010), potentially offering greater clinical relevance (Saunders, Buckman, & Pilling, 2020). In psychosis research, such analyses have identified profiles including ‘childhood abuse’ and ‘childhood neglect’ (Stevens et al., 2019); ‘low adversity’, ‘lack of support and isolation’, and ‘abuse and neglect’ (Carbone et al., 2019); and ‘emotional abuse and neglect’, ‘physical abuse’, ‘sexual abuse’, and ‘poly-victimization’ (Barnes, Emsley, Garety, & Hardy, 2021). A study spanning the psychosis spectrum (clinical voice-hearers, non-clinical voice-hearers, and non-voice-hearers) identified ‘low trauma’, ‘emotion-focused trauma’, and ‘multi-trauma’ profiles, with the latter predominantly comprising clinical voice-hearers and reporting more adverse voice characteristics (Begemann et al., 2022). Another study identified four profiles among clinical voice-hearers: ‘adverse voice and relational trauma’, ‘low malevolent and omnipotent voices’, and ‘adverse voices yet low relational trauma’, with the relational-trauma profile showing the most severe voices and greatest emotional distress (Marotti, Saunders, Montague, & Fornells-Ambrojo, 2025). Collectively, voice-hearing occurs across trauma profiles; voice severity varies by trauma profile; and the intersection of trauma types (i.e. complex trauma) has a more potent impact on psychosis than discrete trauma types.

Multiple pathways may connect childhood trauma and psychosis (Bloomfield et al., 2021; Isvoranu et al., 2017; Sideli et al., 2020; Williams, Bucci, Berry, & Varese, 2018), including PTSD-related mechanisms (Hardy, 2017; Hardy et al., 2016, 2021). Identifying mediators of the trauma–psychosis relationship may inform potential treatment targets (Alameda et al., 2020). However, existing research tends to focus on frequency or overall severity of psychotic experiences, with less attention given to clinical outcomes targeted in therapy and valued by people with lived experience, such as voice-related distress (Lincoln et al., 2024; Steel et al., 2007; Thomas, 2015). Studies linking childhood trauma to voice-related distress suggest mediation via anxious attachment (Pilton et al., 2016) or negative voice content (Rosen et al., 2018), though it should be noted that sample sizes were small and trauma co-occurrence was not considered.

Guided by existing theories and empirical findings, we hypothesized that negative voice content, persecutory beliefs about voices, voice power, voice relating style, negative self/other beliefs, emotion regulation, depression, and sleep disturbances may differentially mediate the association between childhood trauma profiles and voice-related distress, with gender moderating the effects. These variables have been associated with trauma and/or voice-related distress (see Table 1 and Supplementary Material A for details). Investigating between-group differences in mediation of these variables may provide clinically useful indicators for personalized formulation and intervention planning. Accordingly, this study aimed to (1) derive childhood trauma profiles in a large sample of clinical voice-hearers with SSD and (2) test between-group differences in mediation and moderation in the association between childhood trauma and voice-related distress, estimating presence and magnitude of direct, indirect, and conditional effects. The mediation analyses were explanatory, not interventional.

**Table 1.** Overview of exposure, outcome, mediator, moderator, and confounder variables, including action and conceptual theories for candidate mediators Table 1. long description.

Analytic role	Variable	Measure	
Exposure	Childhood trauma	The Childhood Trauma Questionnaire–Short Form (CTQ-SF; Bernstein, Fink, Handelsman, & Foote, 1998; Bernstein et al., 2003) is a 28-item, 5-point (1–5) self-report questionnaire assessing childhood abuse (sexual, emotional, physical) and neglect (emotional, physical), yielding five subscale scores (5–25) and a total score (25–125). The CTQ-SF is considered gold-standard (Airey, Taylor, Vikram, & Berry, 2023). We used the Danish CTQ-SF (Bernstein and Fink, 2011; Kongerslev et al., 2019).	
Outcome	Voice distress	Psychotic Symptom Rating Scale–Auditory Hallucinations Subscale (PSYRATS-AHS; Haddock, McCarron, Tarrier, & Faragher, 1999) is an 11-item, 5-point (0–4) clinician-administered measure of voice severity with good psychometric properties and extensive usage (Drake et al., 2007; Kronmüller et al., 2011; Woodward et al., 2014). We used a distress-items sum score (items 8 + 9; 0–9; Rosen et al., 2018; Varese et al., 2016).	

Analytic role	Variable	Conceptual and action theory	Measure
Mediator	Negative voice content	Negative voice content is associated with childhood trauma (Begemann et al., 2022) and considered a driver of voice-distress (Larøi et al., 2019; Silver, Lewton, & Lewis, 2023).	PSYRATS-AHS (Haddock et al., 1999). We used a negative content-item sum score (items 6 + 7; 0–8; Rosen et al., 2018).
	Persecutory beliefs about voices	Trauma has predicted variance in voice appraisals (Andrew et al., 2008) and appraisals have predicted voice-related distress (Cole, Strauss, Fife-Schaw, & McCarthy-Jones, 2017; Peters, Williams, Cooke, & Kuipers, 2012) in prior studies. Appraisals such as malevolence, power, and dominance are associated with distress (Birchwood et al., 2004; Chadwick and Birchwood, 1994; Tsang et al., 2021).	Beliefs about Voices Questionnaire–Revised (BAVQ-R; Chadwick, Lees, & Max, 2000) is a 35-item, 4-point (0–3) self-report questionnaire with satisfactory psychometric properties and wide usage (Ratcliff, Farhall, & Shawyer, 2011). We used ‘Persecutory Beliefs Factor’ (0–27) (Strauss et al., 2018).
	Voice power	*Same as above.*	Voice Power Differential Scale (VPS; Birchwood et al., 2000) is a 7-item, 5-point (1–5) self-report questionnaire with good psychometric properties (Gmeiner et al., 2020). Higher scores indicate greater perceived voice power (7–35).
	Relating to voices	Relating to voices can be shaped by life history and trauma (Corstens and Longden, 2013). More passive and less assertive relating are associated with voice-distress in females (Hayward et al., 2016; Schlier et al., 2021).	Approve-Voice (Approve; Hayward et al., 2020) is a 15-item, 11-point (0–10) self-report questionnaire on voice relating (assertive, aggressive, passive/submissive), with three subscales (0–50).
	Negative self/other beliefs	Negative self/other beliefs are associated with childhood trauma and insecure attachment (Hardy et al., 2016; Scott et al., 2020). It has mediated trauma and psychosis (Jorovat, Twumasi, Mechelli, & Georgiades, 2025) and predicted voice-distress via negative voice beliefs (Cole et al., 2017) in prior studies.	Brief Core Schema Scale (BCSS; Fowler et al., 2006) is a 24-item, 5-point (0–4) self-report questionnaire with good psychometric properties (Faustino, 2023). We used the negative subscales (6 items each; 0–24).
	Emotion regulation	Emotion regulation difficulties are associated with childhood trauma (Gruhn and Compas, 2020; Lavi, Katz, Ozer, & Gross, 2019; Miu et al., 2022). Maladaptive regulation like meta-worrying (Morrison and Wells, 2007) and rumination (Badcock, Paulik, & Maybery, 2011) are linked to voice-distress.	Emotion Regulation Questionnaire (ERQ; Gross and John, 2003) is a 10-item, 7-point (1–5) self-report questionnaire on habitual use of cognitive reappraisal and expressive suppression, yielding two subscale scores (5–35). The two-factor structure is validated (Melka et al., 2011).
	Depression	Depression is associated with trauma (Dragioti et al., 2022); could be predicted by trauma in females with SSD (Köhler-Forsberg et al., 2024); has mediated negative schemas and voice-distress (Kusztrits et al., 2022), but neither childhood adversities and voice-distress (Rosen et al., 2018) nor positive symptoms (Barnes et al., 2023) in prior studies.	Calgary Depression Scale for Schizophrenia (CDSS; Addington, Addington, & Maticka-tyndale, 1993) is a 9-item, 4-point (0–3) clinician-administered interview assessing severity of depressive symptoms over the past 2 weeks (total 0–27). It has good psychometric properties (Addington, Addington, & Atkinson, 1996; Porter, Jones, & Fox, 2022).
	Sleep distrubances	Sleep disturbances are associated with childhood adversities (Schønning et al., 2022) and psychosis (Bagautdinova et al., 2023; Reeve, Sheaves, & Freeman, 2015) and could mediate childhood adversities and psychopathology (Laskemoen et al., 2021; Liu et al., 2023), including interpersonal trauma and paranoia-related distress (Herms, Bolbecker, & Wisner, 2024) in prior studies.	Pittsburgh Sleep Quality Index (PSQI; Buysse et al., 1989) is a 24-item questionnaire assessing sleep quality over the past month. PSQI is widely used, though its dimensionality remains debated (Manzar et al., 2018). We used the 19 self-report items, yielding seven component scores summed into a global score (0–21).
Moderator	Gender	Gender differences in psychosis exist (Carter et al., 2022). Females report greater voice severity, distress, and more resistant relating styles than males (Hayward et al., 2016; Murphy, Shevlin, Adamson, & Houston, 2010; Schlier et al., 2021; Toh et al., 2020).	Self-report
Confounder	Age		Extracted from case report

*Note*: For details, see Supplementary Material A.

## Methods

### Study registration

The protocol and statistical analysis plan were prospectively registered on the Open Science Framework (OSF) (https://osf.io/s6dtf), with no deviations. Reporting followed A Guideline for Reporting Mediation Analyses (AGReMA) (Lee et al., 2021).

### Study design and source of data

This observational study applied a cross-sectional design using baseline data collected prior to randomization in Challenge, a randomized multi-center clinical trial on virtual reality (VR)-assisted therapy for distressing voices in SSD. Elsewhere, more details are provided on Challenge’s methodology (Smith et al., 2022), outcomes (Smith et al., 2025), user and clinical experiences (Christensen et al., 2025; Rasmussen et al., 2026; Vernal et al., 2023), and secondary analyses of spatial presence (Glenthøj et al., 2026) and emotion regulation (Glenthøj et al., n.d.).

### Participants

The target population was individuals diagnosed with SSD experiencing treatment-resistant, distressing voices. Inclusion criteria: adults (≥18); SSD diagnosis (ICD-10); persistent auditory hallucinations (≥3 months, SAPS ≥3); currently in treatment at a Danish psychiatric facility; able to provide informed consent; no changes in antipsychotic mediation within the past ≥4 weeks; and insufficient response to current antipsychotic treatment; or, if not currently treated, to ≥2 previous antipsychotic compounds. Exclusion criteria: inability to identify a dominant voice; organic brain disease; substance use interfering with attendance; hearing voices in an unsupported language; inadequate Danish/English proficiency; inability to tolerate assessment procedures; or severe visual impairment. Challenge was conducted across three Danish sites. Enrollment started on 16 November 2020; follow-up ended on 27 February 2024.

### Sample size

The sample comprised all 270 participants enrolled in Challenge. Because the trial was powered for its primary hypotheses rather than the secondary analyses reported here, post hoc power calculations for this fixed sample would be uninformative.

### Effects of interest

Effects of interest included the direct, indirect, and total effect of the exposure (childhood trauma profile) on the outcome (voice-related distress).

### Assumed causal model

**Figure 1.**
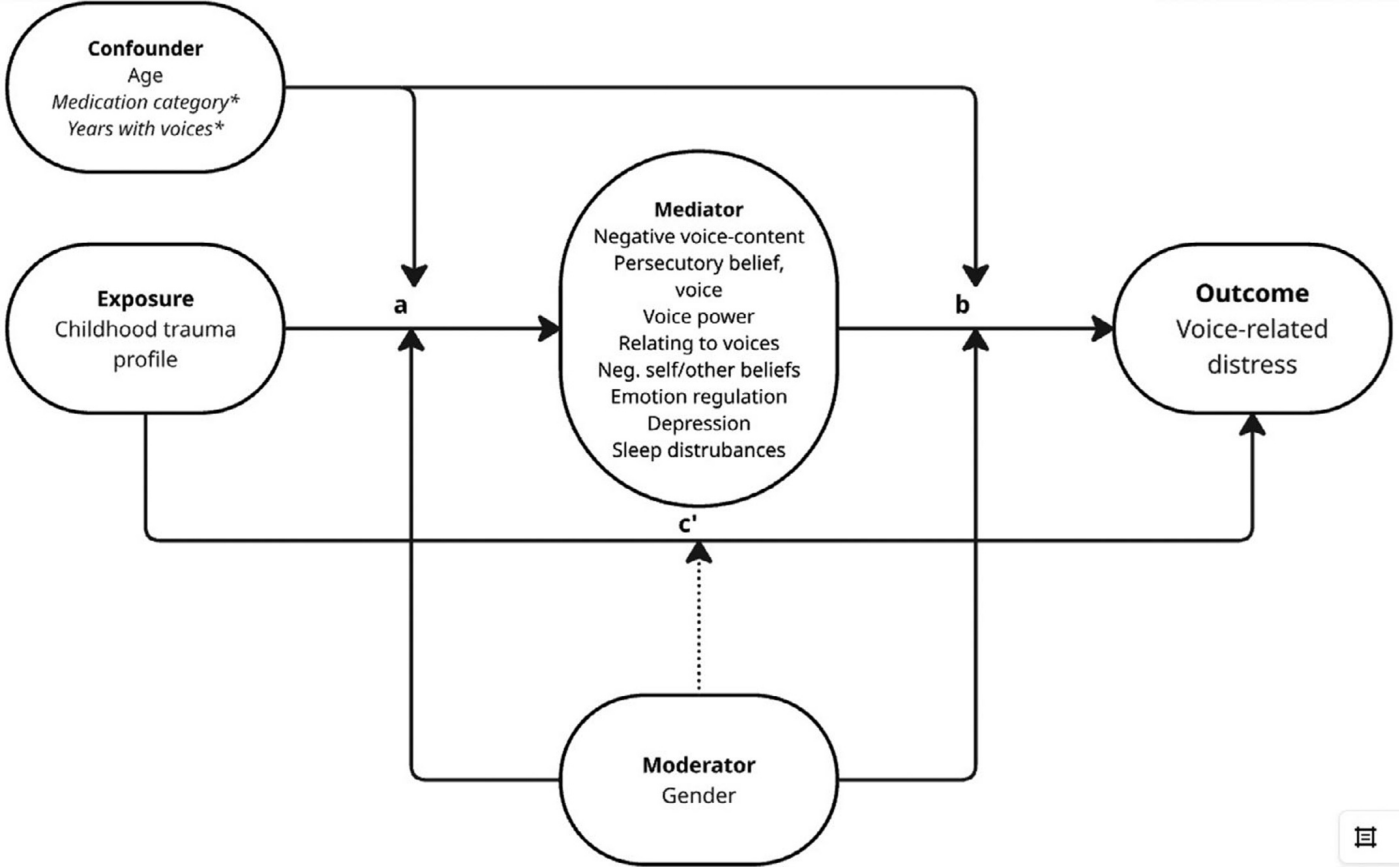
Causal directed acyclic graph illustrating assumed causal model and interplay among variables. Figure 1. long description.

Figure 1 illustrates the assumed causal model in a causal directed acyclic graph (Lipsky & Greenland, 2022), including exposure, mediator, outcome, moderator, and potential confounders.

### Model assumptions

The tested model assumed that exposure to childhood trauma impacts voice-related distress via one or more of the candidate mediators, with gender moderating the effect, and age potentially confounding. The model assumed that more severe childhood trauma would exert a negative effect on the variable of interest, which in turn would negatively influence voice-related distress. In the exposure–mediator relationship, we assumed that childhood trauma predates the onset of voices as well as the other psychopathological variables. In the mediator–outcome relationship, we assumed that candidate mediators predate voice-related distress. However, temporal precedence cannot be definitively established, and reverse causality cannot be precluded. People with psychosis are more likely to experience both past and current traumatic events, and early difficulties could plausibly increase the risk of mistreatment from caregivers. Likewise, bidirectional relationships may exist between the proposed mediators and distress (e.g. distress impacting sleep). Nonetheless, the present study specifically tested the hypothesized pathway in which childhood trauma influences the mediator, which in turn influences voice-related distress.

### Measurement

Table 1 displays measures and variables, including conceptual (linking exposure to mediators) and action theories (linking mediators to outcome).

### Measurement levels

The exposure, mediator, and outcome variables were measured at the individual level. Following LCA, the exposure was assigned according to identified groupings also on the individual level.

### Statistical methods

All statistical analyses were conducted in Stata v18 (StataCorp, 2023). Participant characteristics were summarized using means and standard deviations or frequencies and percentages. Mediation and conditional process analyses followed current conceptual and statistical guidelines (Hayes and Rockwood, 2020; Igartua and Hayes, 2021). Accordingly, we focused on the difference between total and direct effects of the exposure and evaluated whether zero could be plausibly excluded from the confidence interval of the indirect effect. The statistical significance or pattern of the total or direct effect did not determine the presence or absence of mediation (Igartua and Hayes, 2021).

#### Latent class analysis (LCA)

LCA (a maximum likelihood-based alternative to conventional partitional cluster analysis and thus with a basis on likelihood-based statistical theory) was used to identify participant subgroups based on CTQ scores. Model selection was guided by the Akaike Information Criterion (AIC; Akaike, 1974) and Bayesian Information Criterion (BIC; Schwarz, 1978), with lower values indicating better statistical fit. AIC and BIC are goodness-of-fit measures used to evaluate how well class solutions minimize distance between respondents within a class and maximize the distance between classes. Selection of the optimal number of classes also considered the interpretability of the resulting profiles and their consistency with prior theory and empirical research (Roesch, Villodas, & Villodas, 2010).

LCA classes were turned into childhood trauma profiles by interpreting average CTQ scores and distribution of severity across subscales (Table 2). Danish, gender-specific CTQ reference norms were used to determine severity (Bernstein and Fink, 2011).

**Table 2.** Participant demographics and clinical characteristics Table 2. long description.

Profile	1 ‘Variable trauma’	2 ‘Severe neglect and emotional abuse’	3 ‘Severe poly-trauma’	Total	Missings/*N* (%)
*n* (%)	160 (60.2)	84 (31.6)	22 (8.3)	266 (100.0)	0/266 (0.0)
Gender, *n* (%)					
Female	91 (56.9)	58 (69.0)	14 (63.6)	163 (61.3)	
Male	69 (43.1)	26 (31.0)	8 (36.4)	103 (38.7)	0/266 (0.0)
Age, mean (sd)	32.7 (12.1)	32.7 (11.6)	34.0 (11.3)	32.8 (11.8)	0/266 (0.0)
Education, *n* (%)					
Primary	61 (38.1)	36 (42.9)	7 (31.8)	104 (39.1)	
Secondary	42 (26.2)	27 (32.1)	9 (40.9)	78 (29.3)	
Vocational	34 (21.2)	14 (16.7)	3 (13.6)	51 (19.2)	
University	16 (10.0)	6 (7.1)	1 (4.5)	23 (8.6)	
Other	7 (4.4)	1 (1.2)	2 (9.1)	10 (3.8)	0/266 (0.0)
Occupation, *n* (%)					
1 Full-time	3 (1.9)	2 (2.4)	1 (4.5)	6 (2.3)	
2 Part-time	14 (8.8)	6 (7.1)	0 (0.0)	20 (7.5)	
3 Student	20 (12.5)	8 (9.5)	4 (18.2)	32 (12.0)	
4 Stay-at-home	0	0	0	0	
5 Unemployed	53 (33.1)	39 (46.4)	5 (22.7)	97 (36.5)	
6 Retired, early retirement pension, or post-employment benefit	57 (35.6)	23 (27.4)	11 (50.0)	91 (34.2)	
7 Other	13 (8.1)	6 (7.1)	1 (4.5)	20 (7.5)	0/266 (0.0)
Years with voice-hearing, mean (sd)	14.7 (11.5)	17.0 (10.4)	19.0 (9.6)	15.8 (11.1)	8/266 (3.0)
Attending a voice-hearing group outside psychiatric services, *n* (%)					
No	155 (96.9)	80 (96.4)	21 (95.5)	256 (96.6)	
Yes	5 (3.1)	3 (3.6)	1 (4.5)	9 (3.4)	1/266 (0.4)
Any antipsychotics (AP) medication use, *n* (%)	148 (92.5)	73 (86.9)	19 (86.4)	240 (90.2)	0/266 (0.0)
Dosage of olanzapine equivalent of AP (mg/day), (sd)	20.69 (14.59)	16.07 (12.22)	18.43 (11.90)	19.04 (13.78)	6/266 (2.3)
First-generation AP use, *n* (%)	20 (12.5)	8 (9.5)	2 (9.1)	30 (11.3)	0/266 (0.0)
Clozapine use, *n* (%)	45 (28.1)	12 (14.3)	4 (18.2)	61 (22.9)	0/266 (0.0)
Non-sedative AP use, *n* (%)	82 (51.2)	43 (51.2)	13 (59.1)	138 (51.9)	0/266 (0.0)
Sedative AP use, *n* (%)	55 (34.4)	26 (31.0)	9 (40.9)	90 (33.8)	0/266 (0.0)
Diagnosis (ICD–10)					
Schizophrenia (F20)	149 (93.1)	77 (91.7)	19 (86.4)	245 (92.1)	
Persistent delusional disorders (F22.8/F22.9)	3 (1.9)	1 (1.2)	0 (0.0)	4 (1.5)	
Schizoaffective disorders (F25)	3 (1.9)	2 (2.4)	1 (4.5)	6 (2.3)	
Other non-organic psychotic disorders (F28 and F29)	5 (3.1)	4 (4.8)	2 (9.1)	11 (4.1)	0/266 (0.0)
Childhood trauma (total CTQ), mean (sd)	36.62 (7.92)	63.93 (10.00)	92.05 (15.25)	49.83 (20.09)	0/266 (0.0)
Emotional abuse, mean (sd)	8.75 (3.21)	18.40 (3.62)	21.95 (3.53)	12.89 (6.17)	0/266 (0.0)
None, *n* (%)	57 (35.6)	0 (0.0)	0 (0.0)	57 (21.4)	
Low, *n* (%)	36 (22.5)	0 (0.0)	0 (0.0)	36 (13.5)	
Moderate, *n* (%)	27 (16.9)	0 (0.0)	0 (0.0)	27 (10.2)	
Severe, *n* (%)	40 (25.0)	84 (100.0)	22 (100.0)	146 (54.9)	
Physical abuse, mean (sd)	5.46 (1.27)	7.35 (2.35)	19.59 (3.84)	7.22 (4.29)	0/266 (0.0)
None, *n* (%)	130 (81.2)	30 (35.7)	0 (0.0)	160 (60.2)	
Moderate, *n* (%)	13 (8.1)	10 (11.9)	0 (0.0)	23 (8.6)	
Severe, *n* (%)	17 (10.6)	44 (52.4)	22 (100.0)	83 (31.2)	
Sexual abuse, mean (sd)	6.42 (3.78)	9.45 (5.94)	15.27 (8.24)	8.11 (5.62)	0/266 (0.0)
None, *n* (%)	129 (80.6)	39 (46.4)	4 (18.2)	172 (64.7)	
Moderate, *n* (%)	5 (3.1)	4 (4.8)	1 (4.5)	10 (3.8)	
Severe, *n* (%)	26 (16.2)	41 (48.8)	17 (77.3)	84 (31.6)	
Emotional neglect, mean (sd)	9.36 (3.62)	17.54 (4.14)	19.32 (4.82)	12.76 (5.73)	0/266 (0.0)
None, *n* (%)	83 (51.9)	6 (7.1)	0 (0.0)	89 (33.5)	
Low, *n* (%)	41 (25.6)	0 (0.0)	3 (13.6)	44 (16.5)	
Moderate, *n* (%)	15 (9.4)	8 (9.5)	0 (0.0)	23 (8.6)	
Severe, *n* (%)	21 (13.1)	70 (83.3)	19 (86.4)	110 (41.4)	
Physical neglect, mean (sd)	6.64 (2.11)	11.19 (3.86)	15.91 (5.19)	8.84 (4.28)	0/266 (0.0)
None, *n* (%)	72 (45.0)	3 (3.6)	0 (0.0)	75 (28.2)	
Low, *n* (%)	26 (16.2)	4 (4.8)	1 (4.5)	31 (11.7)	
Moderate, *n* (%)	19 (11.9)	5 (6.0)	0 (0.0)	24 (9.0)	
Severe, *n* (%)	43 (26.9)	72 (85.7)	21 (95.5)	136 (51.1)	
Voice-related distress (items 8 + 9 PSYRATS-AHS), mean (sd)	6.34 (1.30)	6.30 (1.29)	6.59 (1.33)	6.35 (1.30)	0/266 (0.0)
Negative voice-content (items 6 + 7 PSYRATS-AHS), mean (sd)	6.88 (1.36)	6.86 (1.35)	7.14 (1.04)	6.89 (1.33)	0/266 (0.0)
Persecutory belief factor (subscale BAVQ-R), mean (sd)	17.20 (6.14)	17.63 (6.13)	20.00 (5.64)	17.56 (6.12)	1/266 (0.4)
Voice power differential (total VPDS), mean (sd)	24.75 (5.34)	25.52 (4.37)	26.82 (4.36)	25.16 (5.00)	3/266 (1.1)
Assertive relating (subscale Approve), mean (sd)	22.08 (13.29)	20.86 (14.61)	25.86 (15.34)	22.01 (13.90)	1/266 (0.4)
Aggressive responding (subscale Approve), mean (sd)	14.75 (12.22)	18.02 (14.63)	26.00 (17.65)	16.68 (13.81)	4/266 (1.5)
Passive/submissive responding (subscale Approve), mean (sd)	22.93 (14.28)	23.59 (12.86)	28.93 (15.12)	23.62 (13.95)	2/266 (0.8)
Negative other-beliefs (subscale BCSS), mean (sd)	6.65 (5.77)	7.40 (5.77)	7.36 (6.77)	6.95 (5.85)	5/266 (1.9)
Negative self-beliefs (subscale BCSS), mean (sd)	10.12 (5.75)	11.52 (6.26)	12.36 (5.46)	10.76 (5.93)	5/266 (1.9)
Cognitive reappraisal (subscale ERQ), mean (sd)	23.39 (7.61)	22.49 (8.94)	23.27 (8.76)	23.10 (8.13)	1/266 (0.4)
Expressive suppression (subscale ERQ), mean (sd)	16.34 (5.58)	16.38 (5.44)	15.73 (6.08)	16.30 (5.56)	1/266 (0.4)
Depression (total CDSS), mean (sd)	6.08 (4.38)	6.66 (4.76)	8.23 (5.25)	6.44 (4.60)	6/266 (2.3)
Sleep disturbances (total PSQI), mean (sd)	9.59 (4.29)	11.36 (3.95)	11.38 (4.25)	10.30 (4.25)	5/266 (1.9)

*Note*: Participant demographics and clinical characteristics, including the number and percentage of participants within each childhood trauma profile who met trauma-severity thresholds for each CTQ trauma domain (range: none to severe), based on Danish CTQ reference norms from Bernstein and Fink (2011). Cut-offs were emotional abuse: none (5–6 males, 5–7 females), low (7 males, 8–9 females), moderate (8–9 males, 10–11 females), severe (≥10 males, ≥12 females); physical abuse: none (5), moderate (6), severe (≥7); sexual abuse: none (5), moderate (6), severe (≥7); emotional neglect: none (5–8 males, 5–9 females), low (9–12 males, 10–11 females), moderate (13–14 males, 12–13 females), severe (≥15 males, ≥14 females); physical neglect: none (5), low (6), moderate (7), severe (≥8). Abbreviations: sd, standard deviations; ICD-10, International Statistical Classification of Diseases and Related Health Problems 10th Revision. Medication classes, first-generation antipsychotics (Chlorprothixene, Haloperidol, Perphenazine, Zuclopenthixol); Clozapine; non-sedating antipsychotics (Aripiprazole, Amisulpride, Lurasidone); sedating antipsychotics (Olanzapine, Quetiapine). Measures, Approve, Approve-Voices; BAVQ-R, Beliefs About Voices Questionnaire–Revised; CTQ, Childhood Trauma Questionnaire; BCSS, Brief Core Schema Scale; CDSS, Calgary Depression Scale for Schizophrenia; ERQ, Emotion Regulation Questionnaire; PSQI; Pittsburgh Sleep Quality Index; VPDS, Voice Power Differential Scale.

#### Mediation analysis

Traditional mediation analyses (Hayes and Rockwood, 2020) were conducted with the two implicit regression models estimated simultaneously using structural equation modeling (SEM). While the exposure variable is categorical (childhood trauma profile), the outcome (voice-related distress) and candidate mediators (negative voice content, persecutory beliefs about voices, voice power, voice relating, negative self/other beliefs, emotion regulation, depression, and sleep disturbances) were continuous variables. All regression models were linear. Indirect effects were evaluated using 95% percentile-based confidence intervals generated from 5,000 bootstrap resamples.

#### Conditional process analysis/moderated mediation

Moderation of indirect effects by gender (conditional process analysis) was examined by introducing interaction terms by gender in the regression models, as recommended elsewhere (Hayes and Rockwood, 2020). Moderation was evaluated using bootstrapped confidence intervals for the difference in indirect effects between males and females.

### Sensitivity parameters

In line with the preregistered protocol, sensitivity analyses were adjusted for age as a three-category variable based on tertiles and examined alternative operationalizations of both the exposure and outcome, as well as the impact of excluding participants with CTQ minimization/denial scores >0 (see Supplementary Material B). Following reviewer suggestions, post hoc sensitivity analyses incorporated antipsychotic class (first-generation, clozapine, non-sedating second-generation, sedating second-generation) and duration of voice hearing (tertiles) as potential confounders.

### Ethical approval

The Challenge trial protocol was approved by the Committee on Health Research Ethics of the Capital Region of Denmark (H-19086621) and the Danish Data Protection Agency (P-2020-506), registered at ClinicalTrials.gov (NCT04661163), and published (Smith et al., 2022). All participants received written information and provided informed consent, as detailed elsewhere (Smith et al., 2025).

## Results

### Participants

After excluding participants with missing CTQ data (*n* = 4), the analytic sample comprised 266 participants (see Table 2 for characteristics). Missing data were minimal, with 98–100% data completeness (Table 2).

### Childhood trauma classes

**Figure 2.**
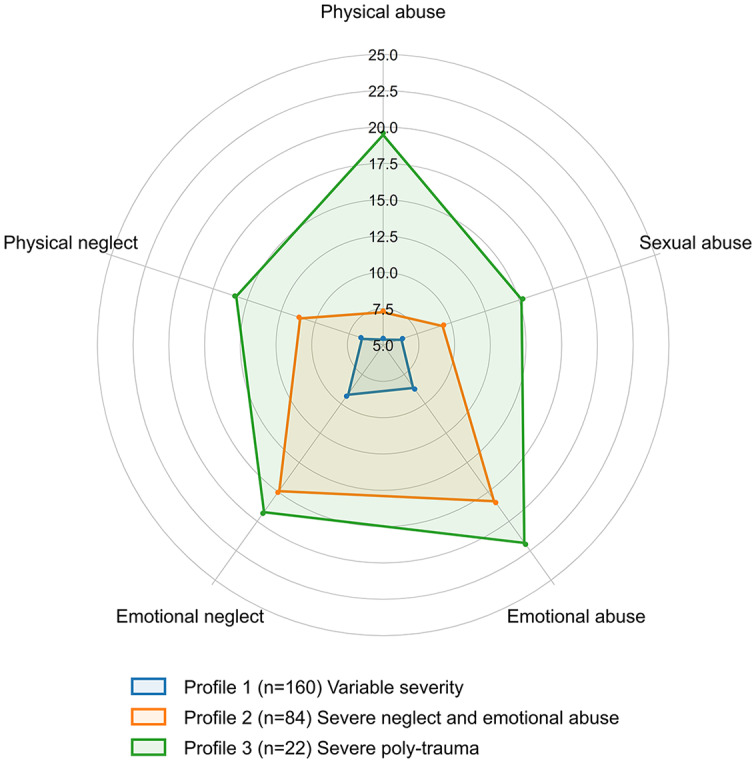
Childhood Trauma Questionnaire (CTQ) subscale means scores per profile. *Note:* This figure does not reflect trauma severity classifications but only mean CTQ subscale scores. See Table 2 for severity classifications. Figure 2. long description.

**Figure 3.**
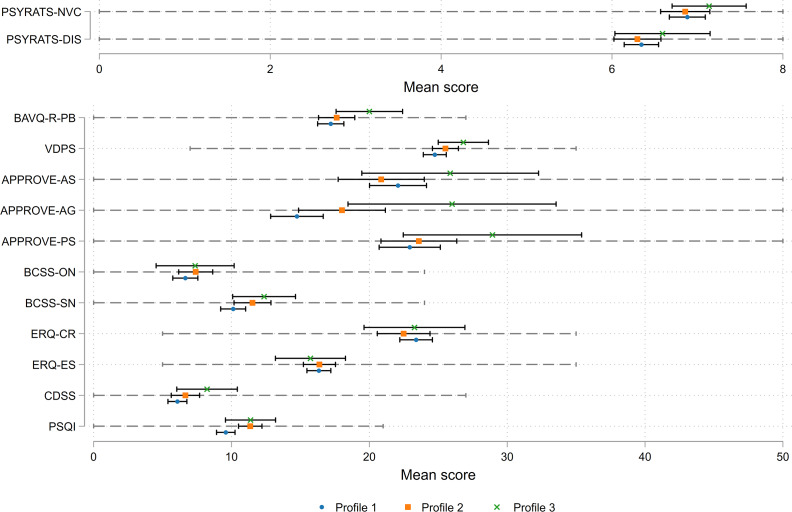
Forest plot comparing childhood trauma profiles on means and 95% confidence intervals of mediator and outcome variables. *Note*: Forest plot comparing childhood trauma profiles on means and confidence intervals of mediator and outcome variables. Dotted lines designate the range of each scale. PSYRATS variables separated out as the comparable lower scale range would impair interpretation. Measures, Approve-AS, Approve-Voices, Assertive Relating; Approve-AG, Approve-Voices, Aggressive Relating; Approve-PS, Approve-Voices, Passive/submissive Relating; BAVQ-R-PB, Beliefs About Voices Questionnaire–Revised, Persecutory Beliefs About Voices; CTQ, Childhood Trauma Questionnaire; BCSS-ON, Brief Core Schema Scale, Negative Other Beliefs; BCSS-SN, Brief Core Schema Scale, Negative Self Beliefs; CDSS, Calgary Depression Scale for Schizophrenia; ERQ-CR, Emotion Regulation Questionnaire Cognitive Reappraisal; ERQ-ES, Emotion Regulation Questionnaire Expressive Suppression; PSYRATS-NVC, Psychotic Symptom Rating Scale, Negative Voice Content; PSYRATS-DIS, Psychotic Symptom Rating Scale, Voice-related Distress; PSQI; Pittsburgh Sleep Quality Index; VPDS, Voice Power Differential Scale. Figure 3. long description.

Following LCA, a 3-class model was chosen as the most appropriate (AIC/BIC values in Supplementary Material C Table 11). A 4-class model largely subdivided existing classes without adding meaningful differentiation. Higher-order models (7 or 9 class) offered slightly lower AIC/BIC values, but improvements in model fit were minimal compared to moving from a 1- or 2-class solution to a 3-class solution. Higher-order models would also introduce greater complexity by yielding smaller, less stable subgroups. The 3-class model therefore balanced statistical fit, interpretability, and theoretical relevance for subsequent analyses. Table 2 lists profile characteristics; Figure 2 illustrates trauma type severity; and Figure 3 shows mediator and outcome means.

Profiles were characterized by (1) ‘variable severity’ (*n* = 160; 60,2%), (2) ‘severe neglect and emotional abuse’ (*n* = 84; 31,6%), and (3) ‘severe poly-trauma’ (*n* = 22; 8,3%). Profile 1 showed moderate severity of emotional and sexual abuse but low scores on physical abuse and physical and emotional neglect based on average scores. However, severity varied widely across individuals, ranging none-severe on all CTQ subscales. Class 2 showed high average severity across all CTQ subscales, with a clear dispersion toward severe levels for emotional abuse and both physical and emotional neglect. Sexual and physical abuse, however, showed a more polarized distribution, ranging none-severe. Class 3 showed extremely severe average scores on all CTQ subscales, with an almost uniformly severe distribution across all subscales.

### Outcomes and estimates

All outcomes for the mediation analyses are presented in Table 3, including point estimates and uncertainty estimates. No statistically significant between-group differences were detected in either the direct or total effects, indicating that the association between childhood trauma and voice-related distress did not differ across profiles, regardless of whether the indirect effect was included in the estimation of the association.

**Table 3.** Point estimates and uncertainty estimates for total, direct, and indirect effects Table 3. long description.

	Class	*N*	Indirect	95% CI Lower	95% CI Upper	*P*	Direct	95% CI Lower	95% CI Upper	*P*	Total	95% CI Lower	95% CI Upper	*P*
H1 Negative voice-content (items 6 + 7 PSYRATS-AHS)	2 vs 1	266	−0.015	−0.258	0.201	0.912	−0.031	−0.297	0.232	0.827	−0.046	−0.400	0.285	0.790
	3 vs 1	266	0.159	−0.173	0.447	0.300	0.089	−0.356	0.526	0.657	0.247	−0.408	0.800	0.391
	3 vs 2	266	0.174	−0.166	0.489	0.287	0.120	−0.341	0.572	0.593	0.293	−0.391	0.872	0.349
H2 Persecutory belief factor (subscale BAVQ-R)	2 vs 1	265	0.041	−0.108	0.202	0.615	−0.087	−0.417	0.217	0.593	−0.046	−0.399	0.287	0.800
	3 vs 1	265	0.265	0.003	0.538	**0.048**	−0.085	−0.649	0.417	0.792	0.180	−0.466	0.751	0.528
	3 vs 2	265	0.224	−0.039	0.507	0.093	0.002	−0.594	0.539	0.960	0.226	−0.449	0.826	0.452
H3 Voice power differential (total VPDS)	2 vs 1	263	0.065	−0.043	0.179	0.233	−0.082	−0.417	0.234	0.616	−0.016	−0.359	0.310	0.922
	3 vs 1	263	0.175	0.007	0.377	**0.041**	0.074	−0.493	0.557	0.720	0.249	−0.364	0.796	0.372
	3 vs 2	263	0.110	−0.055	0.308	0.194	0.156	−0.445	0.675	0.547	0.266	−0.365	0.851	0.379
H4 Assertive relating (subscale Approve)	2 vs 1	265	0.021	−0.044	0.097	0.517	−0.107	−0.434	0.221	0.528	−0.086	−0.420	0.240	0.620
	3 vs 1	265	−0.065	−0.212	0.056	0.270	0.272	−0.407	0.848	0.362	0.207	−0.428	0.749	0.448
	3 vs 2	265	−0.086	−0.246	0.040	0.172	0.379	−0.302	0.995	0.254	0.293	−0.347	0.863	0.331
H5 Aggressive responding (subscale Approve)	2 vs 1	262	−0.026	−0.090	0.012	0.211	−0.051	−0.404	0.297	0.778	−0.077	−0.437	0.263	0.664
	3 vs 1	262	−0.090	−0.275	0.033	0.162	0.402	−0.301	1.008	0.229	0.312	−0.327	0.856	0.299
	3 vs 2	262	−0.064	−0.226	0.029	0.212	0.453	−0.240	1.086	0.195	0.390	−0.265	0.980	0.226
H6 Passive/submissive responding (subscale Approve)	2 vs 1	264	0.017	−0.079	0.110	0.720	−0.078	−0.421	0.235	0.636	−0.061	−0.424	0.262	0.719
	3 vs 1	264	0.158	−0.019	0.377	0.082	0.150	−0.459	0.655	0.566	0.308	−0.335	0.853	0.302
	3 vs 2	264	0.140	−0.038	0.361	0.129	0.229	−0.391	0.766	0.415	0.369	−0.277	0.939	0.242
H7 Negative other-beliefs (subscale BCSS)	2 vs 1	261	0.009	−0.015	0.048	0.554	−0.053	−0.396	0.288	0.811	−0.044	−0.386	0.290	0.844
	3 vs 1	261	0.008	−0.034	0.072	0.750	0.241	−0.384	0.790	0.422	0.249	−0.382	0.796	0.406
	3 vs 2	261	−0.000	−0.051	0.055	0.994	0.294	−0.372	0.877	0.362	0.293	−0.380	0.876	0.361
H8 Negative self-beliefs (subscale BCSS)	2 vs 1	261	0.062	−0.006	0.167	0.085	−0.087	−0.440	0.244	0.639	−0.025	−0.380	0.306	0.910
	3 vs 1	261	0.099	−0.004	0.257	0.066	0.169	−0.463	0.701	0.550	0.268	−0.363	0.821	0.372
	3 vs 2	261	0.037	−0.084	0.179	0.536	0.256	−0.392	0.806	0.404	0.293	−0.376	0.870	0.364
H9 Cognitive reappraisal (subscale ERQ)	2 vs 1	265	0.017	−0.026	0.080	0.461	−0.059	−0.405	0.278	0.747	−0.042	−0.390	0.292	0.820
	3 vs 1	265	0.002	−0.080	0.098	0.928	0.249	−0.374	0.782	0.398	0.251	−0.380	0.804	0.403
	3 vs 2	265	−0.015	−0.114	0.076	0.740	0.308	−0.351	0.870	0.319	0.293	−0.360	0.866	0.343
H10 Expressive suppression (subscale ERQ)	2 vs 1	265	0.001	−0.039	0.051	0.927	−0.043	−0.388	0.283	0.813	−0.042	−0.390	0.292	0.820
	3 vs 1	265	−0.013	−0.100	0.063	0.740	0.265	−0.379	0.823	0.380	0.251	−0.380	0.804	0.403
	3 vs 2	265	−0.014	−0.107	0.060	0.709	0.308	−0.351	0.888	0.328	0.293	−0.360	0.866	0.342
H11 Depression (total CDSS)	2 vs 1	260	0.047	−0.053	0.157	0.354	−0.100	−0.453	0.229	0.586	−0.053	−0.421	0.283	0.794
	3 vs 1	260	0.172	−0.012	0.396	0.067	0.073	−0.524	0.617	0.780	0.245	−0.386	0.791	0.412
	3 vs 2	260	0.126	−0.066	0.357	0.202	0.173	−0.438	0.739	0.560	0.298	−0.382	0.887	0.358
H12 Sleep disturbances (total PSQI)	2 vs 1	261	0.112	0.029	0.225	**0.002**	−0.124	−0.462	0.219	0.489	−0.012	−0.344	0.339	0.953
	3 vs 1	261	0.113	−0.007	0.273	0.064	0.073	−0.507	0.602	0.751	0.186	−0.446	0.749	0.505
	3 vs 2	261	0.001	−0.137	0.128	0.980	0.197	−0.389	0.743	0.469	0.199	−0.442	0.790	0.507

*Note*: Point estimates and uncertainty estimates for total, direct, and indirect effects. Abbreviations: H#, Hypothesis no.; Class 1, low/moderate trauma cluster; Class 2, multi-trauma cluster; Class 3, extreme multi-trauma cluster. Indirect, indirect effect; Direct, direct effect; Total, total effect; Lower/upper, lower and upper uncertainty estimates Measures, Approve, Approve-Voices; BAVQ-R, Beliefs About Voices Questionnaire–Revised; CTQ, Childhood Trauma Questionnaire; BCSS, Brief Core Schema Scale; CDSS, Calgary Depression Scale for Schizophrenia; ERQ, Emotion Regulation Questionnaire; PSQI, Pittsburgh Sleep Quality Index; VPDS, Voice Power Differential Scale. Significance level: *p* < .05 (highlighted). All CI set at 95%.

Significant between-group differences in indirect effects were detected for persecutory beliefs about voices (profile 1 < 3), voice power (profile 1 < 3), and sleep disturbances (profile 1 < 2). Between-group differences remained significant after age-adjustment (Supplementary Material C Tables 1–2), which additionally revealed a significant (profile 1 < 3) and near-significant (profile 1 < 2) difference in indirect effect for negative self-beliefs. These results indicate between-group differences in how childhood trauma profiles influence voice-related distress through specific mediators. Gender did not moderate indirect effects (Table 4), indicating that mediation pathways were similar for males and females.

**Table 4.** Differences in indirect effect according to gender (female vs male) Table 4. long description.

	Classes	Estimate	95% CI Lower	95% CI Upper	*P*
H1 Negative voice-content (items 6 + 7 PSYRATS-AHS)	2 vs 1	−0.442	−0.964	0.010	0.057
	3 vs 1	−0.359	−1.078	0.199	0.232
	3 vs 2	0.083	−0.642	0.744	0.796
H2 Persecutory belief factor (subscale BAVQ-R)	2 vs 1	0.167	−0.144	0.508	0.308
	3 vs 1	−0.068	−0.644	0.481	0.796
	3 vs 2	−0.234	−0.857	0.348	0.414
H3 Voice power differential (total VPDS)	2 vs 1	−0.151	−0.386	0.046	0.144
	3 vs 1	0.113	−0.212	0.443	0.470
	3 vs 2	0.264	−0.057	0.619	0.100
H4 Assertive relating (subscale Approve)	2 vs 1	0.059	−0.071	0.202	0.374
	3 vs 1	−0.088	−0.408	0.148	0.450
	3 vs 2	−0.146	−0.478	0.091	0.228
H5 Aggressive responding (subscale Approve)	2 vs 1	0.050	−0.057	0.264	0.446
	3 vs 1	0.153	−0.196	0.605	0.384
	3 vs 2	0.103	−0.185	0.472	0.511
H6 Passive/submissive responding (subscale Approve)	2 vs 1	0.051	−0.128	0.242	0.574
	3 vs 1	0.102	−0.339	0.512	0.562
	3 vs 2	0.051	−0.413	0.457	0.732
H7 Negative other-beliefs (subscale BCSS)	2 vs 1	0.020	−0.051	0.113	0.549
	3 vs 1	0.061	−0.132	0.268	0.547
	3 vs 2	0.040	−0.152	0.232	0.669
H8 Negative self-beliefs (subscale BCSS)	2 vs 1	0.014	−0.166	0.189	0.798
	3 vs 1	0.005	−0.283	0.271	0.899
	3 vs 2	−0.009	−0.299	0.249	0.977
H9 Cognitive reappraisal (subscale ERQ)	2 vs 1	0.017	−0.093	0.134	0.706
	3 vs 1	0.095	−0.106	0.366	0.356
	3 vs 2	0.078	−0.152	0.372	0.502
H10 Expressive suppression (subscale ERQ)	2 vs 1	0.042	−0.083	0.188	0.522
	3 vs 1	−0.042	−0.266	0.159	0.634
	3 vs 2	−0.084	−0.341	0.144	0.429
H11 Depression (total CDSS)	2 vs 1	−0.039	−0.268	0.163	0.697
	3 vs 1	0.136	−0.245	0.527	0.458
	3 vs 2	0.175	−0.215	0.579	0.344
H12 Sleep disturbances (total PSQI)	2 vs 1	0.025	−0.215	0.231	0.744
	3 vs 1	0.137	−0.144	0.418	0.292
	3 vs 2	0.112	−0.159	0.417	0.409

*Note*: Point estimates and uncertainty estimates for differences in indirect effect according to gender (female vs male). Abbreviations: H#, Hypothesis no.; Class 1, ‘variable trauma profile’; Class 2, ‘severe neglect and emotional abuse profile’; Class 3, ‘severe poly-trauma profile’. Estimate, Estimate of difference in indirect effects comparing female and male participants; lower/upper, lower and upper uncertainty estimates Measures, Approve, Approve-Voices; BAVQ-R, Beliefs About Voices Questionnaire–Revised; CTQ, Childhood Trauma Questionnaire; BCSS, Brief Core Schema Scale; CDSS, Calgary Depression Scale for Schizophrenia; ERQ, Emotion Regulation Questionnaire; PSQI, Pittsburgh Sleep Quality Index; VPDS, Voice Power Differential Scale. Significance level, *p* < .05 (highlighted). All CI set at 95%.

### Sensitivity parameters

All sensitivity parameter outcomes are reported in Supplementary Material C Tables 1–15. Sensitivity analyses generally supported the main findings, with the most robust between-group difference in indirect effect observed for sleep disturbances (profile 1 < 2).

## Discussion

This study demonstrated between-group differences in mediators between childhood trauma profiles and voice-related distress in clinical voice-hearers. This approach is relevant given the frequent co-occurrence of childhood trauma in this population (e.g., Begemann et al., 2022) and may offer advantages over assessing individual trauma types, treating trauma as binary construct, or using a summative score, as trauma profiles better capture distinct patterns within trauma histories (O’Donnell et al., 2017). A clearer understanding of between-group differences of mediators linking trauma profiles to voice-related distress may inform individualized formulation and therapy planning. Three childhood trauma profiles were established: (1) ‘variable severity’, (2) ‘severe neglect and emotional abuse’, and (3) ‘severe poly-trauma’. Significant between-group differences in indirect effect were detected for persecutory beliefs about voices (profiles 1 < 3), voice power (profiles 1 < 3), and sleep disturbances (profiles 1 < 2). Gender did not moderate effects.

### Interpretation

#### Childhood trauma profiles

Childhood trauma was not uniformly reported but showed discrete patterns, consistent with prior work (Begemann et al., 2022; Marotti et al., 2025). While the largest profile reported low trauma on average, supporting the notion of multiple pathways to voice-hearing beyond trauma (Luhrmann et al., 2019), there was considerable variability within this profile, with a substantial proportion also reporting severe trauma. Despite this variability, the three profiles’ configuration suggested a trauma severity gradient, with profile 1 reflecting lower exposure, profile 2 higher exposure, and profile 3 the most extreme trauma. This pattern partly mirrors that identified by Begemann et al. (2022) and is consistent with findings from other profile-based trauma studies (O’Donnell et al., 2017). However, differences in sample characteristics and methods (e.g. reference norms used to interpret profiles) limit direct comparison. Specifically, CTQ-cutoffs may not generalize across populations (Thombs, Bernstein, Lobbestael, & Arntz, 2009). As LCA-derived profiles are sensitive to study design and sample size (Roesch, Villodas, & Villodas, 2010) and may not represent true population subgroups (Williams and Kibowski, 2016), replication in other contexts is needed.

#### Mechanisms linking childhood trauma profiles and voice-related distress

Most hypothesized mediators showed no between-group differences in indirect effects, which was surprising, particularly given the pronounced trauma gradient between profiles 1 and 3. This suggests that many mechanisms may be relevant and operate independently of trauma history. The largest statistically significant difference (persecutory beliefs about voices) corresponded to only a 0.27-point change in distress, which remained small in sensitivity analyses (maximum 0.67 on the 0–8 scale). While between-group differences were fewer and smaller than anticipated, they indicate that, for a subgroup of voice-hearers, childhood trauma influences voice-related distress indirectly through persecutory beliefs about voices, perceived voice power, and sleep disturbances. This suggests that these mechanisms are rooted in early trauma for some individuals. The findings complement previous work proposing differential pathways from trauma to specific psychotic symptoms (Alameda et al., 2020; Bloomfield et al., 2021; Williams et al., 2018) and linking specific trauma types to symptom dimensions (Alameda et al., 2021). However, pathways connecting childhood trauma and voice-related distress are likely more complex and individualized than examined here, as suggested by research showing that attachment style influences distress via interpersonal schemas and persecutory beliefs about voices (Cole et al., 2017).

The between-group difference of **negative self-beliefs** (profile 1 < 3) suggests that individuals in the most severe trauma profile experience more voice-related distress due to negative beliefs about themselves. This aligns with evidence that trauma-related beliefs are associated with hallucinations and delusions (Frost, Collier, & Hardy, 2024) and that negative self/other beliefs can mediate the relationship between childhood trauma and positive symptoms (Alameda et al., 2020; Bloomfield et al., 2021; Jorovat et al., 2025; Williams et al., 2018). The results also align with previous research linking voice-related distress to negative self-beliefs (Smith et al., 2006) and combined negative self/other beliefs (Cole et al., 2017). Notably, these associations appear more consistent for voices than for delusions (Barnes et al., 2023; Hardy et al., 2016). Similarly, the results on **persecutory beliefs about voices** and **voice power** (profiles 1 < 3) suggest that extreme-trauma survivors may experience more voice-related distress due to perceiving voices as persecutory or powerful. Results align with research linking severe trauma profiles to greater voice malevolence and omnipotence (Begemann et al., 2022) and with findings that many voice-hearers experience adverse voices alongside relational trauma (Marotti et al., 2025). While supporting the role of voice appraisals as key drivers of distress (Mawson, Cohen, & Berry, 2010; Tsang et al., 2021), findings suggest between-group differences in how childhood trauma profiles influence the appraisal–distress relationship, with the most traumatized individuals being more distressed by voice appraisals.

**Sleep disturbances’** between-group difference (profile 1 < 2) indicates a more prominent role of sleep in linking childhood trauma to voice-related distress among individuals with higher (but not extreme) trauma severity. Sensitivity analyses consistently supported sleep disturbances as the most robust mediator. This aligns with prior findings showing indirect effects of sleep disturbances on associations between childhood adversity and psychopathology (Laskemoen et al., 2021; Liu et al., 2023) and between interpersonal trauma and paranoia-related distress (Herms, Bolbecker, & Wisner, 2024). Although no difference emerged between profiles 2 and 3, sensitivity analyses treating trauma as a continuous variable of continuously identified sleep disturbances as a significant mediator. This suggests that they may function as a general pathway from trauma to voice-related distress, with its effects most detectable in the mid-range of trauma exposure, potentially reflecting non-linear trauma-sleep associations (Simon & Admon, 2023). Given that sleep disturbances are common in psychosis (Bagautdinova et al., 2023), predict subsequent psychotic experiences (Reeve, Sheaves, & Freeman, 2015), and reduce positive symptoms when treated (Scott et al., 2021), findings suggest that targeting sleep may offer a promising route to reducing voice-related distress, particularly for some trauma survivors. Sleep is increasingly recognized as a causal mechanism in psychosis (Freeman and Waite, 2025), particularly in paranoia (Brown et al., 2024), where sleep-focused interventions can reduce psychotic symptoms (Waite et al., 2023). Given bidirectional relationships between sleep and PTSD (Slavish et al., 2022) and between PTSD and psychosis (Buswell, Haime, Lloyd-Evans, & Billings, 2021; Hardy, 2017), future research could investigate whether trauma, sleep, and voice-related distress interact in reciprocal feedback loops.

Several null findings countered expectations and prior research. Results for **negative voice content** contrast prior findings linking it to a multi-trauma profile (Begemann et al., 2022) and mediating the association between childhood adversity and voice-related distress (Rosen et al., 2018), possibly reflecting methodological or sample differences. Result for **voice relating** oppose prior research linking it to distress (Hayward, Denney, Vaughan, & Fowler, 2008; León-Palacios et al., 2015; Rammou, Berry, Fowler, & Hayward, 2022; Sorrell, Hayward, & Meddings, 2010; Vaughan and Fowler, 2004), especially in patterns of low assertiveness (Schlier et al., 2021) or resistance and withdrawal (Hayward et al., 2016). Future studies might explore serial mediation or path models to test whether it emerges as a downstream process (Cole et al., 2017). For **emotion regulation**, results were similarly unexpected given the affective trauma-psychosis pathway (Grady, Twomey, Cullen, & Gaynor, 2024) and associations between voice-related distress and meta-worrying (Morrison and Wells, 2007) and rumination (Badcock, Paulik, & Maybery, 2011). Results contrast studies showing mediation between trauma and positive symptoms (Laloyaux et al., 2016) or positive symptom distress (Lincoln, Marin, & Jaya, 2017). However, the ERQ may be suboptimal for assessing emotion regulation in SSD, as it captures top-down regulatory processes, which can be impaired in this population (Lyu et al., 2025). For **gender**, results are not unprecedented (Laloyaux et al., 2016), but contrast suggested gender differences within some areas of psychosis (e.g. Giordano et al., 2021; Ochoa et al., 2012), voice-hearing (Murphy et al., 2010), voice relating (Hayward et al., 2016; Schlier et al., 2021), and emotional response to voices (Toh et al., 2020). Further research is needed to clarify gender-related trauma-psychosis pathways.

The null-finding of **depression** is consistent with prior findings of depression not mediating associations between childhood victimization profiles and positive symptoms (Barnes et al., 2023). However, in sensitivity analyses treating trauma as a continuous variable of continuously, depression emerged as having a significant indirect effect, aligning with evidence that depression could mediate links between negative schemas and voice-related distress (Kusztrits et al., 2022), though not between childhood adversity and voice-related distress (Rosen et al., 2018). These findings nuance the proposed affective pathway linking trauma and psychosis (Grady et al., 2024), by suggesting that it may be sensitive to how trauma is measured.

#### Trauma operationalization

Redefining exposure from profiles to the CTQ total score (Supplementary Material C Table 4) rendered statistically significant indirect effects for sleep disturbances and depression, but not for persecutory beliefs about voices or voice power. However, magnitudes were negligible and without clinical relevance (depression = 0.0031; sleep disturbances = 0.0035). These divergent findings highlight methodological considerations in trauma research, particularly concerning dose–response relationships (Flinn et al., 2025) versus the impact of specific trauma types (Grindey and Bradshaw, 2022). Modeling trauma exposure as a cumulative rather than a categorical profile membership implicitly assumes linear and equivalent effects across trauma types. However, childhood sexual abuse (CSA) has particularly severe developmental consequences and confers a substantially higher risk of PTSD than other trauma types (Boumpa et al., 2024; Dworkin, 2020; Dworkin, Jaffe, Bedard-Gilligan, & Fitzpatrick, 2023). Such effects may not be captured by continuous measures of accumulated trauma. While trauma profiles may better reflect patterns in the data, they may also mask trauma-type specificity, especially when experiences such as CSA occur across profiles. In this context, the null finding that trauma profiles were unrelated to voice-related distress is noteworthy. It may indicate that distress is similar across profiles and cannot be inferred from profile membership alone or that the profile-based approach obscures more specific associations between trauma types and distress, limiting its predictive utility.

### Implications

Results support the potential in identifying trauma profiles, their relationship to psychosis outcomes, and potential mediators to establish treatment targets. However, findings must be contextualized within the methodological heterogeneity in trauma research (Denton et al., 2017; Sætren et al., 2024). Variation in trauma measures, profile derivation and interpretation, and the operationalization of exposure and outcome in mediation models pose challenges for replicability and interpretation. Relationships between trauma and voice-related distress appear complex and non-deterministic, with multiple interacting pathways (e.g. equifinality and multifinality) shaping outcomes (e.g. Luhrmann et al., 2019). For clinical practice, findings (and the variability across studies) highlight the importance of incorporating developmental and trauma-focused perspectives into a multi-factor formulation, as in relational therapies (Thomas et al., 2025). Although LCA is a probabilistic, group-level method that does not translate directly to individual casework, it can point to mechanisms that may be especially important to consider in intervention. Still, this does not preclude non-mediators from being clinically relevant in individual cases. Relatedly, findings support future research examining whether LCA-derived trauma profiles act as treatment effect modifiers, given recent null findings for non-profile-based relational trauma in VR-based and standard cognitive behavioural therapy for paranoia (Christensen et al., 2026). For policymakers, findings support expanding access to trauma-informed care (Hardy et al., 2024) and prioritizing further research on such interventions (Bloomfield et al., 2020; Peters et al., 2022; Reid et al., 2024).

### Strengths and limitations

The study’s strengths include the OSF-preregistered protocol and adherence to AGReMA guidelines, enhancing transparency and reproducibility. Data were from the high-quality Challenge trial, which enrolled individuals broadly representative of the psychiatric clinic population, supporting generalizability.

A key limitation is the restricted variance across profiles, particularly in voice-related distress. This suggests that the association between childhood trauma and voice-related distress may be weaker than anticipated or that the help-seeking, treatment-resistant Challenge sample was already above a certain distress threshold. Constrained outcome variance limits the capacity to detect mediated effects, rendering the analyses conservative and potentially obscuring differences that might emerge in a less clinically homogeneous sample, as similarly noted in a delusion-focused study (Barnes et al., 2023).

SEM analyses assess between-group differences and cannot determine whether mechanisms operate similarly within trauma profiles. The assumed causal model included few confounders, leaving open the possibility of unmeasured confounding. Relevant factors were uncontrolled due to data unavailability (e.g. ethnicity, socioeconomic status). Potential colliders were not included in the model and represent an avenue for future research. Potentially relevant mechanisms such as attachment, dissociation, PTSD symptoms (Bloomfield et al., 2021), shame (Davies et al., 2025), or cannabis consumption (Hasan et al., 2020) were not examined. Future research could examine mediators directly as explanatory variables, using standard regression independent of trauma, to assess their association with voice-related distress.

Although the sample was large for a clinical trial, the size may have been insufficient for reliable LCA estimation, potentially affecting results. Interpretation is constrained by definitional and measurement limitations surrounding childhood trauma (Denton et al., 2017). Only CTQ was used, excluding alternative operationalizations and trauma occurring outside the family or within adolescence and adulthood (e.g. peer bullying, discrimination, psychosis-related events). The Danish version may introduce comprehension issues, as also observed in the Dutch version (Thombs et al., 2009). Further, CTQ only measures traumatic events, not traumatic experiences or traumatic effects (Samhsa, 2014).

Finally, the cross-sectional analysis of a retrospective trauma assessment cannot determine the causal nature of any relations, as well as their direction. This limitation is inherent to the trauma-psychosis field given the nature of trauma and the challenges of conducting longitudinal studies.

## Conclusions

This study is, to our knowledge, the first to demonstrate evidence of between-group differences in mediators linking childhood trauma profiles to voice-related distress in a sample of clinical voice-hearers. Results suggest childhood trauma profiles can provide indicators of mediators that may be relevant in informing individualized formulation and therapy planning. While persecutory beliefs about voices, voice power, and sleep disturbances will be relevant treatment targets for many voice-hearers, this study emphasize that, for some, these experiences contribute to voice-related distress because of their roots in childhood trauma.

## Supporting information

10.1017/S0033291726104437.sm001 Christensen et al. supplementary material Christensen et al. supplementary material

## Data Availability

Data from the Challenge trial will be made publicly available in the Danish National Archives following initial publications. Code will be made available through OSF.

## Supplementary Material

The supplementary material for this article can be found at http://doi.org/10.1017/S0033291726104437.

## Long descriptions

### Table 1. Long description

From the top row, columns are analytic role, variable, measure, and additional details. The first row lists exposure as childhood trauma, measured by the Childhood Trauma Questionnaire–Short Form, a 28-item self-report assessing abuse and neglect with five subscales and a total score. The outcome is voice distress, measured by the Psychotic Symptom Rating Scale–Auditory Hallucinations Subscale, using a distress-items sum score. Mediator variables are grouped with conceptual and action theory and measure columns. Negative voice content is linked to trauma and distress, measured by PSYRATS-AHS negative content-item sum score. Persecutory beliefs about voices are associated with trauma and distress, measured by the Beliefs about Voices Questionnaire–Revised, using the Persecutory Beliefs Factor. Voice power is measured by the Voice Power Differential Scale, with higher scores indicating greater perceived voice power. Relating to voices is shaped by trauma, measured by Approve-Voice, with subscales for assertive, aggressive, and passive relating. Negative self/other beliefs are associated with trauma and attachment, measured by the Brief Core Schema Scale negative subscales. Emotion regulation difficulties are linked to trauma, measured by the Emotion Regulation Questionnaire, yielding cognitive reappraisal and expressive suppression subscales. Depression is associated with trauma, measured by the Calgary Depression Scale for Schizophrenia. Sleep disturbances are linked to trauma and psychosis, measured by the Pittsburgh Sleep Quality Index, using 19 self-report items for a global score. The moderator is gender, measured by self-report, with noted differences in psychosis outcomes. The confounder is age, extracted from case reports. The table footnote refers to Supplementary Material A for details.

Navigate back to Table 1..

### Table 2. Long description

The table has five columns: Profile, 1 Variable trauma, 2 Severe neglect and emotional abuse, 3 Severe poly-trauma, Total, and Missings/N (percent). From the top row downward: Total participants are 160 (60.2 percent) in profile 1, 84 (31.6 percent) in profile 2, 22 (8.3 percent) in profile 3, and 266 (100 percent) overall, with no missing data. Gender: Female counts are 91 (56.9 percent), 58 (69.0 percent), 14 (63.6 percent), and 163 (61.3 percent) by profile; Male counts are 69 (43.1 percent), 26 (31.0 percent), 8 (36.4 percent), and 103 (38.7 percent) with no missing data. Age mean (standard deviation): 32.7 (12.1), 32.7 (11.6), 34.0 (11.3), and 32.8 (11.8). Education: Primary 61 (38.1 percent), 36 (42.9 percent), 7 (31.8 percent), 104 (39.1 percent); Secondary 42 (26.2 percent), 27 (32.1 percent), 9 (40.9 percent), 78 (29.3 percent); Vocational 34 (21.2 percent), 14 (16.7 percent), 3 (13.6 percent), 51 (19.2 percent); University 16 (10.0 percent), 6 (7.1 percent), 1 (4.5 percent), 23 (8.6 percent); Other 7 (4.4 percent), 1 (1.2 percent), 2 (9.1 percent), 10 (3.8 percent). Occupation: Full-time 3 (1.9 percent), 2 (2.4 percent), 1 (4.5 percent), 6 (2.3 percent); Part-time 14 (8.8 percent), 6 (7.1 percent), 0, 20 (7.5 percent); Student 20 (12.5 percent), 8 (9.5 percent), 4 (18.2 percent), 32 (12.0 percent); Stay-at-home 0 in all profiles; Unemployed 53 (33.1 percent), 39 (46.4 percent), 5 (22.7 percent), 97 (36.5 percent); Retired/early retirement/post-employment 57 (35.6 percent), 23 (27.4 percent), 11 (50.0 percent), 91 (34.2 percent); Other 13 (8.1 percent), 6 (7.1 percent), 1 (4.5 percent), 20 (7.5 percent). Years with voice-hearing mean (standard deviation): 14.7 (11.5), 17.0 (10.4), 19.0 (9.6), 15.8 (11.1), with 8 missing (3.0 percent). Attending a voice-hearing group outside psychiatric services: No 155 (96.9 percent), 80 (96.4 percent), 21 (95.5 percent), 256 (96.6 percent); Yes 5 (3.1 percent), 3 (3.6 percent), 1 (4.5 percent), 9 (3.4 percent), with 1 missing (0.4 percent). Any antipsychotics use: 148 (92.5 percent), 73 (86.9 percent), 19 (86.4 percent), 240 (90.2 percent). Dosage of olanzapine equivalent (mg/day, standard deviation): 20.69 (14.59), 16.07 (12.22), 18.43 (11.90), 19.04 (13.78), with 6 missing (2.3 percent). First-generation antipsychotic use: 20 (12.5 percent), 8 (9.5 percent), 2 (9.1 percent), 30 (11.3 percent). Clozapine use: 45 (28.1 percent), 12 (14.3 percent), 4 (18.2 percent), 61 (22.9 percent). Non-sedative antipsychotic use: 82 (51.2 percent), 43 (51.2 percent), 13 (59.1 percent), 138 (51.9 percent). Sedative antipsychotic use: 55 (34.4 percent), 26 (31.0 percent), 9 (40.9 percent), 90 (33.8 percent). Diagnosis (ICD-10): Schizophrenia (F20) 149 (93.1 percent), 77 (91.7 percent), 19 (86.4 percent), 245 (92.1 percent); Persistent delusional disorders (F22.8/F22.9) 3 (1.9 percent), 1 (1.2 percent), 0, 4 (1.5 percent); Schizoaffective disorders (F25) 3 (1.9 percent), 2 (2.4 percent), 1 (4.5 percent), 6 (2.3 percent); Other non-organic psychotic disorders (F28 and F29) 5 (3.1 percent), 4 (4.8 percent), 2 (9.1 percent), 11 (4.1 percent). Childhood trauma (total CTQ) mean (standard deviation): 36.62 (7.92), 63.93 (10.00), 92.05 (15.25), 49.83 (20.09). Emotional abuse mean (standard deviation): 8.75 (3.21), 18.40 (3.62), 21.95 (3.53), 12.89 (6.17). None: 57 (35.6 percent), 0, 0, 57 (21.4 percent); Low: 36 (22.5 percent), 0, 0, 36 (13.5 percent); Moderate: 27 (16.9 percent), 0, 0, 27 (10.2 percent); Severe: 40 (25.0 percent), 84 (100.0 percent), 22 (100.0 percent), 146 (54.9 percent). Physical abuse mean (standard deviation): 5.46 (1.27), 7.35 (2.35), 19.59 (3.84), 7.22 (4.29). None: 130 (81.2 percent), 30 (35.7 percent), 0, 160 (60.2 percent); Moderate: 13 (8.1 percent), 10 (11.9 percent), 0, 23 (8.6 percent); Severe: 17 (10.6 percent), 44 (52.4 percent), 22 (100.0 percent), 83 (31.2 percent). Sexual abuse mean (standard deviation): 6.42 (3.78), 9.45 (5.94), 15.27 (8.24), 8.11 (5.62). None: 129 (80.6 percent), 39 (46.4 percent), 4 (18.2 percent), 172 (64.7 percent); Moderate: 5 (3.1 percent), 4 (4.8 percent), 1 (4.5 percent), 10 (3.8 percent); Severe: 26 (16.2 percent), 41 (48.8 percent), 17 (77.3 percent), 84 (31.6 percent). Emotional neglect mean (standard deviation): 9.36 (3.62), 17.54 (4.14), 19.32 (4.82), 12.76 (5.73). None: 83 (51.9 percent), 6 (7.1 percent), 0, 89 (33.5 percent); Low: 41 (25.6 percent), 0, 3 (13.6 percent), 44 (16.5 percent); Moderate: 15 (9.4 percent), 8 (9.5 percent), 0, 23 (8.6 percent); Severe: 21 (13.1 percent), 70 (83.3 percent), 19 (86.4 percent), 110 (41.4 percent). Physical neglect mean (standard deviation): 6.64 (2.11), 11.19 (3.86), 15.91 (5.19), 8.84 (4.28). None: 72 (45.0 percent), 3 (3.6 percent), 0, 75 (28.2 percent); Low: 26 (16.2 percent), 4 (4.8 percent), 1 (4.5 percent), 31 (11.7 percent); Moderate: 19 (11.9 percent), 5 (6.0 percent), 0, 24 (9.0 percent); Severe: 43 (26.9 percent), 72 (85.7 percent), 21 (95.5 percent), 136 (51.1 percent). Voice-related distress mean (standard deviation): 6.34 (1.30), 6.30 (1.29), 6.59 (1.33), 6.35 (1.30). Negative voice-content mean (standard deviation): 6.88 (1.36), 6.86 (1.35), 7.14 (1.04), 6.89 (1.33). Persecutory belief factor mean (standard deviation): 17.20 (6.14), 17.63 (6.13), 20.00 (5.64), 17.56 (6.12). Voice power differential mean (standard deviation): 24.75 (5.34), 25.52 (4.37), 26.82 (4.36), 25.16 (5.00). Assertive relating mean (standard deviation): 22.08 (13.29), 20.86 (14.61), 25.86 (15.34), 22.01 (13.90). Aggressive responding mean (standard deviation): 14.75 (12.22), 18.02 (14.63), 26.00 (17.65), 16.68 (13.81). Passive/submissive responding mean (standard deviation): 22.93 (14.28), 23.59 (12.86), 28.93 (15.12), 23.62 (13.95). Negative other-beliefs mean (standard deviation): 6.65 (5.77), 7.40 (5.77), 7.36 (6.77), 6.95 (5.85). Negative self-beliefs mean (standard deviation): 10.12 (5.75), 11.52 (6.26), 12.36 (5.46), 10.76 (5.93). Cognitive reappraisal mean (standard deviation): 23.39 (7.61), 22.49 (8.94), 23.27 (8.76), 23.10 (8.13). Expressive suppression mean (standard deviation): 16.34 (5.58), 16.38 (5.44), 15.73 (6.08), 16.30 (5.56). Depression mean (standard deviation): 6.08 (4.38), 6.66 (4.76), 8.23 (5.25), 6.44 (4.60). Sleep disturbances mean (standard deviation): 9.59 (4.29), 11.36 (3.95), 11.38 (4.25), 10.30 (4.25).

Navigate back to Table 2..

### Figure 2. Long description

At the center are concentric rings marking mean scores from 0 to 25. Five axes radiate outward labeled clockwise as Physical abuse, Sexual abuse, Emotional abuse, Emotional neglect, and Physical neglect. Three colored polygons represent trauma profiles: blue for Profile 1 (n equals 160, Variable severity), orange for Profile 2 (n equals 84, Severe neglect and emotional abuse), and green for Profile 3 (n equals 22, Severe poly-trauma). Profile 1 forms the smallest polygon, with mean scores near 5 on all axes. Profile 2 forms a larger polygon, with highest values on Emotional neglect and Emotional abuse, moderate on Physical neglect, and lower on Physical and Sexual abuse. Profile 3 forms the largest polygon, with mean scores near or above 15 on all axes, peaking at Physical abuse. The legend below the chart identifies each profile by color and description.

Navigate back to Figure 2..

### Figure 3. Long description

The horizontal forest plot displays mean scores with 95 percent confidence intervals for Profile 1 (blue circle), Profile 2 (orange square), and Profile 3 (green x) for each variable. From top to bottom, the y-axis lists P S Y R A T S dash N V C, P S Y R A T S dash D I S, B A V Q dash R dash P B, V P D S, A P P R O V E dash A S, A P P R O V E dash A G, A P P R O V E dash P S, B C S S dash O N, B C S S dash S N, E R Q dash C R, E R Q dash E S, C D S S, and P S Q I. The x-axis shows mean score values, with scale ranges indicated by dashed lines. For most variables, Profile 3 (green x) has higher mean scores than Profiles 1 and 2, especially for B A V Q dash R dash P B, V P D S, and A P P R O V E variables. P S Y R A T S variables are plotted on a smaller scale at the top, with all profiles showing higher means. The legend at the bottom identifies the symbols for each profile.

Navigate back to Figure 3..

### Table 3. Long description

The table is organized by hypothesis, each spanning three rows for class comparisons: 2 vs 1, 3 vs 1, and 3 vs 2. Columns are: Class, N, Indirect, 95 percent confidence interval lower and upper, p, Direct, 95 percent confidence interval lower and upper, p, Total, 95 percent confidence interval lower and upper, p. For H1 Negative voice-content, all effects are non-significant with p-values above 0.05. For H2 Persecutory belief factor, the indirect effect for 3 vs 1 is 0.265 with a 95 percent confidence interval from 0.003 to 0.538 and p equals 0.048, which is significant. For H3 Voice power differential, the indirect effect for 3 vs 1 is 0.175 with a 95 percent confidence interval from 0.007 to 0.377 and p equals 0.041, also significant. For H4 Assertive relating, all p-values are non-significant. For H5 Aggressive responding, all p-values are non-significant. For H6 Passive/submissive responding, all p-values are non-significant. For H7 Negative other-beliefs, all p-values are non-significant. For H8 Negative self-beliefs, all p-values are non-significant. For H9 Cognitive reappraisal, all p-values are non-significant. For H10 Expressive suppression, all p-values are non-significant. For H11 Depression, all p-values are non-significant. For H12 Sleep disturbances, the direct effect for 2 vs 1 is negative 0.124 with a 95 percent confidence interval from negative 0.462 to 0.219 and p equals 0.002, which is significant. All confidence intervals are at 95 percent. Class 1 is low or moderate trauma, class 2 is multi-trauma, class 3 is extreme multi-trauma. Measures include Approve, B A V Q dash R, C T Q, B C S S, C D S S, E R Q, P S Q I, and V P D S.

Navigate back to Table 3..

### Table 4. Long description

The table presents differences in indirect effect by gender (female vs male) for 12 hypotheses, each corresponding to a psychological or clinical construct. For each hypothesis, three class comparisons are shown: 2 vs 1, 3 vs 1, and 3 vs 2. Columns display the estimate of difference, 95 percent confidence interval lower and upper bounds, and P value. H1 Negative voice-content (items 6 plus 7 PSYRATS-AHS): 2 vs 1 estimate negative 0.442, CI negative 0.964 to 0.010, P 0.057; 3 vs 1 estimate negative 0.359, CI negative 1.078 to 0.199, P 0.232; 3 vs 2 estimate 0.083, CI negative 0.642 to 0.744, P 0.796. H2 Persecutory belief factor (subscale BAVQ-R): 2 vs 1 estimate 0.167, CI negative 0.144 to 0.508, P 0.308; 3 vs 1 estimate negative 0.068, CI negative 0.644 to 0.481, P 0.796; 3 vs 2 estimate negative 0.234, CI negative 0.857 to 0.348, P 0.414. H3 Voice power differential (total VPDS): 2 vs 1 estimate negative 0.151, CI negative 0.386 to 0.046, P 0.144; 3 vs 1 estimate 0.113, CI negative 0.212 to 0.443, P 0.470; 3 vs 2 estimate 0.264, CI negative 0.057 to 0.619, P 0.100. H4 Assertive relating (subscale Approve): 2 vs 1 estimate 0.059, CI negative 0.071 to 0.202, P 0.374; 3 vs 1 estimate negative 0.088, CI negative 0.408 to 0.148, P 0.450; 3 vs 2 estimate negative 0.146, CI negative 0.478 to 0.091, P 0.228. H5 Aggressive responding (subscale Approve): 2 vs 1 estimate 0.050, CI negative 0.057 to 0.264, P 0.446; 3 vs 1 estimate 0.153, CI negative 0.196 to 0.605, P 0.384; 3 vs 2 estimate 0.103, CI negative 0.185 to 0.472, P 0.511. H6 Passive/submissive responding (subscale Approve): 2 vs 1 estimate 0.051, CI negative 0.128 to 0.242, P 0.574; 3 vs 1 estimate 0.102, CI negative 0.339 to 0.512, P 0.562; 3 vs 2 estimate 0.051, CI negative 0.413 to 0.457, P 0.732. H7 Negative other-beliefs (subscale B C S S): 2 vs 1 estimate 0.020, CI negative 0.051 to 0.113, P 0.549; 3 vs 1 estimate 0.061, CI negative 0.132 to 0.268, P 0.547; 3 vs 2 estimate 0.040, CI negative 0.152 to 0.232, P 0.669. H8 Negative self-beliefs (subscale B C S S): 2 vs 1 estimate 0.014, CI negative 0.166 to 0.189, P 0.798; 3 vs 1 estimate 0.005, CI negative 0.283 to 0.271, P 0.899; 3 vs 2 estimate negative 0.009, CI negative 0.299 to 0.249, P 0.977. H9 Cognitive reappraisal (subscale E R Q): 2 vs 1 estimate 0.017, CI negative 0.093 to 0.134, P 0.706; 3 vs 1 estimate 0.095, CI negative 0.106 to 0.366, P 0.356; 3 vs 2 estimate 0.078, CI negative 0.152 to 0.372, P 0.502. H10 Expressive suppression (subscale E R Q): 2 vs 1 estimate 0.042, CI negative 0.083 to 0.188, P 0.522; 3 vs 1 estimate negative 0.042, CI negative 0.266 to 0.159, P 0.634; 3 vs 2 estimate negative 0.084, CI negative 0.341 to 0.144, P 0.429. H11 Depression (total C D S S): 2 vs 1 estimate negative 0.039, CI negative 0.268 to 0.163, P 0.697; 3 vs 1 estimate 0.136, CI negative 0.245 to 0.527, P 0.458; 3 vs 2 estimate 0.175, CI negative 0.215 to 0.579, P 0.344. H12 Sleep disturbances (total P S Q I): 2 vs 1 estimate 0.025, CI negative 0.215 to 0.231, P 0.744; 3 vs 1 estimate 0.137, CI negative 0.144 to 0.418, P 0.292; 3 vs 2 estimate 0.112, CI negative 0.159 to 0.417, P 0.409. No P values are below 0.05, indicating no statistically significant differences in indirect effect by gender across all hypotheses and class comparisons.

Navigate back to Table 4..

### Figure 1. Long description

The flowchart consists of five rounded nodes connected by solid and dotted arrows.

* On the far left, the Exposure node contains Childhood trauma profile.

* Above and to the left, the Confounder node lists Age, Medication category asterisk, and Years with voices asterisk.

* In the center, the Mediator node lists Negative voice-content, Persecutory belief, voice, Voice power, Relating to voices, Neg. self/other beliefs, Emotion regulation, Depression, and Sleep disturbances.

* At the bottom center, the Moderator node contains Gender.

* On the far right, the Outcome node contains Voice-related distress.

Causal pathways are as follows.

* A horizontal arrow labeled a points from Exposure to the Mediator.

* A horizontal arrow labeled b points from the Mediator to the Outcome.

* A horizontal arrow labeled c prime points directly from Exposure to the Outcome.

* The Confounder node has two branching arrows. One points down to the path between Exposure and Mediator. The other points down to the path between Mediator and Outcome.

* The Moderator node has three branching arrows. One solid arrow points up to the path between Exposure and Mediator. A second solid arrow points up to the path between Mediator and Outcome. A third dotted arrow points up to the direct path c prime.

Navigate back to Figure 1..
