# Supplementary material for: Psychological and behavioral mechanisms linking childhood trauma profiles to voice-related distress in schizophrenia spectrum disorders: latent class, mediation, and conditional process analyses
Source: Psychol Med. 2026 Jun 8;56:e188. doi: 10.1017/S0033291726104437 (PMC13247800; doi:10.1017/S0033291726104437)
Supplement: Christensen et al. supplementary material [file S0033291726104437sup001.docx]

Supplementary Materials

[Supplementary Material A: Conceptual and action theories for candidate mediators 2](#_Toc225513331)

[Supplementary Material B: Sensitivity parameters 6](#_Toc225513332)

[Analyses 6](#_Toc225513333)

[Outcomes 6](#_Toc225513334)

[Supplementary Material C: Supplement Tables 8](#_Toc225513335)

[References 40](#_Toc225513336)

# Supplementary Material A: Conceptual and action theories for candidate mediators

Drawing on existing theories and research on trauma in psychosis, we propose that negative voice content, negative self/other beliefs, voice appraisals, voice relating, emotion regulation, depression, and sleep disturbances may mediate the association between childhood PTEs and voice-related distress, while gender may act as a moderator. These hypotheses are informed by the following conceptual theories (linking exposure to mediators) and action theories (linking mediators to outcomes):

**Negative voice content** is associated with childhood PTEs (Begemann et al., 2022) and can be influenced by several factors (Larøi et al., 2019), including the hearer’s life history (Corstens & Longden, 2013; Hardy et al., 2005; Longden et al., 2012; Reiff et al., 2012; Rosen et al., 2018). It is considered a key driver of voice-related distress (Copolov et al., 2004; Larøi et al., 2019; Silver et al., 2023), can predict engagement with mental health services (Beavan & Read, 2010; Daalman et al., 2011), and most individuals with SSD report predominantly negative voices (McCarthy-Jones et al., 2014; Nayani & David, 1996).

**Negative self/other core beliefs** can mediate the relationship between PTEs and psychotic-like symptoms in non-clinical samples, as well as PTEs and positive symptoms in SSD (Jorovat et al., 2025). These beliefs have been predicted by childhood emotional trauma and insecure-anxious attachment (M. Scott et al., 2020), with sexual, physical, or emotional abuse specifically associated with more severe negative-other beliefs (Hardy et al., 2016). Trauma-related beliefs are associated with hallucinations and delusions (Frost et al., 2024), and negative core beliefs can shape voice appraisals as malevolent, persecutory, powerful, or critical, which are associated with voice-related distress (Birchwood et al., 2000; Larøi et al., 2019; Thomas et al., 2015). One study found that attachment style predicted negative self/other beliefs, which in turn predicted negative beliefs about voices, which predicted voice-related distress (Cole et al., 2017). Similarly, in patients with non-affective psychosis, greater depression, lower self-esteem, and more negative self-beliefs were associated with voice-related distress (Smith et al., 2006).

**Voice appraisals** may be shaped by self/other core beliefs (Birchwood et al., 2004), with negative self-beliefs linked to negative beliefs about voices in SSD (Thomas et al., 2015). As these beliefs develop early (Beck, 2005; Beck & Rector, 2005), they are plausibly influenced by attachment and trauma (Berry et al., 2008, 2012; Hardy et al., 2016; Jorovat et al., 2025; Williams et al., 2018). Supporting this, studies have shown that emotional trauma, insecure attachment, and negative self-schemas jointly predicted negative voice content (M. Scott et al., 2020), and that trauma predicted variance in malevolence, benevolence, and omnipotence voice appraisals (Andrew et al., 2008). Voice appraisals are considered key drivers of distress and depression (Mawson et al., 2010), particularly beliefs about malevolence, omnipotence, social rank, power, and expressed emotion (Birchwood et al., 2000; Chadwick & Birchwood, 1994; Connor & Birchwood, 2013). Appraisals predict voice-related distress (Cole et al., 2017; Peters et al., 2012) and are associated with general distress, depression, and behavioral responses independent of voice activity (Birchwood et al., 2004). Meta-analytic evidence confirms robust links between distress and appraisals of malevolence, power, dominance, intrusiveness, and metaphysical beliefs or beliefs about loss of control (Tsang et al., 2021).

**Relating to voices** can mirror social relationships (Hayward, 2003; Hayward et al., 2011, 2020), reflecting broader interpersonal patterns (Birchwood et al., 2000; Mawson et al., 2011; O’Brien et al., 2021; Paulik, 2012; Ward et al., 2022) shaped by life history and trauma (Corstens et al., 2014; Corstens & Longden, 2013; Longden et al., 2012; Ward et al., 2020). More passive and less assertive relating increase voice-related distress, particularly among women (Schlier et al., 2021), who may show greater resistance and withdrawal (Hayward et al., 2016). Perceived voice dominance and intrusiveness are associated with distress in young people (Rammou et al., 2022) and adults (Hayward et al., 2008; León-Palacios et al., 2015; Sorrell et al., 2010; Vaughan & Fowler, 2004), with hostility, power, and persecutory beliefs in relating to voices influencing the association between relating style and negative affect (León-Palacios et al., 2015; Rammou et al., 2022; Sorrell et al., 2010; Thomas et al., 2009).

**Emotion regulation difficulties** are associated with childhood adversity (Gruhn & Compas, 2020; Lavi et al., 2019; Miu et al., 2022), and may partly mediate the relation between adversity and psychopathology (Laloyaux et al., 2016; Miu et al., 2022). Emotion regulation difficulties are common in the SSD population (Chapman et al., 2019; Moran et al., 2018). People with psychosis use more maladaptive and fewer adaptive regulation strategies than healthy controls, including greater rumination, self-blame, and suppression, and less distraction or reappraisal (Ludwig et al., 2019; O’Driscoll et al., 2014). This pattern is clinically relevant, as reappraisal compared to suppression is associated with better outcomes (Cutuli, 2014), while maladaptive strategies like meta-worrying (Morrison & Wells, 2007) and rumination (Badcock et al., 2011) are linked to voice-related distress. Furthermore, a prospective study found emotion regulation partially mediated the relation between childhood interpersonal trauma and positive symptom distress, but not symptom severity (Lincoln et al., 2017).

**Depression** may increase following trauma (Schindel-Allon et al., 2010), with childhood sexual abuse contributing an estimated 13% to the global population attributable fraction (Dragioti et al., 2022), and cumulative childhood adversities predicting higher depression in women with SSD or bipolar disorder (Köhler-Forsberg et al., 2024). Depression and anxiety may shape psychosis through processes such as beliefs about deserving harm (Freeman & Garety, 2014), and are associated with psychotic symptom severity, content, and distress (Hartley et al., 2013). Depression has been shown to mediate negative schemas and voice-related distress (Kusztrits et al., 2022) but neither childhood adversities and voice-related distress (Rosen et al., 2018) nor childhood victimization profiles and positive symptoms (Barnes et al., 2023).

**Sleep disturbances** are associated with childhood adversities (Schønning et al., 2022; Vadukapuram et al., 2022), are common in psychosis (Bagautdinova et al., 2023), may predict subsequent psychotic experiences (Reeve et al., 2015), reduce positive symptoms when treated (A. J. Scott et al., 2021), and have been shown to mediate the associations between childhood adversities and psychopathology (Laskemoen et al., 2021; Liu et al., 2023), including interpersonal trauma and paranoia-related distress (Herms et al., 2024). While not directly tied to voice-related distress, one study found spindle activity deficits in SSD patients both with and without voices, with several deficits more pronounced in the voice-hearing group (Sun et al., 2023).

**Gender differences** exist in psychosis (Carter et al., 2022; Giordano et al., 2021; Ochoa et al., 2012), with women reporting greater voice severity, more distress, and more resistant relating styles than men (Hayward et al., 2016; Murphy et al., 2010; Schlier et al., 2021; Toh et al., 2020). Trauma-psychosis associations also appear stronger in women, especially following neglect (Garcia et al., 2016), potentially due to sex differences in hypothalamic-pituitary-adrenal (HPA) axis reactivity (Desantis et al., 2011; Gibson et al., 2014; Kelly et al., 2016). Nonetheless, evidence for gender as a moderator remains mixed (Laloyaux et al., 2016).

# Supplementary Material B: Sensitivity parameters

## Analyses

In accordance with the preregistered protocol (<https://osf.io/s6dtf>), confounder adjustment and sensitivity analyses were performed to explore assumptions in the mediation model, with the Benjamini-Hochberg procedure applied to control the false-positive rate (Thissen et al., 2002). First, adjustment for age as a potential confounder was conducted in all exposure–outcome and exposure–mediator regressions. Three age categories derived from tertiles of the full dataset were used. Second, all mediation analyses were re-estimated after excluding participants with CTQ minimization/denial scores >0. Third, analyses were repeated treating childhood trauma as a continuous variable (CTQ total score), disregarding LCA groupings but aligning with common practice. Fourth, alternative operationalizations of voice-related distress were tested to avoid methodological artifacts. These included combining PSYRATS negative content and distress items (Drake et al., 2007) with either the control item (Woodward et al., 2014) or the disruption item (Steel et al., 2007), as well as analyzing distress and negative content items separately. When negative content items were used as outcomes, models using negative voice content as a mediator were omitted to avoid redundancy. Following reviewer suggestions, post hoc sensitivity analyses incorporated antipsychotic class (first-generation, clozapine, non-sedating second-generation, sedating second-generation) and duration of voice hearing (tertiles) as potential confounders. Medication classes included: First-generation antipsychotics (Chlorprothixene, Haloperidol, Perphenazine, Zuclopenthixol); Clozapine; Non-sedating antipsychotics (Aripiprazole, Amisulpride, Lurasidone); Sedating antipsychotics (Olanzapine, Quetiapine).

## Outcomes

All outcomes for mediation and conditional process analyses adjusted for age are provided in Supplementary Material C Tables 1 and 2, respectively. Adjusting for age revealed a significant (profiles 1 vs. 3) and a near-significant effect (profiles 1 vs. 2) indirect effect of negative self-beliefs. No moderation by gender was found.

Excluding participants scoring >0 on the CTQ minimization/denial subscale made the indirect effect of voice persecutory beliefs (profiles 1 vs. 3) non-significant, while effects of voice power differential (profiles 1 vs. 3) and sleep disturbances (profiles 1 vs. 2) remained. However, none survived correction for multiple testing (Supplementary Material C Table 3).

Excluding participants scoring >0 on the CTQ minimization/denial subscale rendered the indirect effect of persecutory beliefs about voices (profiles 1 vs. 3) non-significant, while effects of voice power differential (profiles 1 vs. 3) and sleep disturbances (profiles 1 vs. 2) remained. However, none survived correction for multiple testing (Supplementary Material C Table 3).

Redefining exposure from categorical childhood trauma profiles to the CTQ total score, aggregating the five trauma subscales into a single continuous variable, yielded significant indirect effects for negative self-beliefs, sleep disturbances, and depression, but not for persecutory beliefs about voices or voice power. Only sleep disturbances and depression survived correction for multiple testing (Supplementary Material C Table 4).

Alternative operationalizations of voice-related distress did not change the main findings for persecutory beliefs about voices (profiles 1 vs. 3), voice power (profiles 1 vs. 3), and sleep disturbances (profiles 1 vs. 2). However, only sleep disturbances survived correction for multiple testing (Supplementary Material C Tables 5-10).

Including antipsychotic class and duration of voice hearing as potential confounders did not change the main findings for persecutory beliefs about voices (profiles 1 vs. 3), voice power (profiles 1 vs. 3), and sleep disturbances (profiles 1 vs. 2) (Supplementary Material C Tables 12-15).

# Supplementary Material C: Supplement Tables

**Supplement 1**. Age-adjusted point estimates and uncertainty estimates for total, direct, and indirect effects.

**Supplement 2.** Age-adjusted point estimates and uncertainty estimates for differences in indrect effect according to gender (female VS male).

**Supplement 3.** Point estimates and uncertainty estimates for total, direct, and indirect effects after exclusion of individuals with CTQ validity score >0 on the minimization and denial scale

**Supplement 4.** Point estimates and uncertainty estimates for total, direct, and indirect effects treating trauma exposure as continuous CTQ score.

**Supplement 5.** Point estimates and uncertainty estimates for total, direct, and indirect effects operationalizing outcome as sum score of PSYRATS-AH items 6,7,8,9,10.

**Supplement 6.** Point estimates and uncertainty estimates for total, direct, and indirect effects operationalizing outcome as sum score of PSYRATS-AH items 6,7,8,9,11.

**Supplement 7.** Point estimates and uncertainty estimates for total, direct, and indirect effects operationalizing outcome as sum score of PSYRATS-AH items 6,7,8,9.

**Supplement 8**. Point estimates and uncertainty estimates for total, direct, and indirect effects operationalizing outcome as sum score of PSYRATS-AH items 8,9,11.

**Supplement 9**. Point estimates and uncertainty estimates for total, direct, and indirect effects operationalizing outcome as sum score of PSYRATS-AH item 8.

**Supplement 10.** Point estimates and uncertainty estimates for total, direct, and indirect effects operationalizing outcome as sum score of PSYRATS-AH item 9.

**Supplement 11.** Statistical fit of latent class analysis, AIC and BIC values.

**Supplement 12.** Medication-adjusted point estimates and uncertainty estimates for total, direct, and indirect effects.

**Supplement 13.** Medication-adjusted point estimates and uncertainty estimates for differences in indrect effect according to gender (female VS male).

**Supplement 14.** Voice duration-adjusted point estimates and uncertainty estimates for total, direct, and indirect effects.

**Supplement 15.** Voice duration-adjusted point estimates and uncertainty estimates for differences in indrect effect according to gender (female VS male).

| **Supplement 1.** Age-adjusted point estimates and uncertainty estimates for total, direct, and indirect effects. | | | | | | | | | | | | | | |
| --- | --- | --- | --- | --- | --- | --- | --- | --- | --- | --- | --- | --- | --- | --- |
|  | Class | N | Indirect | Lower | Upper | P | Direct | Lower | Upper | P | Total | Lower | Upper | P |
| H1 Negative voice-content (items 6+7 PSYRATS-AHS) | 2 VS 1 | 266 | -0.009 | -0.250 | 0.209 | 0.949 | -0.038 | -0.300 | 0.229 | 0.782 | -0.047 | -0.398 | 0.286 | 0.798 |
|  | 3 VS 1 | 266 | 0.171 | -0.173 | 0.484 | 0.289 | 0.062 | -0.385 | 0.490 | 0.743 | 0.233 | -0.417 | 0.786 | 0.415 |
|  | 3 VS 2 | 266 | 0.180 | -0.175 | 0.515 | 0.282 | 0.100 | -0.352 | 0.541 | 0.634 | 0.280 | -0.388 | 0.854 | 0.357 |
| H2 Persecutory belief factor (subscale BAVQ-R) | 2 VS 1 | 265 | 0.050 | -0.104 | 0.207 | 0.530 | -0.097 | -0.422 | 0.205 | 0.542 | -0.048 | -0.400 | 0.287 | 0.797 |
|  | 3 VS 1 | 265 | 0.283 | 0.007 | 0.581 | **0.041** | -0.112 | -0.682 | 0.388 | 0.710 | 0.171 | -0.477 | 0.750 | 0.546 |
|  | 3 VS 2 | 265 | 0.233 | -0.040 | 0.539 | 0.093 | -0.015 | -0.617 | 0.526 | 0.993 | 0.219 | -0.449 | 0.812 | 0.464 |
| H3 Voice power differential (total VPDS) | 2 VS 1 | 263 | 0.073 | -0.038 | 0.189 | 0.195 | -0.091 | -0.418 | 0.228 | 0.574 | -0.018 | -0.357 | 0.312 | 0.916 |
|  | 3 VS 1 | 263 | 0.191 | 0.015 | 0.407 | **0.032** | 0.047 | -0.510 | 0.531 | 0.805 | 0.237 | -0.371 | 0.793 | 0.393 |
|  | 3 VS 2 | 263 | 0.118 | -0.055 | 0.319 | 0.178 | 0.137 | -0.445 | 0.650 | 0.588 | 0.256 | -0.368 | 0.833 | 0.390 |
| H4 Assertive relating (subscale Approve) | 2 VS 1 | 265 | 0.026 | -0.043 | 0.108 | 0.448 | -0.114 | -0.441 | 0.219 | 0.504 | -0.087 | -0.418 | 0.244 | 0.616 |
|  | 3 VS 1 | 265 | -0.060 | -0.207 | 0.072 | 0.342 | 0.249 | -0.411 | 0.811 | 0.390 | 0.189 | -0.434 | 0.720 | 0.470 |
|  | 3 VS 2 | 265 | -0.086 | -0.251 | 0.050 | 0.198 | 0.363 | -0.294 | 0.970 | 0.259 | 0.277 | -0.353 | 0.832 | 0.345 |
| H5 Aggressive responding (subscale Approve) | 2 VS 1 | 262 | -0.026 | -0.092 | 0.011 | 0.206 | -0.051 | -0.406 | 0.299 | 0.782 | -0.076 | -0.433 | 0.267 | 0.667 |
|  | 3 VS 1 | 262 | -0.090 | -0.274 | 0.028 | 0.150 | 0.390 | -0.290 | 1.000 | 0.241 | 0.301 | -0.322 | 0.844 | 0.314 |
|  | 3 VS 2 | 262 | -0.064 | -0.227 | 0.027 | 0.202 | 0.441 | -0.240 | 1.066 | 0.196 | 0.377 | -0.264 | 0.968 | 0.235 |
| H6 Passive/submissive responding (subsacle Approve) | 2 VS 1 | 264 | 0.021 | -0.078 | 0.115 | 0.663 | -0.083 | -0.427 | 0.232 | 0.620 | -0.061 | -0.424 | 0.264 | 0.724 |
|  | 3 VS 1 | 264 | 0.169 | -0.014 | 0.394 | 0.067 | 0.125 | -0.475 | 0.634 | 0.627 | 0.294 | -0.338 | 0.842 | 0.317 |
|  | 3 VS 2 | 264 | 0.148 | -0.036 | 0.378 | 0.121 | 0.208 | -0.395 | 0.739 | 0.440 | 0.356 | -0.265 | 0.925 | 0.248 |
| H7 Negative other-beliefs (subscale BCSS) | 2 VS 1 | 261 | 0.010 | -0.015 | 0.052 | 0.522 | -0.060 | -0.399 | 0.283 | 0.764 | -0.050 | -0.388 | 0.288 | 0.808 |
|  | 3 VS 1 | 261 | 0.010 | -0.034 | 0.075 | 0.712 | 0.220 | -0.390 | 0.778 | 0.455 | 0.230 | -0.393 | 0.782 | 0.440 |
|  | 3 VS 2 | 261 | 0.000 | -0.052 | 0.056 | 0.997 | 0.280 | -0.370 | 0.862 | 0.372 | 0.280 | -0.381 | 0.861 | 0.376 |
| H8 Negative self-beliefs (subscale BCSS) | 2 VS 1 | 261 | 0.076 | -0.001 | 0.190 | 0.052 | -0.105 | -0.457 | 0.224 | 0.568 | -0.030 | -0.378 | 0.301 | 0.896 |
|  | 3 VS 1 | 261 | 0.128 | 0.000 | 0.315 | **0.048** | 0.124 | -0.493 | 0.656 | 0.650 | 0.252 | -0.369 | 0.815 | 0.397 |
|  | 3 VS 2 | 261 | 0.052 | -0.087 | 0.220 | 0.466 | 0.229 | -0.408 | 0.773 | 0.446 | 0.282 | -0.374 | 0.864 | 0.387 |
| H9 Cognitive reappraisal (subscale ERQ) | 2 VS 1 | 265 | 0.019 | -0.027 | 0.088 | 0.420 | -0.064 | -0.413 | 0.271 | 0.725 | -0.044 | -0.393 | 0.290 | 0.807 |
|  | 3 VS 1 | 265 | 0.007 | -0.086 | 0.122 | 0.859 | 0.230 | -0.386 | 0.759 | 0.424 | 0.236 | -0.392 | 0.791 | 0.425 |
|  | 3 VS 2 | 265 | -0.013 | -0.120 | 0.096 | 0.791 | 0.293 | -0.351 | 0.856 | 0.330 | 0.280 | -0.370 | 0.859 | 0.356 |
| H10 Expressive supression (subscale ERQ) | 2 VS 1 | 265 | 0.000 | -0.038 | 0.049 | 0.956 | -0.044 | -0.393 | 0.284 | 0.796 | -0.044 | -0.393 | 0.290 | 0.807 |
|  | 3 VS 1 | 265 | -0.015 | -0.098 | 0.059 | 0.723 | 0.251 | -0.392 | 0.810 | 0.405 | 0.236 | -0.392 | 0.791 | 0.425 |
|  | 3 VS 2 | 265 | -0.015 | -0.107 | 0.058 | 0.697 | 0.295 | -0.359 | 0.880 | 0.340 | 0.280 | -0.370 | 0.859 | 0.356 |
| H11 Depression (total CDSS) | 2 VS 1 | 260 | 0.050 | -0.052 | 0.163 | 0.329 | -0.102 | -0.446 | 0.228 | 0.583 | -0.052 | -0.412 | 0.288 | 0.802 |
|  | 3 VS 1 | 260 | 0.188 | -0.010 | 0.423 | 0.063 | 0.043 | -0.533 | 0.579 | 0.864 | 0.231 | -0.391 | 0.780 | 0.427 |
|  | 3 VS 2 | 260 | 0.138 | -0.067 | 0.379 | 0.190 | 0.144 | -0.454 | 0.699 | 0.627 | 0.282 | -0.380 | 0.867 | 0.378 |
| H12 Sleep disturbances (total PSQI) | 2 VS 1 | 261 | 0.116 | 0.032 | 0.230 | **0.002** | -0.129 | -0.471 | 0.212 | 0.472 | -0.014 | -0.346 | 0.334 | 0.952 |
|  | 3 VS 1 | 261 | 0.119 | -0.004 | 0.280 | 0.058 | 0.056 | -0.518 | 0.587 | 0.800 | 0.175 | -0.448 | 0.740 | 0.519 |
|  | 3 VS 2 | 261 | 0.004 | -0.136 | 0.134 | 0.952 | 0.185 | -0.384 | 0.717 | 0.482 | 0.189 | -0.444 | 0.780 | 0.512 |
| **Supplement 1.** Age-adjusted point estimates and uncertainty estimates for total, direct, and indirect effects. Abbreviations: H#: Hypothesis no.; Class 1: 'variable severity'; Class 2: 'severe neglect and emotional abuse'; Class 3: 'severe poly-trauma'. Indirect: Indirect effect; Direct: Direct effect; Total: Total effect; Lower/upper: lower and upper uncertainty estimates Measures: Approve: Approve-Voices; BAVQ-R: Beliefs About Voices Questionnaire - Revised; CTQ: Childhood Trauma Questionnaire; BCSS: Brief Core Schema Scale; CDSS: Calgary Depression Scale for Schizophrenia; ERQ: Emotion Regulation Questionnaire; PSQI; Pittsburgh Sleep Quality Index; VPDS: Voice Power Differential Scale. Significance level: p < .05 (highlighted). All CI set at 95%. | | | | | | | | | | | | | | |

| **Supplement 2.** Age-adjusted differences in indirect effect according to gender (m vs. f). | | | | | |
| --- | --- | --- | --- | --- | --- |
|  | Classes | Estimate | Lower | Upper | P |
| H1 Negative voice-content (items 6+7 PSYRATS-AHS) | 2 VS 1 | -0.431 | -0.955 | 0.021 | 0.064 |
|  | 3 VS 1 | -0.386 | -1.096 | 0.193 | 0.210 |
|  | 3 VS 2 | 0.046 | -0.665 | 0.721 | 0.881 |
| H2 Persecutory belief factor (subscale BAVQ-R) | 2 VS 1 | 0.193 | -0.112 | 0.541 | 0.241 |
|  | 3 VS 1 | -0.133 | -0.760 | 0.432 | 0.617 |
|  | 3 VS 2 | -0.326 | -0.997 | 0.265 | 0.272 |
| H3 Voice power differential (total VPDS) | 2 VS 1 | -0.150 | -0.382 | 0.050 | 0.143 |
|  | 3 VS 1 | 0.113 | -0.224 | 0.469 | 0.479 |
|  | 3 VS 2 | 0.263 | -0.067 | 0.639 | 0.111 |
| H4 Assertive relating (subscale Approve) | 2 VS 1 | 0.068 | -0.069 | 0.213 | 0.330 |
|  | 3 VS 1 | -0.088 | -0.413 | 0.166 | 0.477 |
|  | 3 VS 2 | -0.156 | -0.495 | 0.098 | 0.225 |
| H5 Aggressive responding (subscale Approve) | 2 VS 1 | 0.049 | -0.057 | 0.257 | 0.470 |
|  | 3 VS 1 | 0.144 | -0.200 | 0.608 | 0.425 |
|  | 3 VS 2 | 0.095 | -0.192 | 0.462 | 0.552 |
| H6 Passive/submissive responding (subsacle Approve) | 2 VS 1 | 0.054 | -0.125 | 0.248 | 0.554 |
|  | 3 VS 1 | 0.105 | -0.338 | 0.516 | 0.550 |
|  | 3 VS 2 | 0.051 | -0.424 | 0.461 | 0.745 |
| H7 Negative other-beliefs (subscale BCSS) | 2 VS 1 | 0.025 | -0.049 | 0.124 | 0.491 |
|  | 3 VS 1 | 0.062 | -0.113 | 0.253 | 0.502 |
|  | 3 VS 2 | 0.037 | -0.133 | 0.216 | 0.688 |
| H8 Negative self-beliefs (subscale BCSS) | 2 VS 1 | 0.014 | -0.196 | 0.200 | 0.810 |
|  | 3 VS 1 | 0.032 | -0.287 | 0.341 | 0.767 |
|  | 3 VS 2 | 0.017 | -0.299 | 0.334 | 0.876 |
| H9 Cognitive reappraisal (subscale ERQ) | 2 VS 1 | 0.010 | -0.121 | 0.132 | 0.846 |
|  | 3 VS 1 | 0.146 | -0.082 | 0.455 | 0.223 |
|  | 3 VS 2 | 0.136 | -0.124 | 0.481 | 0.311 |
| H10 Expressive supression (subscale ERQ) | 2 VS 1 | 0.040 | -0.089 | 0.187 | 0.551 |
|  | 3 VS 1 | -0.047 | -0.273 | 0.158 | 0.597 |
|  | 3 VS 2 | -0.087 | -0.353 | 0.145 | 0.428 |
| H11 Depression (total CDSS) | 2 VS 1 | -0.049 | -0.298 | 0.160 | 0.637 |
|  | 3 VS 1 | 0.156 | -0.233 | 0.570 | 0.432 |
|  | 3 VS 2 | 0.205 | -0.206 | 0.635 | 0.302 |
| H12 Sleep disturbances (total PSQI) | 2 VS 1 | 0.027 | -0.223 | 0.237 | 0.732 |
|  | 3 VS 1 | 0.130 | -0.156 | 0.418 | 0.326 |
|  | 3 VS 2 | 0.103 | -0.175 | 0.414 | 0.460 |
| **Supplement 2**. Age-adjusted point estimates and uncertainty estimates for differences in indirect effect according to gender (female VS male). Abbreviations: H#: Hypothesis no.; Class 1: 'variable severity'; Class 2: 'severe neglect and emotional abuse'; Class 3: 'severe poly-trauma'. Estimate: Estimate of difference in indrect effects comparing female and male participants; Lower/upper: lower and upper uncertainty estimates Measures: Approve: Approve-Voices; BAVQ-R: Beliefs About Voices Questionnaire - Revised; CTQ: Childhood Trauma Questionnaire; BCSS: Brief Core Schema Scale; CDSS: Calgary Depression Scale for Schizophrenia; ERQ: Emotion Regulation Questionnaire; PSQI; Pittsburgh Sleep Quality Index; VPDS: Voice Power Differential Scale. Significance level: p < .05 (highlighted). All CI set at 95%. | | | | | |

| **Supplement 3.** Point estimates and uncertainty estimates for total, direct, and indirect effects after exclusion of individuals with CTQ validity score >0 on the minimization and denial scale. | | | | | | | | | | | | | | | |
| --- | --- | --- | --- | --- | --- | --- | --- | --- | --- | --- | --- | --- | --- | --- | --- |
|  | Class | N | Indirect | Lower | Upper | P | Direct | Lower | Upper | P | Total | Lower | Upper | P | bh_significant |
| H1 Negative voice-content (items 6+7 PSYRATS-AHS) | 2 VS 1 | 190 | -0.0592 | -0.3496 | 0.2298 | 0.6988 | 0.0806 | -0.2324 | 0.3934 | 0.6164 | 0.0214 | -0.3739 | 0.4202 | 0.9076 | 0 |
|  | 3 VS 1 | 190 | 0.1022 | -0.2766 | 0.4650 | 0.5604 | 0.1575 | -0.3338 | 0.6491 | 0.5096 | 0.2597 | -0.4483 | 0.9062 | 0.4228 | 0 |
|  | 3 VS 2 | 190 | 0.1615 | -0.2368 | 0.5401 | 0.4068 | 0.0769 | -0.4155 | 0.5733 | 0.7428 | 0.2383 | -0.4692 | 0.8873 | 0.4696 | 0 |
| H2 Persecutory belief factor (subscale BAVQ-R) | 2 VS 1 | 189 | 0.0376 | -0.1414 | 0.2296 | 0.6904 | -0.0163 | -0.4109 | 0.3500 | 0.9208 | 0.0214 | -0.4039 | 0.4344 | 0.9252 | 0 |
|  | 3 VS 1 | 189 | 0.2670 | -0.0275 | 0.5866 | 0.0812 | -0.0837 | -0.7473 | 0.4866 | 0.8220 | 0.1834 | -0.5455 | 0.8160 | 0.5716 | 0 |
|  | 3 VS 2 | 189 | 0.2294 | -0.0832 | 0.5449 | 0.1444 | -0.0674 | -0.7198 | 0.5127 | 0.8608 | 0.1620 | -0.5593 | 0.8105 | 0.6260 | 0 |
| H3 Voice power differential (total VPDS) | 2 VS 1 | 188 | 0.0834 | -0.0666 | 0.2493 | 0.2588 | -0.0239 | -0.4235 | 0.3602 | 0.9064 | 0.0595 | -0.3658 | 0.4669 | 0.7812 | 0 |
|  | 3 VS 1 | 188 | 0.2577 | 0.0235 | 0.5319 | **0.0352** | 0.0097 | -0.6515 | 0.5914 | 0.9396 | 0.2674 | -0.4667 | 0.9085 | 0.4296 | 0 |
|  | 3 VS 2 | 188 | 0.1743 | -0.0454 | 0.4323 | 0.1328 | 0.0336 | -0.6202 | 0.5938 | 0.8652 | 0.2079 | -0.5182 | 0.8423 | 0.5188 | 0 |
| H4 Assertive relating (subscale Approve) | 2 VS 1 | 189 | 0.0235 | -0.0718 | 0.1301 | 0.6280 | -0.0705 | -0.4554 | 0.3079 | 0.7140 | -0.0470 | -0.4551 | 0.3522 | 0.8088 | 0 |
|  | 3 VS 1 | 189 | -0.1102 | -0.3227 | 0.0501 | 0.1748 | 0.3015 | -0.4635 | 0.9516 | 0.3800 | 0.1913 | -0.5191 | 0.8024 | 0.5316 | 0 |
|  | 3 VS 2 | 189 | -0.1337 | -0.3677 | 0.0306 | 0.1180 | 0.3720 | -0.4113 | 1.0720 | 0.3104 | 0.2383 | -0.4732 | 0.8571 | 0.4564 | 0 |
| H5 Aggressive responding (subscale Approve) | 2 VS 1 | 187 | -0.0413 | -0.1349 | 0.0175 | 0.1992 | 0.0155 | -0.4033 | 0.4229 | 0.9524 | -0.0257 | -0.4420 | 0.3865 | 0.8892 | 0 |
|  | 3 VS 1 | 187 | -0.1645 | -0.4682 | 0.0288 | 0.1020 | 0.4808 | -0.3689 | 1.2424 | 0.2412 | 0.3164 | -0.4288 | 0.9513 | 0.3468 | 0 |
|  | 3 VS 2 | 187 | -0.1232 | -0.3909 | 0.0254 | 0.1264 | 0.4653 | -0.3512 | 1.2160 | 0.2364 | 0.3421 | -0.3848 | 0.9861 | 0.3160 | 0 |
| H6 Passive/submissive responding (subsacle Approve) | 2 VS 1 | 188 | 0.0307 | -0.0947 | 0.1586 | 0.5852 | -0.0342 | -0.4511 | 0.3529 | 0.8396 | -0.0035 | -0.4371 | 0.3951 | 0.9660 | 0 |
|  | 3 VS 1 | 188 | 0.1778 | -0.0332 | 0.4374 | 0.1072 | 0.1386 | -0.5857 | 0.7249 | 0.6368 | 0.3164 | -0.4392 | 0.9452 | 0.3480 | 0 |
|  | 3 VS 2 | 188 | 0.1471 | -0.0661 | 0.4083 | 0.1772 | 0.1728 | -0.5565 | 0.7884 | 0.5632 | 0.3199 | -0.4256 | 0.9597 | 0.3404 | 0 |
| H7 Negative other-beliefs (subscale BCSS) | 2 VS 1 | 187 | 0.0048 | -0.0306 | 0.0497 | 0.7840 | 0.0069 | -0.4092 | 0.4344 | 0.9972 | 0.0117 | -0.3981 | 0.4367 | 0.9724 | 0 |
|  | 3 VS 1 | 187 | 0.0139 | -0.0435 | 0.1077 | 0.6944 | 0.2361 | -0.4708 | 0.8631 | 0.4896 | 0.2500 | -0.4636 | 0.8802 | 0.4684 | 0 |
|  | 3 VS 2 | 187 | 0.0091 | -0.0517 | 0.0987 | 0.7996 | 0.2292 | -0.4944 | 0.8747 | 0.4880 | 0.2383 | -0.5058 | 0.8877 | 0.4784 | 0 |
| H8 Negative self-beliefs (subscale BCSS) | 2 VS 1 | 187 | 0.0674 | -0.0173 | 0.2121 | 0.1396 | -0.0224 | -0.4355 | 0.3837 | 0.9028 | 0.0450 | -0.3709 | 0.4601 | 0.8348 | 0 |
|  | 3 VS 1 | 187 | 0.0996 | -0.0245 | 0.3040 | 0.1440 | 0.1837 | -0.5883 | 0.7866 | 0.5628 | 0.2833 | -0.4900 | 0.9107 | 0.4132 | 0 |
|  | 3 VS 2 | 187 | 0.0322 | -0.1065 | 0.2022 | 0.6792 | 0.2061 | -0.5226 | 0.7960 | 0.5156 | 0.2383 | -0.5020 | 0.8549 | 0.4792 | 0 |
| H9 Cognitive reappraisal (subscale ERQ) | 2 VS 1 | 189 | 0.0013 | -0.0749 | 0.0819 | 0.9828 | 0.0277 | -0.3932 | 0.4339 | 0.8948 | 0.0291 | -0.3915 | 0.4367 | 0.8900 | 0 |
|  | 3 VS 1 | 189 | -0.0200 | -0.1499 | 0.1061 | 0.7272 | 0.2874 | -0.3973 | 0.9001 | 0.3756 | 0.2674 | -0.4280 | 0.8965 | 0.4216 | 0 |
|  | 3 VS 2 | 189 | -0.0214 | -0.1595 | 0.1115 | 0.7328 | 0.2597 | -0.4272 | 0.8778 | 0.4328 | 0.2383 | -0.4685 | 0.8840 | 0.4784 | 0 |
| H10 Expressive supression (subscale ERQ) | 2 VS 1 | 189 | -0.0067 | -0.0729 | 0.0729 | 0.8800 | 0.0358 | -0.3845 | 0.4281 | 0.8676 | 0.0291 | -0.3915 | 0.4367 | 0.8900 | 0 |
|  | 3 VS 1 | 189 | -0.0315 | -0.1564 | 0.0813 | 0.5748 | 0.2989 | -0.4149 | 0.9384 | 0.3904 | 0.2674 | -0.4280 | 0.8965 | 0.4216 | 0 |
|  | 3 VS 2 | 189 | -0.0248 | -0.1590 | 0.0880 | 0.6616 | 0.2631 | -0.4586 | 0.9124 | 0.4388 | 0.2383 | -0.4685 | 0.8840 | 0.4784 | 0 |
| H11 Depression (total CDSS) | 2 VS 1 | 185 | 0.0071 | -0.1178 | 0.1260 | 0.9228 | 0.0106 | -0.3933 | 0.4132 | 0.9716 | 0.0178 | -0.4034 | 0.4404 | 0.9504 | 0 |
|  | 3 VS 1 | 185 | 0.1289 | -0.0743 | 0.3763 | 0.2160 | 0.1322 | -0.5536 | 0.7494 | 0.6556 | 0.2611 | -0.4626 | 0.8908 | 0.4412 | 0 |
|  | 3 VS 2 | 185 | 0.1217 | -0.0845 | 0.3800 | 0.2524 | 0.1216 | -0.5602 | 0.7292 | 0.6764 | 0.2433 | -0.4754 | 0.8825 | 0.4596 | 0 |
| H12 Sleep disturbances (total PSQI) | 2 VS 1 | 187 | 0.1019 | 0.0089 | 0.2379 | **0.0276** | -0.0424 | -0.4601 | 0.3737 | 0.8636 | 0.0595 | -0.3512 | 0.4743 | 0.7544 | 0 |
|  | 3 VS 1 | 187 | 0.1182 | -0.0304 | 0.3037 | 0.1312 | 0.0729 | -0.6174 | 0.6725 | 0.7976 | 0.1911 | -0.5730 | 0.8235 | 0.5784 | 0 |
|  | 3 VS 2 | 187 | 0.0163 | -0.1479 | 0.1640 | 0.8504 | 0.1153 | -0.5550 | 0.6915 | 0.6988 | 0.1316 | -0.6140 | 0.7539 | 0.6976 | 0 |
| **Supplement 3.** Point estimates and uncertainty estimates for total, direct, and indirect effects after exclusion of individuals with CTQ validity score >0 on the minimization and denial scale. Abbreviations: H#: Hypothesis no.; Class 1: 'variable severity'; Class 2: 'severe neglect and emotional abuse'; Class 3: 'severe poly-trauma'. Indirect: Indirect effect; Direct: Direct effect; Total: Total effect; Lower/upper: lower and upper uncertainty estimates; bh_significant: Benjamini-Hochberg correction (0=non-significant after correction; 1= significant after correction). Measures: Approve: Approve-Voices; BAVQ-R: Beliefs About Voices Questionnaire - Revised; CTQ: Childhood Trauma Questionnaire; BCSS: Brief Core Schema Scale; CDSS: Calgary Depression Scale for Schizophrenia; ERQ: Emotion Regulation Questionnaire; PSQI; Pittsburgh Sleep Quality Index; VPDS: Voice Power Differential Scale. Significance level: p < .05 (highlighted). All CI set at 95%. | | | | | | | | | | | | | | | |

| **Supplement 3.** Point estimates and uncertainty estimates for total, direct, and indirect effects after exclusion of individuals with CTQ validity score >0 on the minimization and denial scale. | | | | | | | | | | | | | | | |
| --- | --- | --- | --- | --- | --- | --- | --- | --- | --- | --- | --- | --- | --- | --- | --- |
|  | Class | N | Indirect | Lower | Upper | P | Direct | Lower | Upper | P | Total | Lower | Upper | P | bh_significant |
| H1 Negative voice-content (items 6+7 PSYRATS-AHS) | 2 VS 1 | 190 | -0.0592 | -0.3496 | 0.2298 | 0.6988 | 0.0806 | -0.2324 | 0.3934 | 0.6164 | 0.0214 | -0.3739 | 0.4202 | 0.9076 | 0 |
|  | 3 VS 1 | 190 | 0.1022 | -0.2766 | 0.4650 | 0.5604 | 0.1575 | -0.3338 | 0.6491 | 0.5096 | 0.2597 | -0.4483 | 0.9062 | 0.4228 | 0 |
|  | 3 VS 2 | 190 | 0.1615 | -0.2368 | 0.5401 | 0.4068 | 0.0769 | -0.4155 | 0.5733 | 0.7428 | 0.2383 | -0.4692 | 0.8873 | 0.4696 | 0 |
| H2 Persecutory belief factor (subscale BAVQ-R) | 2 VS 1 | 189 | 0.0376 | -0.1414 | 0.2296 | 0.6904 | -0.0163 | -0.4109 | 0.3500 | 0.9208 | 0.0214 | -0.4039 | 0.4344 | 0.9252 | 0 |
|  | 3 VS 1 | 189 | 0.2670 | -0.0275 | 0.5866 | 0.0812 | -0.0837 | -0.7473 | 0.4866 | 0.8220 | 0.1834 | -0.5455 | 0.8160 | 0.5716 | 0 |
|  | 3 VS 2 | 189 | 0.2294 | -0.0832 | 0.5449 | 0.1444 | -0.0674 | -0.7198 | 0.5127 | 0.8608 | 0.1620 | -0.5593 | 0.8105 | 0.6260 | 0 |
| H3 Voice power differential (total VPDS) | 2 VS 1 | 188 | 0.0834 | -0.0666 | 0.2493 | 0.2588 | -0.0239 | -0.4235 | 0.3602 | 0.9064 | 0.0595 | -0.3658 | 0.4669 | 0.7812 | 0 |
|  | 3 VS 1 | 188 | 0.2577 | 0.0235 | 0.5319 | **0.0352** | 0.0097 | -0.6515 | 0.5914 | 0.9396 | 0.2674 | -0.4667 | 0.9085 | 0.4296 | 0 |
|  | 3 VS 2 | 188 | 0.1743 | -0.0454 | 0.4323 | 0.1328 | 0.0336 | -0.6202 | 0.5938 | 0.8652 | 0.2079 | -0.5182 | 0.8423 | 0.5188 | 0 |
| H4 Assertive relating (subscale Approve) | 2 VS 1 | 189 | 0.0235 | -0.0718 | 0.1301 | 0.6280 | -0.0705 | -0.4554 | 0.3079 | 0.7140 | -0.0470 | -0.4551 | 0.3522 | 0.8088 | 0 |
|  | 3 VS 1 | 189 | -0.1102 | -0.3227 | 0.0501 | 0.1748 | 0.3015 | -0.4635 | 0.9516 | 0.3800 | 0.1913 | -0.5191 | 0.8024 | 0.5316 | 0 |
|  | 3 VS 2 | 189 | -0.1337 | -0.3677 | 0.0306 | 0.1180 | 0.3720 | -0.4113 | 1.0720 | 0.3104 | 0.2383 | -0.4732 | 0.8571 | 0.4564 | 0 |
| H5 Aggressive responding (subscale Approve) | 2 VS 1 | 187 | -0.0413 | -0.1349 | 0.0175 | 0.1992 | 0.0155 | -0.4033 | 0.4229 | 0.9524 | -0.0257 | -0.4420 | 0.3865 | 0.8892 | 0 |
|  | 3 VS 1 | 187 | -0.1645 | -0.4682 | 0.0288 | 0.1020 | 0.4808 | -0.3689 | 1.2424 | 0.2412 | 0.3164 | -0.4288 | 0.9513 | 0.3468 | 0 |
|  | 3 VS 2 | 187 | -0.1232 | -0.3909 | 0.0254 | 0.1264 | 0.4653 | -0.3512 | 1.2160 | 0.2364 | 0.3421 | -0.3848 | 0.9861 | 0.3160 | 0 |
| H6 Passive/submissive responding (subsacle Approve) | 2 VS 1 | 188 | 0.0307 | -0.0947 | 0.1586 | 0.5852 | -0.0342 | -0.4511 | 0.3529 | 0.8396 | -0.0035 | -0.4371 | 0.3951 | 0.9660 | 0 |
|  | 3 VS 1 | 188 | 0.1778 | -0.0332 | 0.4374 | 0.1072 | 0.1386 | -0.5857 | 0.7249 | 0.6368 | 0.3164 | -0.4392 | 0.9452 | 0.3480 | 0 |
|  | 3 VS 2 | 188 | 0.1471 | -0.0661 | 0.4083 | 0.1772 | 0.1728 | -0.5565 | 0.7884 | 0.5632 | 0.3199 | -0.4256 | 0.9597 | 0.3404 | 0 |
| H7 Negative other-beliefs (subscale BCSS) | 2 VS 1 | 187 | 0.0048 | -0.0306 | 0.0497 | 0.7840 | 0.0069 | -0.4092 | 0.4344 | 0.9972 | 0.0117 | -0.3981 | 0.4367 | 0.9724 | 0 |
|  | 3 VS 1 | 187 | 0.0139 | -0.0435 | 0.1077 | 0.6944 | 0.2361 | -0.4708 | 0.8631 | 0.4896 | 0.2500 | -0.4636 | 0.8802 | 0.4684 | 0 |
|  | 3 VS 2 | 187 | 0.0091 | -0.0517 | 0.0987 | 0.7996 | 0.2292 | -0.4944 | 0.8747 | 0.4880 | 0.2383 | -0.5058 | 0.8877 | 0.4784 | 0 |
| H8 Negative self-beliefs (subscale BCSS) | 2 VS 1 | 187 | 0.0674 | -0.0173 | 0.2121 | 0.1396 | -0.0224 | -0.4355 | 0.3837 | 0.9028 | 0.0450 | -0.3709 | 0.4601 | 0.8348 | 0 |
|  | 3 VS 1 | 187 | 0.0996 | -0.0245 | 0.3040 | 0.1440 | 0.1837 | -0.5883 | 0.7866 | 0.5628 | 0.2833 | -0.4900 | 0.9107 | 0.4132 | 0 |
|  | 3 VS 2 | 187 | 0.0322 | -0.1065 | 0.2022 | 0.6792 | 0.2061 | -0.5226 | 0.7960 | 0.5156 | 0.2383 | -0.5020 | 0.8549 | 0.4792 | 0 |
| H9 Cognitive reappraisal (subscale ERQ) | 2 VS 1 | 189 | 0.0013 | -0.0749 | 0.0819 | 0.9828 | 0.0277 | -0.3932 | 0.4339 | 0.8948 | 0.0291 | -0.3915 | 0.4367 | 0.8900 | 0 |
|  | 3 VS 1 | 189 | -0.0200 | -0.1499 | 0.1061 | 0.7272 | 0.2874 | -0.3973 | 0.9001 | 0.3756 | 0.2674 | -0.4280 | 0.8965 | 0.4216 | 0 |
|  | 3 VS 2 | 189 | -0.0214 | -0.1595 | 0.1115 | 0.7328 | 0.2597 | -0.4272 | 0.8778 | 0.4328 | 0.2383 | -0.4685 | 0.8840 | 0.4784 | 0 |
| H10 Expressive supression (subscale ERQ) | 2 VS 1 | 189 | -0.0067 | -0.0729 | 0.0729 | 0.8800 | 0.0358 | -0.3845 | 0.4281 | 0.8676 | 0.0291 | -0.3915 | 0.4367 | 0.8900 | 0 |
|  | 3 VS 1 | 189 | -0.0315 | -0.1564 | 0.0813 | 0.5748 | 0.2989 | -0.4149 | 0.9384 | 0.3904 | 0.2674 | -0.4280 | 0.8965 | 0.4216 | 0 |
|  | 3 VS 2 | 189 | -0.0248 | -0.1590 | 0.0880 | 0.6616 | 0.2631 | -0.4586 | 0.9124 | 0.4388 | 0.2383 | -0.4685 | 0.8840 | 0.4784 | 0 |
| H11 Depression (total CDSS) | 2 VS 1 | 185 | 0.0071 | -0.1178 | 0.1260 | 0.9228 | 0.0106 | -0.3933 | 0.4132 | 0.9716 | 0.0178 | -0.4034 | 0.4404 | 0.9504 | 0 |
|  | 3 VS 1 | 185 | 0.1289 | -0.0743 | 0.3763 | 0.2160 | 0.1322 | -0.5536 | 0.7494 | 0.6556 | 0.2611 | -0.4626 | 0.8908 | 0.4412 | 0 |
|  | 3 VS 2 | 185 | 0.1217 | -0.0845 | 0.3800 | 0.2524 | 0.1216 | -0.5602 | 0.7292 | 0.6764 | 0.2433 | -0.4754 | 0.8825 | 0.4596 | 0 |
| H12 Sleep disturbances (total PSQI) | 2 VS 1 | 187 | 0.1019 | 0.0089 | 0.2379 | **0.0276** | -0.0424 | -0.4601 | 0.3737 | 0.8636 | 0.0595 | -0.3512 | 0.4743 | 0.7544 | 0 |
|  | 3 VS 1 | 187 | 0.1182 | -0.0304 | 0.3037 | 0.1312 | 0.0729 | -0.6174 | 0.6725 | 0.7976 | 0.1911 | -0.5730 | 0.8235 | 0.5784 | 0 |
|  | 3 VS 2 | 187 | 0.0163 | -0.1479 | 0.1640 | 0.8504 | 0.1153 | -0.5550 | 0.6915 | 0.6988 | 0.1316 | -0.6140 | 0.7539 | 0.6976 | 0 |
| **Supplement 3.** Point estimates and uncertainty estimates for total, direct, and indirect effects after exclusion of individuals with CTQ validity score >0 on the minimization and denial scale. Abbreviations: H#: Hypothesis no.; Class 1: 'variable severity'; Class 2: 'severe neglect and emotional abuse'; Class 3: 'severe poly-trauma'. Indirect: Indirect effect; Direct: Direct effect; Total: Total effect; Lower/upper: lower and upper uncertainty estimates; bh_significant: Benjamini-Hochberg correction (0=non-significant after correction; 1= significant after correction). Measures: Approve: Approve-Voices; BAVQ-R: Beliefs About Voices Questionnaire - Revised; CTQ: Childhood Trauma Questionnaire; BCSS: Brief Core Schema Scale; CDSS: Calgary Depression Scale for Schizophrenia; ERQ: Emotion Regulation Questionnaire; PSQI; Pittsburgh Sleep Quality Index; VPDS: Voice Power Differential Scale. Significance level: p < .05 (highlighted). All CI set at 95%. | | | | | | | | | | | | | | | |

| **Supplement 4**. Point estimates and uncertainty estimates for total, direct, and indirect effects treating trauma exposure as continuous CTQ score. | | | | | | | | | | | | | | |
| --- | --- | --- | --- | --- | --- | --- | --- | --- | --- | --- | --- | --- | --- | --- |
|  | N | Indirect | Lower | Upper | P | Direct | Lower | Upper | P | Total | Lower | Upper | P | BH significant |
| H1 Negative voice-content (items 6+7 PSYRATS-AHS) | 266 | 0.0022 | -0.0026 | 0.0066 | 0.3408 | 0.0018 | -0.0035 | 0.0071 | 0.4856 | 0.0040 | -0.0034 | 0.0109 | 0.2668 | 0 |
| H2 Persecutory belief factor (subscale BAVQ-R) | 265 | 0.0030 | -0.0006 | 0.0069 | 0.0996 | 0.0002 | -0.0065 | 0.0063 | 0.9716 | 0.0032 | -0.0040 | 0.0101 | 0.3912 | 0 |
| H3 Voice power differential (total VPDS) | 263 | 0.0022 | -0.0002 | 0.0049 | 0.0768 | 0.0023 | -0.0045 | 0.0086 | 0.4672 | 0.0045 | -0.0026 | 0.0111 | 0.1972 | 0 |
| H4 Assertive relating (subscale Approve) | 265 | -0.0001 | -0.0016 | 0.0014 | 0.8484 | 0.0036 | -0.0035 | 0.0102 | 0.3108 | 0.0035 | -0.0038 | 0.0101 | 0.3312 | 0 |
| H5 Aggressive responding (subscale Approve) | 262 | -0.0008 | -0.0025 | 0.0003 | 0.1708 | 0.0047 | -0.0028 | 0.0117 | 0.2068 | 0.0039 | -0.0035 | 0.0107 | 0.2904 | 0 |
| H6 Passive/submissive responding (subsacle Approve) | 264 | 0.0007 | -0.0016 | 0.0032 | 0.5360 | 0.0034 | -0.0036 | 0.0096 | 0.3192 | 0.0042 | -0.0032 | 0.0106 | 0.2492 | 0 |
| H7 Negative other-beliefs (subscale BCSS) | 261 | 0.0002 | -0.0003 | 0.0013 | 0.5104 | 0.0039 | -0.0033 | 0.0107 | 0.2812 | 0.0041 | -0.0030 | 0.0110 | 0.2540 | 0 |
| H8 Negative self-beliefs (subscale BCSS) | 261 | 0.0018 | 0.0002 | 0.0043 | **0.0272** | 0.0027 | -0.0046 | 0.0097 | 0.4616 | 0.0045 | -0.0026 | 0.0115 | 0.2152 | 0 |
| H9 Cognitive reappraisal (subscale ERQ) | 265 | 0.0005 | -0.0004 | 0.0021 | 0.3052 | 0.0035 | -0.0036 | 0.0103 | 0.3332 | 0.0041 | -0.0031 | 0.0108 | 0.2700 | 0 |
| H10 Expressive supression (subscale ERQ) | 265 | 0.0000 | -0.0008 | 0.0011 | 0.9120 | 0.0040 | -0.0031 | 0.0107 | 0.2840 | 0.0041 | -0.0031 | 0.0108 | 0.2700 | 0 |
| H11 Depression (total CDSS) | 260 | 0.0031 | 0.0007 | 0.0060 | **0.0080** | 0.0009 | -0.0063 | 0.0077 | 0.7800 | 0.0040 | -0.0033 | 0.0109 | 0.2880 | **1** |
| H12 Sleep disturbances (total PSQI) | 261 | 0.0035 | 0.0012 | 0.0064 | **0.0008** | -0.0000 | -0.0076 | 0.0068 | 0.9768 | 0.0035 | -0.0039 | 0.0102 | 0.3360 | **1** |
| **Supplement 4.** Point estimates and uncertainty estimates for total, direct, and indirect effects treating trauma exposure as continuous CTQ score. Abbreviations: H#: Hypothesis no.; Indirect: Indirect effect; Direct: Direct effect; Total: Total effect; Lower/upper: lower and upper uncertainty estimates; bh_significant: Benjamini-Hochberg correction (0=non-significant after correction; 1= significant after correction). Measures: Approve: Approve-Voices; BAVQ-R: Beliefs About Voices Questionnaire - Revised; CTQ: Childhood Trauma Questionnaire; BCSS: Brief Core Schema Scale; CDSS: Calgary Depression Scale for Schizophrenia; ERQ: Emotion Regulation Questionnaire; PSQI; Pittsburgh Sleep Quality Index; VPDS: Voice Power Differential Scale. Significance level: p < .05 (highlighted). All CI set at 95%. | | | | | | | | | | | | | | |

| **Supplement 6.** Point estimates and uncertainty estimates for total, direct, and indirect effects operationalizing outcome as sum score of PSYRATS-AH items 6,7,8,9,11. | | | | | | | | | | | | | | | |
| --- | --- | --- | --- | --- | --- | --- | --- | --- | --- | --- | --- | --- | --- | --- | --- |
|  | Class | N | Indirect | Lower | Upper | P | Direct | Lower | Upper | P | Total | Lower | Upper | P | bh_significant |
| H1 | n/a | n/a | n/a | n/a | n/a | n/a | n/a | n/a | n/a | n/a | n/a | n/a | n/a | n/a | n/a |
| H2 Persecutory belief factor (subscale BAVQ-R) | 2 VS 1 | 265 | 0.1001 | -0.2688 | 0.4924 | 0.6152 | -0.2534 | -0.9414 | 0.3639 | 0.4372 | -0.1533 | -0.9162 | 0.5555 | 0.6892 | 0 |
|  | 3 VS 1 | 265 | 0.6507 | 0.0078 | 1.2908 | **0.0484** | -0.3515 | -1.3631 | 0.6120 | 0.4936 | 0.2991 | -0.9948 | 1.4496 | 0.6072 | 0 |
|  | 3 VS 2 | 265 | 0.5505 | -0.0989 | 1.2029 | 0.0928 | -0.0981 | -1.1935 | 0.9583 | 0.8660 | 0.4524 | -0.9165 | 1.7019 | 0.4696 | 0 |
| H3 Voice power differential (total VPDS) | 2 VS 1 | 263 | 0.1717 | -0.1115 | 0.4694 | 0.2328 | -0.2842 | -0.9709 | 0.3497 | 0.4004 | -0.1126 | -0.8464 | 0.5692 | 0.7640 | 0 |
|  | 3 VS 1 | 263 | 0.4611 | 0.0182 | 0.9623 | **0.0408** | -0.0692 | -1.1754 | 0.8766 | 0.9548 | 0.3918 | -0.8418 | 1.4736 | 0.4732 | 0 |
|  | 3 VS 2 | 263 | 0.2894 | -0.1523 | 0.7907 | 0.1944 | 0.2150 | -0.9887 | 1.2788 | 0.6712 | 0.5044 | -0.8310 | 1.6742 | 0.4072 | 0 |
| H4 Assertive relating (subscale Approve) | 2 VS 1 | 265 | 0.0587 | -0.1215 | 0.2573 | 0.5160 | -0.2990 | -0.9946 | 0.3673 | 0.3952 | -0.2403 | -0.9585 | 0.4383 | 0.5200 | 0 |
|  | 3 VS 1 | 265 | -0.1822 | -0.5576 | 0.1565 | 0.2676 | 0.4786 | -0.8619 | 1.6771 | 0.4280 | 0.2965 | -0.9569 | 1.3981 | 0.5672 | 0 |
|  | 3 VS 2 | 265 | -0.2409 | -0.6411 | 0.1089 | 0.1692 | 0.7777 | -0.6418 | 2.0769 | 0.2512 | 0.5368 | -0.7982 | 1.7546 | 0.3900 | 0 |
| H5 Aggressive responding (subscale Approve) | 2 VS 1 | 262 | -0.0719 | -0.2113 | 0.0129 | 0.1076 | -0.1636 | -0.9438 | 0.5428 | 0.6832 | -0.2355 | -1.0393 | 0.4704 | 0.5504 | 0 |
|  | 3 VS 1 | 262 | -0.2475 | -0.6278 | -0.0019 | **0.0476** | 0.6471 | -0.7178 | 1.8997 | 0.3244 | 0.3996 | -0.8759 | 1.5391 | 0.4908 | 0 |
|  | 3 VS 2 | 262 | -0.1756 | -0.5283 | 0.0269 | 0.1048 | 0.8107 | -0.6373 | 2.1343 | 0.2444 | 0.6351 | -0.7313 | 1.8720 | 0.3272 | 0 |
| H6 Passive/submissive responding (subsacle Approve) | 2 VS 1 | 264 | 0.0408 | -0.1785 | 0.2602 | 0.7200 | -0.2497 | -0.9792 | 0.4155 | 0.4620 | -0.2089 | -0.9712 | 0.4826 | 0.5464 | 0 |
|  | 3 VS 1 | 264 | 0.3695 | -0.0444 | 0.8767 | 0.0824 | 0.0169 | -1.2019 | 1.0768 | 0.9440 | 0.3863 | -0.9350 | 1.5211 | 0.5184 | 0 |
|  | 3 VS 2 | 264 | 0.3287 | -0.0891 | 0.8293 | 0.1292 | 0.2666 | -1.0149 | 1.4189 | 0.6192 | 0.5952 | -0.7553 | 1.8466 | 0.3496 | 0 |
| H7 Negative other-beliefs (subscale BCSS) | 2 VS 1 | 261 | 0.0256 | -0.0277 | 0.1199 | 0.4164 | -0.1803 | -0.9220 | 0.5204 | 0.6628 | -0.1547 | -0.9014 | 0.5537 | 0.7156 | 0 |
|  | 3 VS 1 | 261 | 0.0242 | -0.0884 | 0.1738 | 0.7048 | 0.3579 | -0.8475 | 1.4749 | 0.5296 | 0.3821 | -0.8414 | 1.4929 | 0.5048 | 0 |
|  | 3 VS 2 | 261 | -0.0014 | -0.1361 | 0.1332 | 0.9584 | 0.5382 | -0.7561 | 1.7259 | 0.4000 | 0.5368 | -0.7677 | 1.7380 | 0.4040 | 0 |
| H8 Negative self-beliefs (subscale BCSS) | 2 VS 1 | 261 | 0.1735 | -0.0169 | 0.4356 | 0.0824 | -0.2895 | -1.0385 | 0.3804 | 0.4392 | -0.1160 | -0.8646 | 0.5998 | 0.7928 | 0 |
|  | 3 VS 1 | 261 | 0.2775 | -0.0116 | 0.6698 | 0.0640 | 0.1433 | -1.0996 | 1.2020 | 0.7760 | 0.4208 | -0.8221 | 1.5326 | 0.4524 | 0 |
|  | 3 VS 2 | 261 | 0.1040 | -0.2224 | 0.4721 | 0.5360 | 0.4328 | -0.8736 | 1.5736 | 0.4728 | 0.5368 | -0.8069 | 1.7181 | 0.4020 | 0 |
| H9 Cognitive reappraisal (subscale ERQ) | 2 VS 1 | 265 | 0.0356 | -0.0584 | 0.1660 | 0.4608 | -0.1816 | -0.9208 | 0.5165 | 0.6544 | -0.1460 | -0.8902 | 0.5499 | 0.7204 | 0 |
|  | 3 VS 1 | 265 | 0.0047 | -0.1766 | 0.2039 | 0.9344 | 0.3861 | -0.8412 | 1.4697 | 0.5068 | 0.3908 | -0.8665 | 1.5012 | 0.5080 | 0 |
|  | 3 VS 2 | 265 | -0.0309 | -0.2404 | 0.1619 | 0.7356 | 0.5677 | -0.7339 | 1.7265 | 0.3688 | 0.5368 | -0.7901 | 1.7126 | 0.3996 | 0 |
| H10 Expressive supression (subscale ERQ) | 2 VS 1 | 265 | 0.0028 | -0.1035 | 0.1261 | 0.9480 | -0.1488 | -0.8747 | 0.5400 | 0.6948 | -0.1460 | -0.8902 | 0.5499 | 0.7200 | 0 |
|  | 3 VS 1 | 265 | -0.0409 | -0.2629 | 0.1544 | 0.6540 | 0.4317 | -0.8355 | 1.5455 | 0.4680 | 0.3908 | -0.8665 | 1.5012 | 0.5080 | 0 |
|  | 3 VS 2 | 265 | -0.0437 | -0.2737 | 0.1550 | 0.6432 | 0.5805 | -0.7569 | 1.7866 | 0.3680 | 0.5368 | -0.7901 | 1.7126 | 0.3996 | 0 |
| H11 Depression (total CDSS) | 2 VS 1 | 260 | 0.1185 | -0.1364 | 0.3897 | 0.3544 | -0.2945 | -1.0488 | 0.3858 | 0.4192 | -0.1760 | -0.9913 | 0.5436 | 0.6820 | 0 |
|  | 3 VS 1 | 260 | 0.4381 | -0.0320 | 0.9678 | 0.0668 | -0.0698 | -1.1796 | 0.9593 | 0.9256 | 0.3683 | -0.8791 | 1.4709 | 0.5288 | 0 |
|  | 3 VS 2 | 260 | 0.3196 | -0.1732 | 0.8708 | 0.2024 | 0.2248 | -0.9380 | 1.3099 | 0.6972 | 0.5443 | -0.7851 | 1.7528 | 0.4060 | 0 |
| H12 Sleep disturbances (total PSQI) | 2 VS 1 | 261 | 0.2255 | 0.0590 | 0.4525 | **0.0020** | -0.3065 | -1.0538 | 0.3899 | 0.4152 | -0.0810 | -0.8189 | 0.6272 | 0.8476 | **1** |
|  | 3 VS 1 | 261 | 0.2280 | -0.0150 | 0.5494 | 0.0640 | 0.0869 | -1.1017 | 1.1785 | 0.8320 | 0.3148 | -0.9514 | 1.4558 | 0.5732 | 0 |
|  | 3 VS 2 | 261 | 0.0025 | -0.2802 | 0.2571 | 0.9800 | 0.3934 | -0.8307 | 1.6105 | 0.5132 | 0.3959 | -0.9360 | 1.6781 | 0.5380 | 0 |
| **Supplement 6.** Point estimates and uncertainty estimates for total, direct, and indirect effects operationalizing outcome as sum score of PSYRATS-AH items 6,7,8,9,11. Abbreviations: H#: Hypothesis no.; Class 1: 'variable severity'; Class 2: 'severe neglect and emotional abuse'; Class 3: 'severe poly-trauma'. Indirect: Indirect effect; Direct: Direct effect; Total: Total effect; Lower/upper: lower and upper uncertainty estimates; bh_significant: Benjamini-Hochberg correction (0=non-significant after correction; 1= significant after correction); n/a= H1 cannot be computed as PSYRATS-AH items for negative content are redundant with the outcome operationalization in this model. Measures: Approve: Approve-Voices; BAVQ-R: Beliefs About Voices Questionnaire - Revised; CTQ: Childhood Trauma Questionnaire; BCSS: Brief Core Schema Scale; CDSS: Calgary Depression Scale for Schizophrenia; ERQ: Emotion Regulation Questionnaire; PSQI; Pittsburgh Sleep Quality Index; VPDS: Voice Power Differential Scale. Significance level: p < .05 (highlighted). All CI set at 95%. | | | | | | | | | | | | | | | |

| **Supplement 7.** Point estimates and uncertainty estimates for total, direct, and indirect effects operationalizing outcome as sum score of PSYRATS-AH items 6,7,8,9. | | | | | | | | | | | | | | | |
| --- | --- | --- | --- | --- | --- | --- | --- | --- | --- | --- | --- | --- | --- | --- | --- |
|  | Class | N | Indirect | Lower | Upper | P | Direct | Lower | Upper | P | Total | Lower | Upper | P | bh_significant |
| H1 | n/a | n/a | n/a | n/a | n/a | n/a | n/a | n/a | n/a | n/a | n/a | n/a | n/a | n/a | n/a |
| H2 Persecutory belief factor (subscale BAVQ-R) | 2 VS 1 | 265 | 0.0898 | -0.2413 | 0.4406 | 0.6152 | -0.1600 | -0.7409 | 0.3757 | 0.5832 | -0.0702 | -0.7206 | 0.5560 | 0.8384 | 0 |
|  | 3 VS 1 | 265 | 0.5832 | 0.0067 | 1.1670 | **0.0484** | -0.1892 | -1.1075 | 0.5919 | 0.6980 | 0.3940 | -0.6983 | 1.3320 | 0.4340 | 0 |
|  | 3 VS 2 | 265 | 0.4935 | -0.0892 | 1.0885 | 0.0928 | -0.0292 | -1.0016 | 0.8378 | 0.9704 | 0.4643 | -0.7008 | 1.4836 | 0.3684 | 0 |
| H3 Voice power differential (total VPDS) | 2 VS 1 | 263 | 0.1383 | -0.0897 | 0.3818 | 0.2328 | -0.1607 | -0.7673 | 0.3919 | 0.5940 | -0.0224 | -0.6584 | 0.5777 | 0.9436 | 0 |
|  | 3 VS 1 | 263 | 0.3714 | 0.0148 | 0.7905 | **0.0408** | 0.1407 | -0.8160 | 0.9177 | 0.7040 | 0.5121 | -0.5493 | 1.4139 | 0.2944 | 0 |
|  | 3 VS 2 | 263 | 0.2331 | -0.1221 | 0.6446 | 0.1944 | 0.3014 | -0.7247 | 1.1789 | 0.4920 | 0.5345 | -0.5642 | 1.5040 | 0.3024 | 0 |
| H4 Assertive relating (subscale Approve) | 2 VS 1 | 265 | 0.0460 | -0.0962 | 0.2078 | 0.5160 | -0.1994 | -0.8084 | 0.3810 | 0.5228 | -0.1534 | -0.7799 | 0.4360 | 0.6500 | 0 |
|  | 3 VS 1 | 265 | -0.1427 | -0.4441 | 0.1215 | 0.2676 | 0.5618 | -0.6129 | 1.5477 | 0.2748 | 0.4191 | -0.6603 | 1.3298 | 0.3728 | 0 |
|  | 3 VS 2 | 265 | -0.1886 | -0.5227 | 0.0862 | 0.1692 | 0.7611 | -0.4632 | 1.8385 | 0.1880 | 0.5725 | -0.5425 | 1.5509 | 0.2816 | 0 |
| H5 Aggressive responding (subscale Approve) | 2 VS 1 | 262 | -0.0598 | -0.1795 | 0.0130 | 0.1184 | -0.0792 | -0.7478 | 0.5710 | 0.8224 | -0.1390 | -0.8215 | 0.4959 | 0.6836 | 0 |
|  | 3 VS 1 | 262 | -0.2059 | -0.5406 | 0.0069 | 0.0592 | 0.7559 | -0.4420 | 1.7792 | 0.1976 | 0.5500 | -0.5366 | 1.4712 | 0.2884 | 0 |
|  | 3 VS 2 | 262 | -0.1461 | -0.4474 | 0.0291 | 0.1148 | 0.8351 | -0.3622 | 1.9062 | 0.1644 | 0.6890 | -0.4503 | 1.7028 | 0.2108 | 0 |
| H6 Passive/submissive responding (subsacle Approve) | 2 VS 1 | 264 | 0.0328 | -0.1463 | 0.2119 | 0.7200 | -0.1485 | -0.7754 | 0.4230 | 0.6060 | -0.1157 | -0.7930 | 0.4823 | 0.7000 | 0 |
|  | 3 VS 1 | 264 | 0.2973 | -0.0346 | 0.7167 | 0.0824 | 0.2418 | -0.8113 | 1.1128 | 0.5900 | 0.5391 | -0.5556 | 1.4675 | 0.2992 | 0 |
|  | 3 VS 2 | 264 | 0.2645 | -0.0733 | 0.6764 | 0.1292 | 0.3902 | -0.6841 | 1.3444 | 0.4296 | 0.6548 | -0.4682 | 1.6510 | 0.2360 | 0 |
| H7 Negative other-beliefs (subscale BCSS) | 2 VS 1 | 261 | 0.0178 | -0.0249 | 0.0928 | 0.5044 | -0.0888 | -0.7296 | 0.5362 | 0.8248 | -0.0710 | -0.7156 | 0.5492 | 0.8680 | 0 |
|  | 3 VS 1 | 261 | 0.0168 | -0.0706 | 0.1335 | 0.7560 | 0.4847 | -0.5723 | 1.4172 | 0.3408 | 0.5015 | -0.5559 | 1.4265 | 0.3268 | 0 |
|  | 3 VS 2 | 261 | -0.0010 | -0.1044 | 0.1006 | 0.9656 | 0.5735 | -0.5232 | 1.5660 | 0.2972 | 0.5725 | -0.5419 | 1.5700 | 0.3012 | 0 |
| H8 Negative self-beliefs (subscale BCSS) | 2 VS 1 | 261 | 0.1534 | -0.0148 | 0.3863 | 0.0824 | -0.1922 | -0.8388 | 0.3943 | 0.5624 | -0.0388 | -0.6931 | 0.5917 | 0.9348 | 0 |
|  | 3 VS 1 | 261 | 0.2454 | -0.0100 | 0.5928 | 0.0640 | 0.2883 | -0.7950 | 1.1722 | 0.5288 | 0.5337 | -0.5519 | 1.4684 | 0.2824 | 0 |
|  | 3 VS 2 | 261 | 0.0920 | -0.1984 | 0.4140 | 0.5360 | 0.4806 | -0.6483 | 1.4286 | 0.3440 | 0.5725 | -0.5671 | 1.5472 | 0.2908 | 0 |
| H9 Cognitive reappraisal (subscale ERQ) | 2 VS 1 | 265 | 0.0272 | -0.0453 | 0.1363 | 0.5048 | -0.0926 | -0.7333 | 0.5260 | 0.7912 | -0.0654 | -0.7036 | 0.5497 | 0.8644 | 0 |
|  | 3 VS 1 | 265 | 0.0036 | -0.1433 | 0.1654 | 0.9328 | 0.5035 | -0.5527 | 1.3999 | 0.3316 | 0.5071 | -0.5761 | 1.4409 | 0.3324 | 0 |
|  | 3 VS 2 | 265 | -0.0236 | -0.1956 | 0.1308 | 0.7596 | 0.5961 | -0.5283 | 1.5634 | 0.2732 | 0.5725 | -0.5598 | 1.5573 | 0.2868 | 0 |
| H10 Expressive supression (subscale ERQ) | 2 VS 1 | 265 | 0.0030 | -0.1102 | 0.1329 | 0.9488 | -0.0684 | -0.7048 | 0.5351 | 0.8420 | -0.0654 | -0.7036 | 0.5497 | 0.8644 | 0 |
|  | 3 VS 1 | 265 | -0.0449 | -0.2750 | 0.1659 | 0.6460 | 0.5520 | -0.5197 | 1.4647 | 0.2876 | 0.5071 | -0.5761 | 1.4409 | 0.3328 | 0 |
|  | 3 VS 2 | 265 | -0.0479 | -0.2903 | 0.1638 | 0.6356 | 0.6204 | -0.5170 | 1.6017 | 0.2596 | 0.5725 | -0.5598 | 1.5573 | 0.2868 | 0 |
| H11 Depression (total CDSS) | 2 VS 1 | 260 | 0.1058 | -0.1185 | 0.3502 | 0.3544 | -0.1716 | -0.8126 | 0.4193 | 0.5952 | -0.0658 | -0.7534 | 0.5615 | 0.8680 | 0 |
|  | 3 VS 1 | 260 | 0.3911 | -0.0278 | 0.8718 | 0.0668 | 0.1118 | -0.8319 | 0.9726 | 0.7944 | 0.5029 | -0.5504 | 1.4404 | 0.3260 | 0 |
|  | 3 VS 2 | 260 | 0.2853 | -0.1537 | 0.7835 | 0.2024 | 0.2834 | -0.7028 | 1.1947 | 0.5544 | 0.5687 | -0.5684 | 1.5716 | 0.2936 | 0 |
| H12 Sleep disturbances (total PSQI) | 2 VS 1 | 261 | 0.2197 | 0.0641 | 0.4327 | **0.0016** | -0.2251 | -0.8571 | 0.3874 | 0.4864 | -0.0054 | -0.6257 | 0.6206 | 0.9992 | **1** |
|  | 3 VS 1 | 261 | 0.2221 | -0.0143 | 0.5266 | 0.0636 | 0.1868 | -0.7905 | 1.0662 | 0.6576 | 0.4089 | -0.6495 | 1.3381 | 0.4000 | 0 |
|  | 3 VS 2 | 261 | 0.0024 | -0.2702 | 0.2479 | 0.9796 | 0.4118 | -0.5826 | 1.3704 | 0.3920 | 0.4142 | -0.6896 | 1.4263 | 0.4256 | 0 |
| **Supplement 7.** Point estimates and uncertainty estimates for total, direct, and indirect effects operationalizing outcome as sum score of PSYRATS-AH items 6,7,8,9. Abbreviations: H#: Hypothesis no.; Class 1: 'variable severity'; Class 2: 'severe neglect and emotional abuse'; Class 3: 'severe poly-trauma'. Indirect: Indirect effect; Direct: Direct effect; Total: Total effect; Lower/upper: lower and upper uncertainty estimates; bh_significant: Benjamini-Hochberg correction (0=non-significant after correction; 1= significant after correction); n/a= H1 cannot be computed as PSYRATS-AH items for negative content are redundant with the outcome operationalization in this model. Measures: Approve: Approve-Voices; BAVQ-R: Beliefs About Voices Questionnaire - Revised; CTQ: Childhood Trauma Questionnaire; BCSS: Brief Core Schema Scale; CDSS: Calgary Depression Scale for Schizophrenia; ERQ: Emotion Regulation Questionnaire; PSQI; Pittsburgh Sleep Quality Index; VPDS: Voice Power Differential Scale. Significance level: p < .05 (highlighted). All CI set at 95%. | | | | | | | | | | | | | | | |

| **Supplement 8.** Point estimates and uncertainty estimates for total, direct, and indirect effects operationalizing outcome as sum score of PSYRATS-AH items 8,9,11. | | | | | | | | | | | | | | | |
| --- | --- | --- | --- | --- | --- | --- | --- | --- | --- | --- | --- | --- | --- | --- | --- |
|  | Class | N | Indirect | Lower | Upper | P | Direct | Lower | Upper | P | Total | Lower | Upper | P | bh_significant |
| H1 Negative voice-content (items 6+7 PSYRATS-AHS) | 2 VS 1 | 266 | -0.0178 | -0.3219 | 0.2323 | 0.9120 | -0.1114 | -0.4709 | 0.2513 | 0.5748 | -0.1292 | -0.5964 | 0.3049 | 0.6016 | 0 |
|  | 3 VS 1 | 266 | 0.1885 | -0.2026 | 0.5219 | 0.3004 | -0.0601 | -0.6211 | 0.5029 | 0.8440 | 0.1284 | -0.6901 | 0.8492 | 0.7108 | 0 |
|  | 3 VS 2 | 266 | 0.2063 | -0.1956 | 0.6069 | 0.2872 | 0.0513 | -0.5576 | 0.6422 | 0.8504 | 0.2576 | -0.6186 | 1.0346 | 0.5228 | 0 |
| H2 Persecutory belief factor (subscale BAVQ-R) | 2 VS 1 | 265 | 0.0511 | -0.1383 | 0.2523 | 0.6152 | -0.1803 | -0.6171 | 0.2165 | 0.3992 | -0.1292 | -0.6063 | 0.3026 | 0.5816 | 0 |
|  | 3 VS 1 | 265 | 0.3322 | 0.0043 | 0.6667 | **0.0484** | -0.2470 | -0.9320 | 0.4047 | 0.4872 | 0.0851 | -0.7483 | 0.8527 | 0.7956 | 0 |
|  | 3 VS 2 | 265 | 0.2810 | -0.0493 | 0.6241 | 0.0928 | -0.0668 | -0.8082 | 0.6512 | 0.8876 | 0.2143 | -0.6668 | 1.0445 | 0.5856 | 0 |
| H3 Voice power differential (total VPDS) | 2 VS 1 | 263 | 0.0987 | -0.0633 | 0.2723 | 0.2328 | -0.2053 | -0.6407 | 0.2066 | 0.3376 | -0.1066 | -0.5666 | 0.3266 | 0.6448 | 0 |
|  | 3 VS 1 | 263 | 0.2650 | 0.0102 | 0.5649 | **0.0408** | -0.1362 | -0.8540 | 0.4839 | 0.7172 | 0.1289 | -0.6661 | 0.8415 | 0.6908 | 0 |
|  | 3 VS 2 | 263 | 0.1664 | -0.0854 | 0.4564 | 0.1944 | 0.0691 | -0.7105 | 0.7557 | 0.8088 | 0.2355 | -0.6159 | 1.0155 | 0.5392 | 0 |
| H4 Assertive relating (subscale Approve) | 2 VS 1 | 265 | 0.0336 | -0.0699 | 0.1510 | 0.5160 | -0.2066 | -0.6492 | 0.2173 | 0.3568 | -0.1730 | -0.6258 | 0.2520 | 0.4512 | 0 |
|  | 3 VS 1 | 265 | -0.1044 | -0.3238 | 0.0881 | 0.2676 | 0.1891 | -0.6602 | 0.9628 | 0.6052 | 0.0846 | -0.7202 | 0.8053 | 0.7800 | 0 |
|  | 3 VS 2 | 265 | -0.1381 | -0.3697 | 0.0620 | 0.1692 | 0.3957 | -0.5046 | 1.2453 | 0.3484 | 0.2576 | -0.5836 | 1.0530 | 0.5012 | 0 |
| H5 Aggressive responding (subscale Approve) | 2 VS 1 | 262 | -0.0382 | -0.1202 | 0.0107 | 0.1524 | -0.1356 | -0.6145 | 0.3012 | 0.5836 | -0.1738 | -0.6600 | 0.2682 | 0.4688 | 0 |
|  | 3 VS 1 | 262 | -0.1314 | -0.3697 | 0.0191 | 0.0972 | 0.2933 | -0.5912 | 1.1136 | 0.4684 | 0.1618 | -0.6710 | 0.9121 | 0.6564 | 0 |
|  | 3 VS 2 | 262 | -0.0933 | -0.3070 | 0.0251 | 0.1512 | 0.4289 | -0.4838 | 1.3140 | 0.3312 | 0.3356 | -0.5346 | 1.1676 | 0.4176 | 0 |
| H6 Passive/submissive responding (subsacle Approve) | 2 VS 1 | 264 | 0.0254 | -0.1155 | 0.1560 | 0.7200 | -0.1795 | -0.6306 | 0.2363 | 0.4008 | -0.1541 | -0.6364 | 0.2773 | 0.4900 | 0 |
|  | 3 VS 1 | 264 | 0.2298 | -0.0288 | 0.5329 | 0.0824 | -0.0744 | -0.8548 | 0.6082 | 0.8708 | 0.1554 | -0.6994 | 0.8929 | 0.6644 | 0 |
|  | 3 VS 2 | 264 | 0.2045 | -0.0549 | 0.5155 | 0.1292 | 0.1050 | -0.7019 | 0.8529 | 0.7440 | 0.3095 | -0.5566 | 1.1285 | 0.4416 | 0 |
| H7 Negative other-beliefs (subscale BCSS) | 2 VS 1 | 261 | 0.0165 | -0.0178 | 0.0765 | 0.4152 | -0.1444 | -0.6049 | 0.2951 | 0.5684 | -0.1280 | -0.5909 | 0.3139 | 0.6168 | 0 |
|  | 3 VS 1 | 261 | 0.0156 | -0.0539 | 0.1166 | 0.6908 | 0.1140 | -0.6549 | 0.8301 | 0.7504 | 0.1296 | -0.6410 | 0.8525 | 0.7272 | 0 |
|  | 3 VS 2 | 261 | -0.0009 | -0.0860 | 0.0883 | 0.9668 | 0.2585 | -0.5831 | 1.0396 | 0.5296 | 0.2576 | -0.6029 | 1.0604 | 0.5408 | 0 |
| H8 Negative self-beliefs (subscale BCSS) | 2 VS 1 | 261 | 0.0821 | -0.0080 | 0.2162 | 0.0832 | -0.1842 | -0.6552 | 0.2490 | 0.4392 | -0.1022 | -0.5684 | 0.3414 | 0.6976 | 0 |
|  | 3 VS 1 | 261 | 0.1313 | -0.0054 | 0.3393 | 0.0656 | 0.0241 | -0.7748 | 0.7310 | 0.9168 | 0.1554 | -0.6497 | 0.8894 | 0.6676 | 0 |
|  | 3 VS 2 | 261 | 0.0492 | -0.1068 | 0.2338 | 0.5352 | 0.2084 | -0.6234 | 0.9513 | 0.5844 | 0.2576 | -0.6050 | 1.0560 | 0.5272 | 0 |
| H9 Cognitive reappraisal (subscale ERQ) | 2 VS 1 | 265 | 0.0251 | -0.0393 | 0.1145 | 0.4392 | -0.1478 | -0.6207 | 0.2886 | 0.5296 | -0.1226 | -0.5937 | 0.3137 | 0.5992 | 0 |
|  | 3 VS 1 | 265 | 0.0033 | -0.1122 | 0.1416 | 0.9320 | 0.1316 | -0.6675 | 0.8317 | 0.7124 | 0.1349 | -0.6798 | 0.8496 | 0.7056 | 0 |
|  | 3 VS 2 | 265 | -0.0218 | -0.1574 | 0.1119 | 0.7228 | 0.2794 | -0.5662 | 1.0450 | 0.4876 | 0.2576 | -0.6070 | 1.0579 | 0.5260 | 0 |
| H10 Expressive supression (subscale ERQ) | 2 VS 1 | 265 | 0.0006 | -0.0346 | 0.0477 | 0.9380 | -0.1233 | -0.5909 | 0.3083 | 0.5912 | -0.1226 | -0.5937 | 0.3137 | 0.5992 | 0 |
|  | 3 VS 1 | 265 | -0.0096 | -0.0939 | 0.0632 | 0.8256 | 0.1445 | -0.6811 | 0.8697 | 0.6992 | 0.1349 | -0.6798 | 0.8496 | 0.7052 | 0 |
|  | 3 VS 2 | 265 | -0.0102 | -0.0985 | 0.0591 | 0.7892 | 0.2678 | -0.6022 | 1.0698 | 0.5124 | 0.2576 | -0.6070 | 1.0579 | 0.5260 | 0 |
| H11 Depression (total CDSS) | 2 VS 1 | 260 | 0.0593 | -0.0692 | 0.1978 | 0.3544 | -0.2230 | -0.7086 | 0.2076 | 0.3288 | -0.1637 | -0.6726 | 0.2865 | 0.5092 | 0 |
|  | 3 VS 1 | 260 | 0.2192 | -0.0159 | 0.5080 | 0.0668 | -0.1090 | -0.8606 | 0.5897 | 0.7832 | 0.1101 | -0.7167 | 0.8280 | 0.7644 | 0 |
|  | 3 VS 2 | 260 | 0.1599 | -0.0841 | 0.4526 | 0.2024 | 0.1140 | -0.6773 | 0.8433 | 0.7608 | 0.2738 | -0.6021 | 1.0700 | 0.5160 | 0 |
| H12 Sleep disturbances (total PSQI) | 2 VS 1 | 261 | 0.1177 | 0.0240 | 0.2477 | **0.0056** | -0.2056 | -0.6783 | 0.2316 | 0.3784 | -0.0879 | -0.5565 | 0.3455 | 0.7320 | **1** |
|  | 3 VS 1 | 261 | 0.1190 | -0.0073 | 0.3011 | 0.0676 | -0.0268 | -0.7952 | 0.7009 | 0.9868 | 0.0922 | -0.7202 | 0.8589 | 0.7716 | 0 |
|  | 3 VS 2 | 261 | 0.0013 | -0.1469 | 0.1364 | 0.9820 | 0.1789 | -0.6200 | 0.9669 | 0.6272 | 0.1801 | -0.6903 | 1.0135 | 0.6424 | 0 |
| **Supplement 8.** Point estimates and uncertainty estimates for total, direct, and indirect effects operationalizing outcome as sum score of PSYRATS-AH items 8,9,11. Abbreviations: H#: Hypothesis no.; Class 1: 'variable severity'; Class 2: 'severe neglect and emotional abuse'; Class 3: 'severe poly-trauma'. Indirect: Indirect effect; Direct: Direct effect; Total: Total effect; Lower/upper: lower and upper uncertainty estimates; bh_significant: Benjamini-Hochberg correction (0=non-significant after correction; 1= significant after correction). Measures: Approve: Approve-Voices; BAVQ-R: Beliefs About Voices Questionnaire - Revised; CTQ: Childhood Trauma Questionnaire; BCSS: Brief Core Schema Scale; CDSS: Calgary Depression Scale for Schizophrenia; ERQ: Emotion Regulation Questionnaire; PSQI; Pittsburgh Sleep Quality Index; VPDS: Voice Power Differential Scale. Significance level: p < .05 (highlighted). All CI set at 95%. | | | | | | | | | | | | | | | |

| **Supplement 9.** Point estimates and uncertainty estimates for total, direct, and indirect effects operationalizing outcome as sum score of PSYRATS-AH item 8. | | | | | | | | | | | | | | | |
| --- | --- | --- | --- | --- | --- | --- | --- | --- | --- | --- | --- | --- | --- | --- | --- |
|  | Class | N | Indirect | Lower | Upper | P | Direct | Lower | Upper | P | Total | Lower | Upper | P | bh_significant |
| H1 Negative voice-content (items 6+7 PSYRATS-AHS) | 2 VS 1 | 266 | -0.0082 | -0.1467 | 0.1083 | 0.9120 | -0.0058 | -0.1723 | 0.1611 | 0.9804 | -0.0140 | -0.2261 | 0.1897 | 0.9276 | 0 |
|  | 3 VS 1 | 266 | 0.0867 | -0.0954 | 0.2426 | 0.3004 | -0.0055 | -0.2314 | 0.2005 | 0.9956 | 0.0813 | -0.2821 | 0.3845 | 0.5852 | 0 |
|  | 3 VS 2 | 266 | 0.0949 | -0.0923 | 0.2727 | 0.2872 | 0.0003 | -0.2447 | 0.2271 | 0.9880 | 0.0952 | -0.2897 | 0.4235 | 0.5656 | 0 |
| H2 Persecutory belief factor (subscale BAVQ-R) | 2 VS 1 | 265 | 0.0212 | -0.0574 | 0.1059 | 0.6152 | -0.0352 | -0.2312 | 0.1485 | 0.7332 | -0.0140 | -0.2245 | 0.1811 | 0.9056 | 0 |
|  | 3 VS 1 | 265 | 0.1376 | 0.0015 | 0.2835 | **0.0484** | -0.0801 | -0.4004 | 0.1899 | 0.6380 | 0.0574 | -0.3197 | 0.3741 | 0.7032 | 0 |
|  | 3 VS 2 | 265 | 0.1164 | -0.0202 | 0.2666 | 0.0928 | -0.0450 | -0.3879 | 0.2560 | 0.8236 | 0.0714 | -0.3238 | 0.4149 | 0.6604 | 0 |
| H3 Voice power differential (total VPDS) | 2 VS 1 | 263 | 0.0300 | -0.0193 | 0.0857 | 0.2328 | -0.0381 | -0.2470 | 0.1591 | 0.7036 | -0.0081 | -0.2184 | 0.1938 | 0.9392 | 0 |
|  | 3 VS 1 | 263 | 0.0807 | 0.0030 | 0.1771 | **0.0408** | 0.0016 | -0.3235 | 0.2752 | 0.9284 | 0.0823 | -0.2686 | 0.3802 | 0.5736 | 0 |
|  | 3 VS 2 | 263 | 0.0506 | -0.0257 | 0.1420 | 0.1944 | 0.0397 | -0.3042 | 0.3456 | 0.7748 | 0.0904 | -0.2788 | 0.4165 | 0.5784 | 0 |
| H4 Assertive relating (subscale Approve) | 2 VS 1 | 265 | 0.0159 | -0.0331 | 0.0694 | 0.5160 | -0.0514 | -0.2503 | 0.1350 | 0.6008 | -0.0355 | -0.2414 | 0.1583 | 0.7396 | 0 |
|  | 3 VS 1 | 265 | -0.0494 | -0.1522 | 0.0404 | 0.2676 | 0.1091 | -0.2791 | 0.4417 | 0.5000 | 0.0597 | -0.3076 | 0.3717 | 0.6700 | 0 |
|  | 3 VS 2 | 265 | -0.0653 | -0.1746 | 0.0303 | 0.1692 | 0.1605 | -0.2318 | 0.5133 | 0.3708 | 0.0952 | -0.2780 | 0.4279 | 0.5512 | 0 |
| H5 Aggressive responding (subscale Approve) | 2 VS 1 | 262 | -0.0208 | -0.0650 | 0.0035 | 0.1164 | -0.0056 | -0.2257 | 0.2064 | 0.9676 | -0.0265 | -0.2445 | 0.1827 | 0.8104 | 0 |
|  | 3 VS 1 | 262 | -0.0717 | -0.1866 | 0.0021 | 0.0612 | 0.1715 | -0.2165 | 0.5107 | 0.3472 | 0.0998 | -0.2560 | 0.4150 | 0.5188 | 0 |
|  | 3 VS 2 | 262 | -0.0509 | -0.1525 | 0.0084 | 0.1168 | 0.1771 | -0.2224 | 0.5377 | 0.3468 | 0.1262 | -0.2531 | 0.4707 | 0.4728 | 0 |
| H6 Passive/submissive responding (subsacle Approve) | 2 VS 1 | 264 | 0.0063 | -0.0300 | 0.0415 | 0.7208 | -0.0292 | -0.2363 | 0.1675 | 0.7672 | -0.0229 | -0.2340 | 0.1763 | 0.8204 | 0 |
|  | 3 VS 1 | 264 | 0.0571 | -0.0069 | 0.1520 | 0.0840 | 0.0390 | -0.3072 | 0.3449 | 0.7860 | 0.0961 | -0.2614 | 0.4136 | 0.5484 | 0 |
|  | 3 VS 2 | 264 | 0.0508 | -0.0133 | 0.1431 | 0.1308 | 0.0683 | -0.2984 | 0.3962 | 0.6708 | 0.1190 | -0.2573 | 0.4523 | 0.4908 | 0 |
| H7 Negative other-beliefs (subscale BCSS) | 2 VS 1 | 261 | 0.0027 | -0.0121 | 0.0241 | 0.7516 | -0.0237 | -0.2357 | 0.1884 | 0.8632 | -0.0210 | -0.2311 | 0.1903 | 0.8820 | 0 |
|  | 3 VS 1 | 261 | 0.0025 | -0.0223 | 0.0324 | 0.8720 | 0.0717 | -0.2801 | 0.3813 | 0.6624 | 0.0742 | -0.2755 | 0.3823 | 0.6492 | 0 |
|  | 3 VS 2 | 261 | -0.0001 | -0.0286 | 0.0250 | 0.9768 | 0.0954 | -0.2771 | 0.4197 | 0.5964 | 0.0952 | -0.2788 | 0.4228 | 0.6044 | 0 |
| H8 Negative self-beliefs (subscale BCSS) | 2 VS 1 | 261 | 0.0367 | -0.0038 | 0.0994 | 0.0856 | -0.0449 | -0.2618 | 0.1587 | 0.6884 | -0.0081 | -0.2245 | 0.1940 | 0.9620 | 0 |
|  | 3 VS 1 | 261 | 0.0588 | -0.0026 | 0.1514 | 0.0656 | 0.0283 | -0.3306 | 0.3257 | 0.8244 | 0.0871 | -0.2727 | 0.3958 | 0.5664 | 0 |
|  | 3 VS 2 | 261 | 0.0220 | -0.0500 | 0.1050 | 0.5368 | 0.0732 | -0.2961 | 0.3941 | 0.6540 | 0.0952 | -0.2785 | 0.4296 | 0.5924 | 0 |
| H9 Cognitive reappraisal (subscale ERQ) | 2 VS 1 | 265 | 0.0110 | -0.0183 | 0.0488 | 0.4584 | -0.0276 | -0.2362 | 0.1732 | 0.8076 | -0.0166 | -0.2280 | 0.1839 | 0.8920 | 0 |
|  | 3 VS 1 | 265 | 0.0015 | -0.0530 | 0.0589 | 0.9272 | 0.0772 | -0.2812 | 0.3821 | 0.6092 | 0.0786 | -0.2861 | 0.3896 | 0.6084 | 0 |
|  | 3 VS 2 | 265 | -0.0095 | -0.0726 | 0.0482 | 0.7364 | 0.1048 | -0.2711 | 0.4253 | 0.5264 | 0.0952 | -0.2825 | 0.4287 | 0.5628 | 0 |
| H10 Expressive supression (subscale ERQ) | 2 VS 1 | 265 | 0.0010 | -0.0365 | 0.0415 | 0.9488 | -0.0176 | -0.2243 | 0.1775 | 0.8748 | -0.0166 | -0.2280 | 0.1839 | 0.8920 | 0 |
|  | 3 VS 1 | 265 | -0.0144 | -0.0879 | 0.0539 | 0.6460 | 0.0931 | -0.2655 | 0.3992 | 0.5596 | 0.0786 | -0.2861 | 0.3896 | 0.6084 | 0 |
|  | 3 VS 2 | 265 | -0.0154 | -0.0916 | 0.0527 | 0.6352 | 0.1107 | -0.2630 | 0.4346 | 0.5080 | 0.0952 | -0.2825 | 0.4287 | 0.5624 | 0 |
| H11 Depression (total CDSS) | 2 VS 1 | 260 | 0.0270 | -0.0313 | 0.0889 | 0.3548 | -0.0476 | -0.2610 | 0.1574 | 0.6752 | -0.0206 | -0.2436 | 0.1857 | 0.8664 | 0 |
|  | 3 VS 1 | 260 | 0.0997 | -0.0071 | 0.2260 | 0.0672 | -0.0228 | -0.3408 | 0.2751 | 0.9068 | 0.0769 | -0.2706 | 0.3906 | 0.6436 | 0 |
|  | 3 VS 2 | 260 | 0.0728 | -0.0381 | 0.2025 | 0.2028 | 0.0248 | -0.3206 | 0.3356 | 0.8760 | 0.0976 | -0.2755 | 0.4316 | 0.5804 | 0 |
| H12 Sleep disturbances (total PSQI) | 2 VS 1 | 261 | 0.0580 | 0.0128 | 0.1202 | **0.0060** | -0.0688 | -0.2791 | 0.1421 | 0.5456 | -0.0107 | -0.2145 | 0.1975 | 0.9160 | **1** |
|  | 3 VS 1 | 261 | 0.0586 | -0.0040 | 0.1457 | 0.0680 | -0.0028 | -0.3428 | 0.3025 | 0.9632 | 0.0558 | -0.3043 | 0.3772 | 0.6972 | 0 |
|  | 3 VS 2 | 261 | 0.0006 | -0.0721 | 0.0672 | 0.9800 | 0.0659 | -0.2869 | 0.3913 | 0.6848 | 0.0666 | -0.3201 | 0.4112 | 0.6968 | 0 |
| **Supplement 9.** Point estimates and uncertainty estimates for total, direct, and indirect effects operationalizing outcome as sum score of PSYRATS-AH item 8. Abbreviations: H#: Hypothesis no.; Class 1: 'variable severity'; Class 2: 'severe neglect and emotional abuse'; Class 3: 'severe poly-trauma'. Indirect: Indirect effect; Direct: Direct effect; Total: Total effect; Lower/upper: lower and upper uncertainty estimates; bh_significant: Benjamini-Hochberg correction (0=non-significant after correction; 1= significant after correction). Measures: Approve: Approve-Voices; BAVQ-R: Beliefs About Voices Questionnaire - Revised; CTQ: Childhood Trauma Questionnaire; BCSS: Brief Core Schema Scale; CDSS: Calgary Depression Scale for Schizophrenia; ERQ: Emotion Regulation Questionnaire; PSQI; Pittsburgh Sleep Quality Index; VPDS: Voice Power Differential Scale. Significance level: p < .05 (highlighted). All CI set at 95%. | | | | | | | | | | | | | | | |

| **Supplement 10.** Point estimates and uncertainty estimates for total, direct, and indirect effects operationalizing outcome as sum score of PSYRATS-AH item 9. | | | | | | | | | | | | | | | |
| --- | --- | --- | --- | --- | --- | --- | --- | --- | --- | --- | --- | --- | --- | --- | --- |
|  | Class | N | Indirect | Lower | Upper | P | Direct | Lower | Upper | P | Total | Lower | Upper | P | bh_significant |
| H1 Negative voice-content (items 6+7 PSYRATS-AHS) | 2 VS 1 | 266 | -0.0068 | -0.1117 | 0.0960 | 0.9120 | -0.0254 | -0.2009 | 0.1449 | 0.7504 | -0.0321 | -0.2279 | 0.1580 | 0.7500 | 0 |
|  | 3 VS 1 | 266 | 0.0718 | -0.0763 | 0.2085 | 0.3004 | 0.0941 | -0.2168 | 0.3988 | 0.5244 | 0.1659 | -0.1867 | 0.4907 | 0.3164 | 0 |
|  | 3 VS 2 | 266 | 0.0786 | -0.0778 | 0.2241 | 0.2868 | 0.1195 | -0.1949 | 0.4428 | 0.4368 | 0.1981 | -0.1672 | 0.5336 | 0.2612 | 0 |
| H2 Persecutory belief factor (subscale BAVQ-R) | 2 VS 1 | 265 | 0.0196 | -0.0529 | 0.0993 | 0.6152 | -0.0517 | -0.2433 | 0.1313 | 0.5776 | -0.0321 | -0.2320 | 0.1618 | 0.7436 | 0 |
|  | 3 VS 1 | 265 | 0.1272 | 0.0015 | 0.2669 | **0.0484** | -0.0046 | -0.3363 | 0.3207 | 0.9796 | 0.1226 | -0.2274 | 0.4653 | 0.4644 | 0 |
|  | 3 VS 2 | 265 | 0.1076 | -0.0187 | 0.2457 | 0.0928 | 0.0471 | -0.2955 | 0.3838 | 0.7748 | 0.1548 | -0.2098 | 0.5021 | 0.3688 | 0 |
| H3 Voice power differential (total VPDS) | 2 VS 1 | 263 | 0.0353 | -0.0239 | 0.0976 | 0.2328 | -0.0437 | -0.2283 | 0.1330 | 0.6236 | -0.0084 | -0.2019 | 0.1754 | 0.9128 | 0 |
|  | 3 VS 1 | 263 | 0.0948 | 0.0037 | 0.2104 | **0.0408** | 0.0721 | -0.2529 | 0.3780 | 0.6456 | 0.1669 | -0.1705 | 0.4977 | 0.3168 | 0 |
|  | 3 VS 2 | 263 | 0.0595 | -0.0289 | 0.1715 | 0.1944 | 0.1158 | -0.2220 | 0.4345 | 0.4664 | 0.1752 | -0.1701 | 0.5169 | 0.3068 | 0 |
| H4 Assertive relating (subscale Approve) | 2 VS 1 | 265 | 0.0050 | -0.0123 | 0.0335 | 0.6052 | -0.0555 | -0.2498 | 0.1410 | 0.5680 | -0.0505 | -0.2464 | 0.1438 | 0.6020 | 0 |
|  | 3 VS 1 | 265 | -0.0155 | -0.0712 | 0.0204 | 0.4352 | 0.1631 | -0.1905 | 0.4986 | 0.3432 | 0.1475 | -0.1983 | 0.4720 | 0.3672 | 0 |
|  | 3 VS 2 | 265 | -0.0206 | -0.0870 | 0.0177 | 0.3528 | 0.2186 | -0.1524 | 0.5816 | 0.2260 | 0.1981 | -0.1542 | 0.5455 | 0.2496 | 0 |
| H5 Aggressive responding (subscale Approve) | 2 VS 1 | 262 | -0.0052 | -0.0379 | 0.0201 | 0.6492 | -0.0456 | -0.2459 | 0.1515 | 0.6468 | -0.0509 | -0.2540 | 0.1476 | 0.6104 | 0 |
|  | 3 VS 1 | 262 | -0.0181 | -0.1147 | 0.0610 | 0.6244 | 0.2306 | -0.1510 | 0.5823 | 0.2272 | 0.2125 | -0.1365 | 0.5390 | 0.2196 | 0 |
|  | 3 VS 2 | 262 | -0.0128 | -0.0928 | 0.0457 | 0.6544 | 0.2762 | -0.0980 | 0.6421 | 0.1500 | 0.2633 | -0.0930 | 0.6110 | 0.1416 | 0 |
| H6 Passive/submissive responding (subsacle Approve) | 2 VS 1 | 264 | 0.0111 | -0.0507 | 0.0704 | 0.7200 | -0.0491 | -0.2504 | 0.1308 | 0.5948 | -0.0380 | -0.2449 | 0.1479 | 0.6980 | 0 |
|  | 3 VS 1 | 264 | 0.1006 | -0.0124 | 0.2363 | 0.0824 | 0.1114 | -0.2169 | 0.4123 | 0.4580 | 0.2120 | -0.1292 | 0.5339 | 0.1996 | 0 |
|  | 3 VS 2 | 264 | 0.0895 | -0.0244 | 0.2308 | 0.1292 | 0.1605 | -0.1676 | 0.4766 | 0.3076 | 0.2500 | -0.0913 | 0.5881 | 0.1460 | 0 |
| H7 Negative other-beliefs (subscale BCSS) | 2 VS 1 | 261 | 0.0060 | -0.0105 | 0.0313 | 0.5272 | -0.0293 | -0.2295 | 0.1651 | 0.8092 | -0.0233 | -0.2195 | 0.1706 | 0.8520 | 0 |
|  | 3 VS 1 | 261 | 0.0057 | -0.0204 | 0.0501 | 0.7076 | 0.1691 | -0.1748 | 0.4826 | 0.3204 | 0.1748 | -0.1766 | 0.4977 | 0.3136 | 0 |
|  | 3 VS 2 | 261 | -0.0003 | -0.0329 | 0.0412 | 0.9932 | 0.1984 | -0.1523 | 0.5301 | 0.2804 | 0.1981 | -0.1593 | 0.5380 | 0.2868 | 0 |
| H8 Negative self-beliefs (subscale BCSS) | 2 VS 1 | 261 | 0.0252 | -0.0037 | 0.0746 | 0.1144 | -0.0421 | -0.2448 | 0.1554 | 0.7092 | -0.0168 | -0.2166 | 0.1788 | 0.9016 | 0 |
|  | 3 VS 1 | 261 | 0.0404 | -0.0046 | 0.1176 | 0.0976 | 0.1409 | -0.2050 | 0.4608 | 0.4168 | 0.1812 | -0.1663 | 0.5059 | 0.2956 | 0 |
|  | 3 VS 2 | 261 | 0.0151 | -0.0346 | 0.0797 | 0.5488 | 0.1829 | -0.1821 | 0.5101 | 0.3172 | 0.1981 | -0.1743 | 0.5392 | 0.2892 | 0 |
| H9 Cognitive reappraisal (subscale ERQ) | 2 VS 1 | 265 | 0.0058 | -0.0112 | 0.0368 | 0.5688 | -0.0312 | -0.2291 | 0.1588 | 0.7544 | -0.0254 | -0.2228 | 0.1671 | 0.8000 | 0 |
|  | 3 VS 1 | 265 | 0.0008 | -0.0331 | 0.0450 | 0.9032 | 0.1719 | -0.1778 | 0.4955 | 0.3120 | 0.1727 | -0.1811 | 0.4999 | 0.3076 | 0 |
|  | 3 VS 2 | 265 | -0.0050 | -0.0496 | 0.0338 | 0.8068 | 0.2031 | -0.1559 | 0.5485 | 0.2484 | 0.1981 | -0.1586 | 0.5508 | 0.2560 | 0 |
| H10 Expressive supression (subscale ERQ) | 2 VS 1 | 265 | -0.0001 | -0.0127 | 0.0186 | 0.9160 | -0.0253 | -0.2252 | 0.1675 | 0.7924 | -0.0254 | -0.2228 | 0.1671 | 0.7996 | 0 |
|  | 3 VS 1 | 265 | 0.0009 | -0.0254 | 0.0398 | 0.8916 | 0.1717 | -0.1889 | 0.5009 | 0.3168 | 0.1727 | -0.1811 | 0.4999 | 0.3080 | 0 |
|  | 3 VS 2 | 265 | 0.0010 | -0.0301 | 0.0384 | 0.9480 | 0.1970 | -0.1618 | 0.5524 | 0.2704 | 0.1981 | -0.1586 | 0.5508 | 0.2568 | 0 |
| H11 Depression (total CDSS) | 2 VS 1 | 260 | 0.0196 | -0.0227 | 0.0723 | 0.3552 | -0.0524 | -0.2515 | 0.1413 | 0.6076 | -0.0328 | -0.2404 | 0.1624 | 0.7696 | 0 |
|  | 3 VS 1 | 260 | 0.0725 | -0.0051 | 0.1790 | 0.0676 | 0.0954 | -0.2494 | 0.4162 | 0.5688 | 0.1678 | -0.1829 | 0.4919 | 0.3208 | 0 |
|  | 3 VS 2 | 260 | 0.0529 | -0.0286 | 0.1614 | 0.2032 | 0.1478 | -0.2067 | 0.4867 | 0.3988 | 0.2007 | -0.1773 | 0.5510 | 0.2672 | 0 |
| H12 Sleep disturbances (total PSQI) | 2 VS 1 | 261 | 0.0539 | 0.0112 | 0.1166 | **0.0044** | -0.0554 | -0.2474 | 0.1333 | 0.5896 | -0.0015 | -0.1933 | 0.1890 | 0.9876 | **1** |
|  | 3 VS 1 | 261 | 0.0545 | -0.0034 | 0.1385 | 0.0656 | 0.0759 | -0.2406 | 0.3808 | 0.6120 | 0.1304 | -0.2045 | 0.4540 | 0.4204 | 0 |
|  | 3 VS 2 | 261 | 0.0006 | -0.0692 | 0.0645 | 0.9800 | 0.1314 | -0.1854 | 0.4474 | 0.4184 | 0.1320 | -0.2167 | 0.4642 | 0.4444 | 0 |
| **Supplement 10.** Point estimates and uncertainty estimates for total, direct, and indirect effects operationalizing outcome as sum score of PSYRATS-AH item 9. Abbreviations: H#: Hypothesis no.; Class 1: 'variable severity'; Class 2: 'severe neglect and emotional abuse'; Class 3: 'severe poly-trauma'. Indirect: Indirect effect; Direct: Direct effect; Total: Total effect; Lower/upper: lower and upper uncertainty estimates; bh_significant: Benjamini-Hochberg correction (0=non-significant after correction; 1= significant after correction). Measures: Approve: Approve-Voices; BAVQ-R: Beliefs About Voices Questionnaire - Revised; CTQ: Childhood Trauma Questionnaire; BCSS: Brief Core Schema Scale; CDSS: Calgary Depression Scale for Schizophrenia; ERQ: Emotion Regulation Questionnaire; PSQI; Pittsburgh Sleep Quality Index; VPDS: Voice Power Differential Scale. Significance level: p < .05 (highlighted). All CI set at 95%. | | | | | | | | | | | | | | | |

| **Supplement 11**. Statistical fit of latent class analysis (LCA) |  |  |  |  |  |
| --- | --- | --- | --- | --- | --- |
| # Class solution | N | ll | df | AIC | BIC |
| class1 | 266 | -4066.67 | 10 | 8153.33 | 8189.17 |
| class2 | 266 | -3843.34 | 16 | 7718.67 | 7776.01 |
| class3 | 266 | -3696.21 | 22 | 7436.42 | 7515.25 |
| class4 | 266 | -3651.15 | 28 | 7358.30 | 7458.64 |
| class5 | 266 | -3631.96 | 34 | 7331.93 | 7453.77 |
| class6 | 266 | -3629.40 | 40 | 7338.79 | 7482.13 |
| class7 | 266 | -3548.09 | 46 | 7188.18 | 7353.02 |
| class8 | 266 | -3604.52 | 52 | 7313.04 | 7499.38 |
| class9 | 266 | -3535.78 | 58 | 7187.56 | 7395.41 |
| class10 | 266 | -3589.10 | 64 | 7306.19 | 7535.53 |
| **Supplement 11**. Statistical fit of latent class analysis, AIC and BIC values. Abbreviations: AIC: Akaike Information Criterion; BIC: Bayesian Information Criterion; df: degrees of freedom; LCA: latent class analysis; ll: log-likelihood; N: number of participants included; #class solution: number of classes tests of fit. Explanation of calculation: AIC and BIC are goodness-of-fit estimates calculted on the basis of the log-likelihood. Lower AIC and BIC values indicate better statistical fit. | | | | | |

| **Supplement 12.** Medication-adjusted point estimates and uncertainty estimates for total, direct, and indirect effects. | | | | | | | | | | | | | | |
| --- | --- | --- | --- | --- | --- | --- | --- | --- | --- | --- | --- | --- | --- | --- |
|  | Class | N | Indirect | Lower | Upper | P | Direct | Lower | Upper | P | Total | Lower | Upper | P |
| H1 Negative voice-content (items 6+7 PSYRATS-AHS) | 2 VS 1 | 266 | -0.033 | -0.278 | 0.181 | 0.776 | -0.042 | -0.320 | 0.234 | 0.782 | -0.075 | -0.417 | 0.264 | 0.676 |
|  | 3 VS 1 | 266 | 0.148 | -0.164 | 0.422 | 0.306 | 0.095 | -0.365 | 0.552 | 0.626 | 0.243 | -0.389 | 0.800 | 0.389 |
|  | 3 VS 2 | 266 | 0.181 | -0.127 | 0.481 | 0.242 | 0.138 | -0.329 | 0.614 | 0.557 | 0.318 | -0.316 | 0.881 | 0.298 |
| H2 Persecutory belief factor (subscale BAVQ-R) | 2 VS 1 | 265 | 0.039 | -0.110 | 0.203 | 0.621 | -0.115 | -0.449 | 0.184 | 0.481 | -0.076 | -0.422 | 0.255 | 0.679 |
|  | 3 VS 1 | 265 | 0.259 | 0.008 | 0.525 | **0.042** | -0.085 | -0.630 | 0.425 | 0.797 | 0.174 | -0.456 | 0.739 | 0.551 |
|  | 3 VS 2 | 265 | 0.219 | -0.039 | 0.498 | 0.092 | 0.030 | -0.539 | 0.569 | 0.868 | 0.250 | -0.384 | 0.839 | 0.401 |
| H3 Voice power differential (total VPDS) | 2 VS 1 | 263 | 0.064 | -0.048 | 0.177 | 0.266 | -0.109 | -0.439 | 0.213 | 0.512 | -0.045 | -0.390 | 0.293 | 0.788 |
|  | 3 VS 1 | 263 | 0.170 | 0.005 | 0.360 | **0.044** | 0.074 | -0.486 | 0.585 | 0.734 | 0.245 | -0.351 | 0.816 | 0.386 |
|  | 3 VS 2 | 263 | 0.106 | -0.056 | 0.301 | 0.216 | 0.183 | -0.392 | 0.708 | 0.493 | 0.290 | -0.317 | 0.867 | 0.326 |
| H4 Assertive relating (subscale Approve) | 2 VS 1 | 265 | 0.028 | -0.038 | 0.112 | 0.417 | -0.124 | -0.466 | 0.216 | 0.477 | -0.096 | -0.440 | 0.247 | 0.604 |
|  | 3 VS 1 | 265 | -0.069 | -0.220 | 0.054 | 0.253 | 0.290 | -0.372 | 0.881 | 0.333 | 0.221 | -0.403 | 0.778 | 0.414 |
|  | 3 VS 2 | 265 | -0.097 | -0.260 | 0.029 | 0.126 | 0.414 | -0.228 | 1.016 | 0.201 | 0.317 | -0.290 | 0.872 | 0.282 |
| H5 Aggressive responding (subscale Approve) | 2 VS 1 | 262 | -0.023 | -0.087 | 0.012 | 0.247 | -0.088 | -0.443 | 0.260 | 0.624 | -0.111 | -0.468 | 0.238 | 0.537 |
|  | 3 VS 1 | 262 | -0.096 | -0.283 | 0.030 | 0.147 | 0.407 | -0.263 | 1.011 | 0.218 | 0.311 | -0.306 | 0.848 | 0.287 |
|  | 3 VS 2 | 262 | -0.073 | -0.240 | 0.029 | 0.187 | 0.495 | -0.161 | 1.125 | 0.131 | 0.422 | -0.180 | 1.011 | 0.159 |
| H6 Passive/submissive responding (subsacle Approve) | 2 VS 1 | 264 | 0.040 | -0.066 | 0.144 | 0.451 | -0.139 | -0.486 | 0.183 | 0.398 | -0.099 | -0.463 | 0.226 | 0.553 |
|  | 3 VS 1 | 264 | 0.171 | -0.009 | 0.392 | 0.063 | 0.137 | -0.433 | 0.646 | 0.590 | 0.308 | -0.294 | 0.862 | 0.290 |
|  | 3 VS 2 | 264 | 0.130 | -0.057 | 0.354 | 0.169 | 0.276 | -0.293 | 0.799 | 0.301 | 0.406 | -0.186 | 0.977 | 0.166 |
| H7 Negative other-beliefs (subscale BCSS) | 2 VS 1 | 261 | 0.008 | -0.016 | 0.045 | 0.567 | -0.076 | -0.423 | 0.267 | 0.696 | -0.068 | -0.412 | 0.270 | 0.738 |
|  | 3 VS 1 | 261 | 0.009 | -0.032 | 0.074 | 0.718 | 0.238 | -0.358 | 0.791 | 0.420 | 0.247 | -0.362 | 0.798 | 0.404 |
|  | 3 VS 2 | 261 | 0.001 | -0.047 | 0.057 | 0.967 | 0.314 | -0.302 | 0.884 | 0.308 | 0.315 | -0.313 | 0.896 | 0.310 |
| H8 Negative self-beliefs (subscale BCSS) | 2 VS 1 | 261 | 0.056 | -0.012 | 0.155 | 0.123 | -0.112 | -0.462 | 0.227 | 0.538 | -0.057 | -0.408 | 0.274 | 0.782 |
|  | 3 VS 1 | 261 | 0.092 | -0.008 | 0.242 | 0.077 | 0.170 | -0.437 | 0.713 | 0.543 | 0.262 | -0.353 | 0.821 | 0.369 |
|  | 3 VS 2 | 261 | 0.037 | -0.080 | 0.170 | 0.536 | 0.282 | -0.337 | 0.825 | 0.347 | 0.319 | -0.310 | 0.882 | 0.306 |
| H9 Cognitive reappraisal (subscale ERQ) | 2 VS 1 | 265 | 0.020 | -0.026 | 0.088 | 0.422 | -0.091 | -0.446 | 0.251 | 0.619 | -0.071 | -0.419 | 0.268 | 0.690 |
|  | 3 VS 1 | 265 | 0.001 | -0.084 | 0.099 | 0.952 | 0.246 | -0.357 | 0.794 | 0.389 | 0.247 | -0.376 | 0.805 | 0.395 |
|  | 3 VS 2 | 265 | -0.019 | -0.122 | 0.073 | 0.670 | 0.337 | -0.267 | 0.896 | 0.260 | 0.318 | -0.281 | 0.887 | 0.295 |
| H10 Expressive supression (subscale ERQ) | 2 VS 1 | 265 | -0.004 | -0.048 | 0.044 | 0.873 | -0.067 | -0.407 | 0.262 | 0.701 | -0.071 | -0.419 | 0.268 | 0.690 |
|  | 3 VS 1 | 265 | -0.017 | -0.109 | 0.064 | 0.686 | 0.263 | -0.368 | 0.828 | 0.373 | 0.247 | -0.376 | 0.805 | 0.395 |
|  | 3 VS 2 | 265 | -0.012 | -0.111 | 0.070 | 0.761 | 0.330 | -0.280 | 0.914 | 0.276 | 0.318 | -0.281 | 0.887 | 0.295 |
| H11 Depression (total CDSS) | 2 VS 1 | 260 | 0.039 | -0.059 | 0.141 | 0.422 | -0.122 | -0.475 | 0.207 | 0.510 | -0.084 | -0.448 | 0.262 | 0.680 |
|  | 3 VS 1 | 260 | 0.163 | -0.013 | 0.382 | 0.070 | 0.075 | -0.511 | 0.625 | 0.768 | 0.237 | -0.384 | 0.785 | 0.422 |
|  | 3 VS 2 | 260 | 0.124 | -0.056 | 0.348 | 0.196 | 0.197 | -0.408 | 0.757 | 0.498 | 0.321 | -0.323 | 0.900 | 0.308 |
| H12 Sleep disturbances (total PSQI) | 2 VS 1 | 261 | 0.096 | 0.020 | 0.202 | **0.007** | -0.136 | -0.477 | 0.213 | 0.445 | -0.040 | -0.376 | 0.307 | 0.840 |
|  | 3 VS 1 | 261 | 0.102 | -0.016 | 0.256 | 0.091 | 0.078 | -0.484 | 0.593 | 0.739 | 0.180 | -0.431 | 0.745 | 0.510 |
|  | 3 VS 2 | 261 | 0.006 | -0.126 | 0.135 | 0.925 | 0.213 | -0.344 | 0.750 | 0.424 | 0.220 | -0.389 | 0.797 | 0.443 |
| **Supplement 12.** Medication-adjusted point estimates and uncertainty estimates for total, direct, and indirect effects. Abbreviations: H#: Hypothesis no.; Class 1: 'variable severity'; Class 2: 'severe neglect and emotional abuse'; Class 3: 'severe poly-trauma'. Indirect: Indirect effect; Direct: Direct effect; Total: Total effect; Lower/upper: lower and upper uncertainty estimates Measures: Approve: Approve-Voices; BAVQ-R: Beliefs About Voices Questionnaire - Revised; CTQ: Childhood Trauma Questionnaire; BCSS: Brief Core Schema Scale; CDSS: Calgary Depression Scale for Schizophrenia; ERQ: Emotion Regulation Questionnaire; PSQI; Pittsburgh Sleep Quality Index; VPDS: Voice Power Differential Scale. Significance level: p < .05 (highlighted). All CI set at 95%. | | | | | | | | | | | | | | |

| **Supplement 13.** Medication-adjusted differences in indrect effect according to gender (m vs. f). | | | | | |
| --- | --- | --- | --- | --- | --- |
|  | Classes | Estimate | Lower | Upper | P |
| H1 Negative voice-content (items 6+7 PSYRATS-AHS) | 2 VS 1 | -0.425 | -0.943 | 0.027 | 0.073 |
|  | 3 VS 1 | -0.300 | -0.966 | 0.241 | 0.295 |
|  | 3 VS 2 | 0.125 | -0.521 | 0.765 | 0.700 |
| H2 Persecutory belief factor (subscale BAVQ-R) | 2 VS 1 | 0.179 | -0.135 | 0.528 | 0.290 |
|  | 3 VS 1 | -0.068 | -0.650 | 0.475 | 0.786 |
|  | 3 VS 2 | -0.247 | -0.864 | 0.332 | 0.378 |
| H3 Voice power differential (total VPDS) | 2 VS 1 | -0.135 | -0.363 | 0.064 | 0.195 |
|  | 3 VS 1 | 0.130 | -0.196 | 0.464 | 0.410 |
|  | 3 VS 2 | 0.265 | -0.064 | 0.628 | 0.105 |
| H4 Assertive relating (subscale Approve) | 2 VS 1 | 0.074 | -0.055 | 0.224 | 0.278 |
|  | 3 VS 1 | -0.092 | -0.415 | 0.154 | 0.449 |
|  | 3 VS 2 | -0.166 | -0.503 | 0.075 | 0.192 |
| H5 Aggressive responding (subscale Approve) | 2 VS 1 | 0.048 | -0.057 | 0.258 | 0.466 |
|  | 3 VS 1 | 0.143 | -0.204 | 0.595 | 0.437 |
|  | 3 VS 2 | 0.096 | -0.201 | 0.463 | 0.571 |
| H6 Passive/submissive responding (subsacle Approve) | 2 VS 1 | 0.054 | -0.151 | 0.265 | 0.586 |
|  | 3 VS 1 | 0.079 | -0.367 | 0.490 | 0.648 |
|  | 3 VS 2 | 0.025 | -0.437 | 0.444 | 0.839 |
| H7 Negative other-beliefs (subscale BCSS) | 2 VS 1 | 0.018 | -0.054 | 0.110 | 0.596 |
|  | 3 VS 1 | 0.065 | -0.130 | 0.274 | 0.516 |
|  | 3 VS 2 | 0.047 | -0.144 | 0.245 | 0.608 |
| H8 Negative self-beliefs (subscale BCSS) | 2 VS 1 | 0.018 | -0.153 | 0.197 | 0.756 |
|  | 3 VS 1 | 0.014 | -0.267 | 0.286 | 0.829 |
|  | 3 VS 2 | -0.004 | -0.288 | 0.271 | 0.968 |
| H9 Cognitive reappraisal (subscale ERQ) | 2 VS 1 | 0.019 | -0.085 | 0.146 | 0.674 |
|  | 3 VS 1 | 0.108 | -0.092 | 0.371 | 0.300 |
|  | 3 VS 2 | 0.088 | -0.141 | 0.373 | 0.447 |
| H10 Expressive supression (subscale ERQ) | 2 VS 1 | 0.032 | -0.096 | 0.178 | 0.631 |
|  | 3 VS 1 | -0.038 | -0.259 | 0.166 | 0.670 |
|  | 3 VS 2 | -0.070 | -0.321 | 0.153 | 0.513 |
| H11 Depression (total CDSS) | 2 VS 1 | -0.024 | -0.241 | 0.169 | 0.812 |
|  | 3 VS 1 | 0.158 | -0.204 | 0.552 | 0.371 |
|  | 3 VS 2 | 0.182 | -0.203 | 0.588 | 0.319 |
| H12 Sleep disturbances (total PSQI) | 2 VS 1 | 0.026 | -0.193 | 0.221 | 0.730 |
|  | 3 VS 1 | 0.161 | -0.109 | 0.446 | 0.220 |
|  | 3 VS 2 | 0.135 | -0.134 | 0.442 | 0.316 |
| **Supplement 13**. Medication-adjusted point estimates and uncertainty estimates for differences in indrect effect according to gender (female VS male). Abbreviations: H#: Hypothesis no.; Class 1: 'variable severity'; Class 2: 'severe neglect and emotional abuse'; Class 3: 'severe poly-trauma'. Estimate: Estimate of difference in indrect effects comparing female and male participants; Lower/upper: lower and upper uncertainty estimates Measures: Approve: Approve-Voices; BAVQ-R: Beliefs About Voices Questionnaire - Revised; CTQ: Childhood Trauma Questionnaire; BCSS: Brief Core Schema Scale; CDSS: Calgary Depression Scale for Schizophrenia; ERQ: Emotion Regulation Questionnaire; PSQI; Pittsburgh Sleep Quality Index; VPDS: Voice Power Differential Scale. Significance level: p < .05 (highlighted). All CI set at 95%. | | | | | |

| **Supplement 14.** Voice duration-adjusted point estimates and uncertainty estimates for total, direct, and indirect effects. | | | | | | | | | | | | | | |
| --- | --- | --- | --- | --- | --- | --- | --- | --- | --- | --- | --- | --- | --- | --- |
|  | Class | N | Indirect | Lower | Upper | P | Direct | Lower | Upper | P | Total | Lower | Upper | P |
| H1 Negative voice-content (items 6+7 PSYRATS-AHS) | 2 VS 1 | 266 | -0.034 | -0.272 | 0.189 | 0.778 | -0.020 | -0.288 | 0.242 | 0.881 | -0.054 | -0.404 | 0.277 | 0.762 |
|  | 3 VS 1 | 266 | 0.135 | -0.194 | 0.434 | 0.381 | 0.090 | -0.361 | 0.529 | 0.650 | 0.225 | -0.439 | 0.793 | 0.441 |
|  | 3 VS 2 | 266 | 0.169 | -0.163 | 0.485 | 0.291 | 0.111 | -0.359 | 0.568 | 0.621 | 0.279 | -0.406 | 0.855 | 0.369 |
| H2 Persecutory belief factor (subscale BAVQ-R) | 2 VS 1 | 265 | 0.035 | -0.119 | 0.200 | 0.664 | -0.093 | -0.410 | 0.210 | 0.558 | -0.058 | -0.410 | 0.280 | 0.746 |
|  | 3 VS 1 | 265 | 0.271 | 0.005 | 0.551 | **0.044** | -0.121 | -0.700 | 0.404 | 0.705 | 0.150 | -0.516 | 0.752 | 0.599 |
|  | 3 VS 2 | 265 | 0.236 | -0.029 | 0.519 | 0.081 | -0.028 | -0.620 | 0.502 | 0.945 | 0.207 | -0.458 | 0.808 | 0.482 |
| H3 Voice power differential (total VPDS) | 2 VS 1 | 263 | 0.070 | -0.040 | 0.187 | 0.214 | -0.089 | -0.421 | 0.231 | 0.578 | -0.019 | -0.359 | 0.311 | 0.907 |
|  | 3 VS 1 | 263 | 0.175 | 0.007 | 0.380 | **0.042** | 0.059 | -0.526 | 0.566 | 0.772 | 0.234 | -0.395 | 0.809 | 0.416 |
|  | 3 VS 2 | 263 | 0.106 | -0.065 | 0.302 | 0.224 | 0.148 | -0.450 | 0.670 | 0.569 | 0.253 | -0.387 | 0.829 | 0.397 |
| H4 Assertive relating (subscale Approve) | 2 VS 1 | 265 | 0.024 | -0.039 | 0.105 | 0.458 | -0.123 | -0.448 | 0.205 | 0.462 | -0.099 | -0.432 | 0.228 | 0.572 |
|  | 3 VS 1 | 265 | -0.068 | -0.217 | 0.054 | 0.254 | 0.236 | -0.445 | 0.818 | 0.433 | 0.168 | -0.474 | 0.722 | 0.530 |
|  | 3 VS 2 | 265 | -0.092 | -0.255 | 0.031 | 0.140 | 0.359 | -0.313 | 0.974 | 0.276 | 0.267 | -0.365 | 0.833 | 0.364 |
| H5 Aggressive responding (subscale Approve) | 2 VS 1 | 262 | -0.027 | -0.095 | 0.013 | 0.227 | -0.070 | -0.419 | 0.286 | 0.696 | -0.097 | -0.447 | 0.250 | 0.582 |
|  | 3 VS 1 | 262 | -0.090 | -0.282 | 0.036 | 0.174 | 0.377 | -0.345 | 1.018 | 0.272 | 0.287 | -0.375 | 0.855 | 0.352 |
|  | 3 VS 2 | 262 | -0.064 | -0.230 | 0.031 | 0.228 | 0.447 | -0.234 | 1.082 | 0.200 | 0.384 | -0.255 | 0.973 | 0.227 |
| H6 Passive/submissive responding (subsacle Approve) | 2 VS 1 | 264 | 0.018 | -0.084 | 0.112 | 0.726 | -0.094 | -0.439 | 0.227 | 0.564 | -0.076 | -0.444 | 0.255 | 0.653 |
|  | 3 VS 1 | 264 | 0.160 | -0.017 | 0.377 | 0.079 | 0.127 | -0.497 | 0.654 | 0.636 | 0.287 | -0.368 | 0.845 | 0.345 |
|  | 3 VS 2 | 264 | 0.143 | -0.042 | 0.364 | 0.128 | 0.221 | -0.406 | 0.763 | 0.428 | 0.363 | -0.288 | 0.928 | 0.241 |
| H7 Negative other-beliefs (subscale BCSS) | 2 VS 1 | 261 | 0.010 | -0.016 | 0.051 | 0.527 | -0.065 | -0.401 | 0.278 | 0.753 | -0.055 | -0.395 | 0.283 | 0.801 |
|  | 3 VS 1 | 261 | 0.008 | -0.036 | 0.070 | 0.770 | 0.226 | -0.416 | 0.792 | 0.461 | 0.234 | -0.417 | 0.797 | 0.444 |
|  | 3 VS 2 | 261 | -0.002 | -0.055 | 0.054 | 0.939 | 0.291 | -0.367 | 0.873 | 0.367 | 0.289 | -0.382 | 0.869 | 0.377 |
| H8 Negative self-beliefs (subscale BCSS) | 2 VS 1 | 261 | 0.061 | -0.008 | 0.165 | 0.096 | -0.094 | -0.445 | 0.234 | 0.611 | -0.033 | -0.386 | 0.305 | 0.884 |
|  | 3 VS 1 | 261 | 0.103 | -0.003 | 0.268 | 0.058 | 0.149 | -0.501 | 0.701 | 0.614 | 0.252 | -0.389 | 0.827 | 0.423 |
|  | 3 VS 2 | 261 | 0.041 | -0.078 | 0.186 | 0.498 | 0.243 | -0.413 | 0.796 | 0.431 | 0.285 | -0.384 | 0.867 | 0.388 |
| H9 Cognitive reappraisal (subscale ERQ) | 2 VS 1 | 265 | 0.016 | -0.030 | 0.081 | 0.500 | -0.066 | -0.414 | 0.267 | 0.718 | -0.050 | -0.390 | 0.280 | 0.784 |
|  | 3 VS 1 | 265 | 0.006 | -0.074 | 0.105 | 0.843 | 0.224 | -0.415 | 0.775 | 0.448 | 0.230 | -0.419 | 0.801 | 0.450 |
|  | 3 VS 2 | 265 | -0.010 | -0.105 | 0.083 | 0.840 | 0.290 | -0.361 | 0.847 | 0.340 | 0.280 | -0.373 | 0.861 | 0.356 |
| H10 Expressive supression (subscale ERQ) | 2 VS 1 | 265 | 0.002 | -0.037 | 0.053 | 0.873 | -0.053 | -0.397 | 0.272 | 0.762 | -0.050 | -0.390 | 0.280 | 0.784 |
|  | 3 VS 1 | 265 | -0.014 | -0.101 | 0.061 | 0.728 | 0.244 | -0.407 | 0.825 | 0.429 | 0.230 | -0.419 | 0.801 | 0.450 |
|  | 3 VS 2 | 265 | -0.017 | -0.112 | 0.056 | 0.668 | 0.297 | -0.372 | 0.882 | 0.336 | 0.280 | -0.373 | 0.861 | 0.356 |
| H11 Depression (total CDSS) | 2 VS 1 | 260 | 0.039 | -0.066 | 0.150 | 0.445 | -0.098 | -0.444 | 0.231 | 0.590 | -0.059 | -0.424 | 0.280 | 0.768 |
|  | 3 VS 1 | 260 | 0.173 | -0.012 | 0.399 | 0.066 | 0.051 | -0.562 | 0.598 | 0.844 | 0.224 | -0.421 | 0.804 | 0.462 |
|  | 3 VS 2 | 260 | 0.134 | -0.059 | 0.369 | 0.183 | 0.150 | -0.462 | 0.715 | 0.620 | 0.284 | -0.390 | 0.880 | 0.379 |
| H12 Sleep disturbances (total PSQI) | 2 VS 1 | 261 | 0.105 | 0.023 | 0.221 | **0.004** | -0.123 | -0.464 | 0.224 | 0.489 | -0.018 | -0.354 | 0.333 | 0.937 |
|  | 3 VS 1 | 261 | 0.119 | -0.005 | 0.288 | 0.063 | 0.047 | -0.563 | 0.593 | 0.841 | 0.166 | -0.481 | 0.754 | 0.560 |
|  | 3 VS 2 | 261 | 0.014 | -0.128 | 0.153 | 0.836 | 0.170 | -0.413 | 0.719 | 0.536 | 0.184 | -0.455 | 0.782 | 0.537 |
| **Supplement 14.** Voice duration-adjusted point estimates and uncertainty estimates for total, direct, and indirect effects. Abbreviations: H#: Hypothesis no.; Class 1: 'variable severity'; Class 2: 'severe neglect and emotional abuse'; Class 3: 'severe poly-trauma'. Indirect: Indirect effect; Direct: Direct effect; Total: Total effect; Lower/upper: lower and upper uncertainty estimates Measures: Approve: Approve-Voices; BAVQ-R: Beliefs About Voices Questionnaire - Revised; CTQ: Childhood Trauma Questionnaire; BCSS: Brief Core Schema Scale; CDSS: Calgary Depression Scale for Schizophrenia; ERQ: Emotion Regulation Questionnaire; PSQI; Pittsburgh Sleep Quality Index; VPDS: Voice Power Differential Scale. Significance level: p < .05 (highlighted). All CI set at 95%. | | | | | | | | | | | | | | |

| **Supplement 15.** Voice duration-adjusted differences in indrect effect according to gender (m vs. f). | | | | | |
| --- | --- | --- | --- | --- | --- |
|  | Classes | Estimate | Lower | Upper | P |
| H1 Negative voice-content (items 6+7 PSYRATS-AHS) | 2 VS 1 | -0.437 | -0.962 | 0.009 | 0.053 |
|  | 3 VS 1 | -0.337 | -1.055 | 0.214 | 0.258 |
|  | 3 VS 2 | 0.100 | -0.619 | 0.776 | 0.762 |
| H2 Persecutory belief factor (subscale BAVQ-R) | 2 VS 1 | 0.160 | -0.157 | 0.498 | 0.335 |
|  | 3 VS 1 | -0.086 | -0.668 | 0.470 | 0.738 |
|  | 3 VS 2 | -0.246 | -0.873 | 0.331 | 0.401 |
| H3 Voice power differential (total VPDS) | 2 VS 1 | -0.147 | -0.377 | 0.054 | 0.160 |
|  | 3 VS 1 | 0.113 | -0.216 | 0.452 | 0.466 |
|  | 3 VS 2 | 0.260 | -0.076 | 0.616 | 0.117 |
| H4 Assertive relating (subscale Approve) | 2 VS 1 | 0.066 | -0.063 | 0.212 | 0.320 |
|  | 3 VS 1 | -0.086 | -0.400 | 0.152 | 0.469 |
|  | 3 VS 2 | -0.151 | -0.488 | 0.083 | 0.223 |
| H5 Aggressive responding (subscale Approve) | 2 VS 1 | 0.056 | -0.053 | 0.273 | 0.404 |
|  | 3 VS 1 | 0.171 | -0.188 | 0.637 | 0.348 |
|  | 3 VS 2 | 0.116 | -0.173 | 0.492 | 0.468 |
| H6 Passive/submissive responding (subsacle Approve) | 2 VS 1 | 0.049 | -0.131 | 0.241 | 0.582 |
|  | 3 VS 1 | 0.100 | -0.354 | 0.524 | 0.570 |
|  | 3 VS 2 | 0.051 | -0.429 | 0.458 | 0.740 |
| H7 Negative other-beliefs (subscale BCSS) | 2 VS 1 | 0.022 | -0.053 | 0.118 | 0.536 |
|  | 3 VS 1 | 0.062 | -0.150 | 0.275 | 0.565 |
|  | 3 VS 2 | 0.039 | -0.166 | 0.238 | 0.696 |
| H8 Negative self-beliefs (subscale BCSS) | 2 VS 1 | 0.009 | -0.179 | 0.187 | 0.852 |
|  | 3 VS 1 | -0.008 | -0.323 | 0.262 | 0.980 |
|  | 3 VS 2 | -0.017 | -0.315 | 0.244 | 0.937 |
| H9 Cognitive reappraisal (subscale ERQ) | 2 VS 1 | 0.013 | -0.099 | 0.133 | 0.776 |
|  | 3 VS 1 | 0.097 | -0.092 | 0.358 | 0.321 |
|  | 3 VS 2 | 0.084 | -0.135 | 0.368 | 0.444 |
| H10 Expressive supression (subscale ERQ) | 2 VS 1 | 0.044 | -0.084 | 0.192 | 0.500 |
|  | 3 VS 1 | -0.041 | -0.268 | 0.163 | 0.655 |
|  | 3 VS 2 | -0.085 | -0.344 | 0.148 | 0.423 |
| H11 Depression (total CDSS) | 2 VS 1 | -0.042 | -0.279 | 0.161 | 0.686 |
|  | 3 VS 1 | 0.132 | -0.260 | 0.529 | 0.477 |
|  | 3 VS 2 | 0.175 | -0.214 | 0.576 | 0.346 |
| H12 Sleep disturbances (total PSQI) | 2 VS 1 | 0.019 | -0.219 | 0.221 | 0.795 |
|  | 3 VS 1 | 0.121 | -0.183 | 0.412 | 0.384 |
|  | 3 VS 2 | 0.102 | -0.190 | 0.420 | 0.470 |
| **Supplement 15**. Voice duration-adjusted point estimates and uncertainty estimates for differences in indrect effect according to gender (female VS male). Abbreviations: H#: Hypothesis no.; Class 1: 'variable severity'; Class 2: 'severe neglect and emotional abuse'; Class 3: 'severe poly-trauma'. Estimate: Estimate of difference in indrect effects comparing female and male participants; Lower/upper: lower and upper uncertainty estimates Measures: Approve: Approve-Voices; BAVQ-R: Beliefs About Voices Questionnaire - Revised; CTQ: Childhood Trauma Questionnaire; BCSS: Brief Core Schema Scale; CDSS: Calgary Depression Scale for Schizophrenia; ERQ: Emotion Regulation Questionnaire; PSQI; Pittsburgh Sleep Quality Index; VPDS: Voice Power Differential Scale. Significance level: p < .05 (highlighted). All CI set at 95%. | | | | | |

# References

Andrew, E. M., Gray, N. S., & Snowden, R. J. (2008). The relationship between trauma and beliefs about hearing voices: A study of psychiatric and non-psychiatric voice hearers. *Psychological Medicine*, *38*(10), 1409–1417. https://doi.org/10.1017/S003329170700253X

Badcock, J. C., Paulik, G., & Maybery, M. T. (2011). The role of emotion regulation in auditory hallucinations. *Psychiatry Research*, *185*(3), 303–308. https://doi.org/10.1016/j.psychres.2010.07.011

Bagautdinova, J., Mayeli, A., Wilson, J. D., Donati, F. L., Colacot, R. M., Meyer, N., Fusar-Poli, P., & Ferrarelli, F. (2023). Sleep Abnormalities in Different Clinical Stages of Psychosis: A Systematic Review and Meta-analysis. *JAMA Psychiatry*, *80*(3), 202–210. https://doi.org/10.1001/jamapsychiatry.2022.4599

Barnes, G. L., Emsley, R., Garety, P., Hardy, A., & Barnes, G. (2023). Investigating specific associations between childhood victimisation profiles and positive psychosis symptoms: the mediating roles of anxiety, depression and schema. *Schizophrenia Bulletin Open, Sgad017.* https://doi.org/10.1093/schizbullopen/sgad017/7198364

Beavan, V., & Read, J. (2010). Hearing voices and listening to what they say: The importance of voice content in understanding and working with distressing voices. *Journal of Nervous and Mental Disease*, *198*(3). https://doi.org/10.1097/NMD.0b013e3181d14612

Beck, A. T. (2005). The current state of cognitive therapy: a 40-year retrospective. *Archives of General Psychiatry*, *62*(9), 953–959.

Beck, A. T., & Rector, N. A. (2005). Cognitive approaches to schizophrenia: theory and therapy. *Annu. Rev. Clin. Psychol.*, *1*(1), 577–606.

Begemann, M. J. H., Sommer, I. E., Brand, R. M., Oomen, P. P., Jongeneel, A., Berkhout, J., Molenaar, R. E., Wielage, N. N., Toh, W. L., Rossell, S. L., & Bell, I. H. (2022). Auditory verbal hallucinations and childhood trauma subtypes across the psychosis continuum: a cluster analysis. *Cognitive Neuropsychiatry*, *27*(2–3), 150–168. https://doi.org/10.1080/13546805.2021.1925235

Berry, K., Barrowclough, C., & Wearden, A. (2008). Attachment theory: a framework for understanding symptoms and interpersonal relationships in psychosis. *Behaviour Research and Therapy*, *46*(12), 1275–1282.

Berry, K., Wearden, A., Barrowclough, C., Oakland, L., & Bradley, J. (2012). An investigation of adult attachment and the nature of relationships with voices. *British Journal of Clinical Psychology*, *51*(3), 280–291.

Birchwood, M., Gilbert, P., Gilbert, J., Trower, P., Meaden, A., Hay, J., Murray, E., & Miles, J. N. V. (2004). Interpersonal and role-related schema influence the relationship with the dominant ‘voice’in schizophrenia: a comparison of three models. *Psychological Medicine*, *34*(8), 1571–1580.

Birchwood, M., Meaden, A., Trower, P., Gilbert, P., & Plaistow, J. (2000). The power and omnipotence of voices: Subordination and entrapment by voices and significant others. *Psychological Medicine*, *30*(2), 337–344. https://doi.org/10.1017/S0033291799001828

Carter, B., Wootten, J., Archie, S., Terry, A. L., & Anderson, K. K. (2022). Sex and gender differences in symptoms of early psychosis: a systematic review and meta-analysis. In *Archives of Women’s Mental Health* (Vol. 25, Issue 4, pp. 679–691). Springer. https://doi.org/10.1007/s00737-022-01247-3

Chadwick, P., & Birchwood, M. (1994). The omnipotence of voices: A cognitive approach to auditory hallucinations. *The British Journal of Psychiatry*, *164*(2), 190–201.

Chapman, H. C., Visser, K. F., Mittal, V. A., Gibb, B. E., Coles, M. E., & Strauss, G. P. (2019). Emotion regulation across the psychosis continuum. *Development and Psychopathology*, *32*(1), 219–227. https://doi.org/10.1017/S0954579418001682

Cole, E. R., Strauss, C., Fife‐Schaw, C., & McCarthy‐Jones, S. (2017). Echoes of others: A path analytic examination of an interpersonal–cognitive model of voice‐related distress. *Psychology and Psychotherapy: Theory, Research and Practice*, *90*(4), 617–632.

Connor, C., & Birchwood, M. (2013). Power and perceived expressed emotion of voices: Their impact on depression and suicidal thinking in those who hear voices. *Clinical Psychology and Psychotherapy*, *20*(3), 199–205. https://doi.org/10.1002/cpp.798

Copolov, D. L., Mackinnon, A., & Trauer, T. (2004). Correlates of the affective impact of auditory hallucinations in psychotic disorders. *Schizophrenia Bulletin*, *30*(1), 163–171.

Corstens, D., & Longden, E. (2013). The origins of voices: Links between life history and voice hearing in a survey of 100 cases. *Psychosis*, *5*(3), 270–285. https://doi.org/10.1080/17522439.2013.816337

Corstens, D., Longden, E., McCarthy-Jones, S., Waddingham, R., & Thomas, N. (2014). Emerging perspectives from the hearing voices movement: Implications for research and practice. In *Schizophrenia Bulletin* (Vol. 40, Issue SUPPL. 4). Oxford University Press. https://doi.org/10.1093/schbul/sbu007

Cutuli, D. (2014). Cognitive reappraisal and expressive suppression strategies role in the emotion regulation: An overview on their modulatory effects and neural correlates. In *Frontiers in Systems Neuroscience* (Vol. 8). Frontiers Research Foundation. https://doi.org/10.3389/fnsys.2014.00175

Daalman, K., Boks, M. P. M., Diederen, K. M. J., de Weijer, A. D., Blom, J. D., Kahn, R. S., & Sommer, I. E. C. (2011). The same or different? A phenomenological comparison of auditory verbal hallucinations in healthy and psychotic individuals. *The Journal of Clinical Psychiatry*, *72*(3), 18878.

Desantis, S. M., Baker, N. L., Back, S. E., Spratt, E., Ciolino, J. D., Moran-Santa Maria, M., Dipankar, B., & Brady, K. T. (2011). Gender differences in the effect of early life trauma on hypothalamic-pituitary-adrenal axis functioning. *Depression and Anxiety*, *28*(5), 383–392. https://doi.org/10.1002/da.20795

Dragioti, E., Radua, J., Solmi, M., Arango, C., Oliver, D., Cortese, S., Jones, P. B., Il Shin, J., Correll, C. U., & Fusar-Poli, P. (2022). Global population attributable fraction of potentially modifiable risk factors for mental disorders: a meta-umbrella systematic review. *Molecular Psychiatry*, *27*(8), 3510–3519. https://doi.org/10.1038/s41380-022-01586-8

Freeman, D., & Garety, P. (2014). Advances in understanding and treating persecutory delusions: A review. In *Social Psychiatry and Psychiatric Epidemiology* (Vol. 49, Issue 8, pp. 1179–1189). Dr. Dietrich Steinkopff Verlag GmbH and Co. KG. https://doi.org/10.1007/s00127-014-0928-7

Frost, R., Collier, O., & Hardy, A. (2024). Are trauma-related beliefs associated with psychosis symptoms? A systematic review and meta-analysis. In *Psychological Medicine*. Cambridge University Press. https://doi.org/10.1017/S0033291724002629

Garcia, M., Montalvo, I., Creus, M., Cabezas, Á., Solé, M., Algora, M. J., Moreno, I., Gutiérrez-Zotes, A., & Labad, J. (2016). Sex differences in the effect of childhood trauma on the clinical expression of early psychosis. *Comprehensive Psychiatry*, *68*, 86–96. https://doi.org/10.1016/j.comppsych.2016.04.004

Gibson, L. E., Anglin, D. M., Klugman, J. T., Reeves, L. E., Fineberg, A. M., Maxwell, S. D., Kerns, C. M., & Ellman, L. M. (2014). Stress sensitivity mediates the relationship between traumatic life events and attenuated positive psychotic symptoms differentially bygender in a college population sample. *Journal of Psychiatric Research*, *53*(1), 111–118. https://doi.org/10.1016/j.jpsychires.2014.02.020

Giordano, G. M., Bucci, P., Mucci, A., Pezzella, P., & Galderisi, S. (2021). Gender Differences in Clinical and Psychosocial Features Among Persons With Schizophrenia: A Mini Review. In *Frontiers in Psychiatry* (Vol. 12). Frontiers Media S.A. https://doi.org/10.3389/fpsyt.2021.789179

Gruhn, M. A., & Compas, B. E. (2020). Effects of maltreatment on coping and emotion regulation in childhood and adolescence: A meta-analytic review. In *Child Abuse and Neglect* (Vol. 103). Elsevier Ltd. https://doi.org/10.1016/j.chiabu.2020.104446

Hardy, A., Emsley, R., Freeman, D., Bebbington, P., Garety, P. A., Kuipers, E. E., Dunn, G., & Fowler, D. (2016). Psychological Mechanisms Mediating Effects between Trauma and Psychotic Symptoms: The Role of Affect Regulation, Intrusive Trauma Memory, Beliefs, and Depression. *Schizophrenia Bulletin*, *42*, S34–S43. https://doi.org/10.1093/schbul/sbv175

Hardy, A., Fowler, D., Freeman, D., Smith, B., Steel, C., Evans, J., Garety, P., Kuipers, E., Bebbington, P., & Dunn, G. (2005). Trauma and hallucinatory experience in psychosis. *Journal of Nervous and Mental Disease*, *193*(8), 501–507. https://doi.org/10.1097/01.nmd.0000172480.56308.21

Hartley, S., Barrowclough, C., & Haddock, G. (2013). Anxiety and depression in psychosis: A systematic review of associations with positive psychotic symptoms. In *Acta Psychiatrica Scandinavica* (Vol. 128, Issue 5, pp. 327–346). https://doi.org/10.1111/acps.12080

Hayward, M. (2003). Interpersonal relating and voice hearing: To what extent does relating to the voice reflect social relating? *Psychology and Psychotherapy: Theory, Research and Practice*, *76*(4), 369–383. https://doi.org/10.1348/147608303770584737

Hayward, M., Berry, K., & Ashton, A. (2011). Applying interpersonal theories to the understanding of and therapy for auditory hallucinations: A review of the literature and directions for further research. In *Clinical Psychology Review* (Vol. 31, Issue 8, pp. 1313–1323). https://doi.org/10.1016/j.cpr.2011.09.001

Hayward, M., Denney, J., Vaughan, S., & Fowler, D. (2008). The voice and you: Development and psychometric evaluation of a measure of relationships with voices. *Clinical Psychology and Psychotherapy*, *15*(1), 45–52. https://doi.org/10.1002/cpp.561

Hayward, M., Schlier, B., Strauss, C., Rammou, A., & Lincoln, T. (2020). Construction and validation of the Approve questionnaires – Measures of relating to voices and other people. *Schizophrenia Research*, *220*, 254–260. https://doi.org/10.1016/j.schres.2020.03.003

Hayward, M., Slater, L., Berry, K., & Perona-Garcelán, S. (2016). Establishing the “fit” between the patient and the therapy: The role of patient gender in selecting psychological therapy for distressing voices. *Frontiers in Psychology*, *7*(MAR). https://doi.org/10.3389/fpsyg.2016.00424

Herms, E. N., Bolbecker, A. R., & Wisner, K. M. (2024). Impaired Sleep Mediates the Relationship Between Interpersonal Trauma and Subtypes of Delusional Ideation. *Schizophrenia Bulletin*, *50*(3), 642–652. https://doi.org/10.1093/schbul/sbad081

Jorovat, A., Twumasi, R., Mechelli, A., & Georgiades, A. (2025). Core beliefs in psychosis: a systematic review and meta-analysis. In *Schizophrenia* (Vol. 11, Issue 1). Nature Research. https://doi.org/10.1038/s41537-025-00577-2

Kelly, D. L., Rowland, L. M., Patchan, K. M., Sullivan, K., Earl, A., Raley, H., Liu, F., Feldman, S., & McMahon, R. P. (2016). Schizophrenia clinical symptom differences in women vs. men with and without a history of childhood physical abuse. *Child and Adolescent Psychiatry and Mental Health*, *10*(1). https://doi.org/10.1186/s13034-016-0092-9

Köhler-Forsberg, O., Ge, F., Hauksdóttir, A., Thordardottir, E. B., Ásbjörnsdóttir, K., Rúnarsdóttir, H., Tómasson, G., Jakobsdóttir, J., Guðmundsdóttir, B., Björnsson, A. S., Sigurðsson, E., Aspelund, T., & Valdimarsdottir, U. A. (2024). Adverse childhood experiences and psychological functioning among women with schizophrenia or bipolar disorder: population-based study. *British Journal of Psychiatry*, *224*(1), 6–12. https://doi.org/10.1192/bjp.2023.128

Kusztrits, I., Toh, W. L., Thomas, N., Larøi, F., Meyers, D., Hirnstein, M., & Rossell, S. (2022). From core schemas about the self and others to voice phenomenology: Anxiety and depression affect voice hearers differently. *Psychology and Psychotherapy: Theory, Research and Practice*, *95*(2), 493–507. https://doi.org/10.1111/papt.12384

Laloyaux, J., Dessart, G., Van Der Linden, M., Lemaire, M., & Larøi, F. (2016). Maladaptive emotion regulation strategies and stress sensitivity mediate the relation between adverse life events and attenuated positive psychotic symptoms. *Cognitive Neuropsychiatry*, *21*(2), 116–129. https://doi.org/10.1080/13546805.2015.1137213

Larøi, F., Thomas, N., Aleman, A., Fernyhough, C., Wilkinson, S., Deamer, F., & McCarthy-Jones, S. (2019). The ice in voices: Understanding negative content in auditory-verbal hallucinations. In *Clinical Psychology Review* (Vol. 67, pp. 1–10). Elsevier Inc. https://doi.org/10.1016/j.cpr.2018.11.001

Laskemoen, J. F., Aas, M., Vaskinn, A., Berg, A. O., Lunding, S. H., Barrett, E. A., Melle, I., & Simonsen, C. (2021). Sleep disturbance mediates the link between childhood trauma and clinical outcome in severe mental disorders. *Psychological Medicine*, *51*(14), 2337–2346. https://doi.org/10.1017/S0033291720000914

Lavi, I., Katz, L. F., Ozer, E. J., & Gross, J. J. (2019). Emotion Reactivity and Regulation in Maltreated Children: A Meta-Analysis. *Child Development*, *90*(5), 1503–1524. https://doi.org/10.1111/cdev.13272

León-Palacios, M. de G., Úbeda-Gómez, J., Escudero-Pérez, S., Barros-Albarán, M. D., López-Jiménez, A. M. aría, & Perona-Garcelán, S. (2015). Auditory Verbal Hallucinations: can Beliefs about Voices Mediate the Relationship Patients establish with them and Negative Affect? *The Spanish Journal of Psychology*, *18*, E76. https://doi.org/10.1017/sjp.2015.77

Lincoln, T. M., Marin, N., & Jaya, E. S. (2017). Childhood trauma and psychotic experiences in a general population sample: A prospective study on the mediating role of emotion regulation. *European Psychiatry*, *42*, 111–119. https://doi.org/10.1016/j.eurpsy.2016.12.010

Liu, J., Teh, W. L., Tan, R. H. S., Tan, Y. B., Tang, C., Chandwani, N., & Subramaniam, M. (2023). Sleep disturbance as transdiagnostic mediator between adverse childhood experiences and psychopathology in children and adolescents: A structural equation modeling meta-analysis. In *JCPP Advances* (Vol. 3, Issue 3). John Wiley and Sons Inc. https://doi.org/10.1002/jcv2.12156

Longden, E., Corstens, D., Escher, S., & Romme, M. (2012). Voice hearing in a biographical context: A model for formulating the relationship between voices and life history. *Psychosis*, *4*(3), 224–234. https://doi.org/10.1080/17522439.2011.596566

Ludwig, L., Werner, D., & Lincoln, T. M. (2019). The relevance of cognitive emotion regulation to psychotic symptoms – A systematic review and meta-analysis. In *Clinical Psychology Review* (Vol. 72). Elsevier Inc. https://doi.org/10.1016/j.cpr.2019.101746

Mawson, A., Berry, K., Murray, C., & Hayward, M. (2011). Voice hearing within the context of hearers’ social worlds: An interpretative phenomenological analysis. *Psychology and Psychotherapy: Theory, Research and Practice*, *84*(3), 256–272. https://doi.org/10.1348/147608310X524883

Mawson, A., Cohen, K., & Berry, K. (2010). Reviewing evidence for the cognitive model of auditory hallucinations: The relationship between cognitive voice appraisals and distress during psychosis. In *Clinical Psychology Review* (Vol. 30, Issue 2, pp. 248–258). https://doi.org/10.1016/j.cpr.2009.11.006

McCarthy-Jones, S., Thomas, N., Strauss, C., Dodgson, G., Jones, N., Woods, A., Brewin, C. R., Hayward, M., Stephane, M., Barton, J., Kingdon, D., & Sommer, I. E. (2014). Better than mermaids and stray dogs? subtyping auditory verbal hallucinations and its implications for research and practice. *Schizophrenia Bulletin*, *40*(SUPPL. 4). https://doi.org/10.1093/schbul/sbu018

Miu, A. C., Szentágotai-Tătar, A., Balázsi, R., Nechita, D., Bunea, I., & Pollak, S. D. (2022). Emotion regulation as mediator between childhood adversity and psychopathology: A meta-analysis. *Clinical Psychology Review*, *93*, 102141. https://doi.org/10.1016/J.CPR.2022.102141

Moran, E. K., Culbreth, A. J., & Barch, D. M. (2018). Emotion Regulation Predicts Everyday Emotion Experience and Social Function in Schizophrenia. *Clinical Psychological Science*, *6*(2), 271–279. https://doi.org/10.1177/2167702617738827

Morrison, A. P., & Wells, A. (2007). Relationships between worry, psychotic experiences and emotional distress in patients with schizophrenia spectrum diagnoses and comparisons with anxious and non-patient groups. *Behaviour Research and Therapy*, *45*(7), 1593–1600. https://doi.org/10.1016/j.brat.2006.11.010

Murphy, J., Shevlin, M., Adamson, G., & Houston, J. E. (2010). Positive psychosis symptom structure in the general population: Assessing dimensional consistency and continuity from “pathology” to “normality.” *Psychosis*, *2*(3), 199–209. https://doi.org/10.1080/17522430903437087

Nayani, T. H., & David, A. S. (1996). The auditory hallucination: a phenomenological survey. In *Psychological Medicine* (Vol. 26).

O’Brien, C., Rus-Calafell, M., Craig, T. K. J., Garety, P., Ward, T., Lister, R., & Fornells-Ambrojo, M. (2021). Relating behaviours and therapeutic actions during AVATAR therapy dialogue: An observational study. *British Journal of Clinical Psychology*, *60*(4), 443–462. https://doi.org/10.1111/bjc.12296

Ochoa, S., Usall, J., Cobo, J., Labad, X., & Kulkarni, J. (2012). Gender Differences in Schizophrenia and First-Episode Psychosis: A Comprehensive Literature Review. *Schizophrenia Research and Treatment*, *2012*, 1–9. https://doi.org/10.1155/2012/916198

O’Driscoll, C., Laing, J., & Mason, O. (2014). Cognitive emotion regulation strategies, alexithymia and dissociation in schizophrenia, a review and meta-analysis. In *Clinical Psychology Review* (Vol. 34, Issue 6, pp. 482–495). Elsevier Inc. https://doi.org/10.1016/j.cpr.2014.07.002

Paulik, G. (2012). The Role of Social Schema in the Experience of Auditory Hallucinations: A Systematic Review and a Proposal for the Inclusion of Social Schema in a Cognitive Behavioural Model of Voice Hearing. *Clinical Psychology and Psychotherapy*, *19*(6), 459–472. https://doi.org/10.1002/cpp.768

Peters, E. R., Williams, S. L., Cooke, M. A., & Kuipers, E. (2012). It’s not what you hear, it’s the way you think about it: Appraisals as determinants of affect and behaviour in voice hearers. *Psychological Medicine*, *42*(7), 1507–1514. https://doi.org/10.1017/S0033291711002650

Rammou, A., Berry, C., Fowler, D., & Hayward, M. (2022). Distress factors of voice-hearing in young people and social relating: Exploring a cognitive-interpersonal voice-hearing model. *Psychology and Psychotherapy: Theory, Research and Practice*, *95*(4), 939–957. https://doi.org/10.1111/papt.12411

Reeve, S., Sheaves, B., & Freeman, D. (2015). The role of sleep dysfunction in the occurrence of delusions and hallucinations: A systematic review. In *Clinical Psychology Review* (Vol. 42, pp. 96–115). Elsevier Inc. https://doi.org/10.1016/j.cpr.2015.09.001

Reiff, M., Castille, D. M., Muenzenmaier, K., & Link, B. (2012). Childhood abuse and the content of adult psychotic symptoms. *Psychological Trauma: Theory, Research, Practice, and Policy*, *4*(4), 356–369. https://doi.org/10.1037/a0024203

Rosen, C., McCarthy-Jones, S., Jones, N., Chase, K. A., & Sharma, R. P. (2018). Negative voice-content as a full mediator of a relation between childhood adversity and distress ensuing from hearing voices. *Schizophrenia Research*, *199*, 361–366. https://doi.org/10.1016/j.schres.2018.03.030

Schindel-Allon, I., Aderka, I. M., Shahar, G., Stein, M., & Gilboa-Schechtman, E. (2010). Longitudinal associations between post-traumatic distress and depressive symptoms following a traumatic event: A test of three models. *Psychological Medicine*, *40*(10), 1669–1678. https://doi.org/10.1017/S0033291709992248

Schlier, B., Sitara, X., Strauss, C., Rammou, A., Lincoln, T. M., & Hayward, M. (2021). Can Gender Differences in Distress Due to Difficult Voices Be Explained by Differences in Relating? *Cognitive Therapy and Research*, *45*(4), 831–839. https://doi.org/10.1007/s10608-020-10190-5

Schønning, V., Sivertsen, B., Hysing, M., Dovran, A., & Askeland, K. G. (2022). Childhood maltreatment and sleep in children and adolescents: A systematic review and meta-analysis. In *Sleep Medicine Reviews* (Vol. 63). W.B. Saunders Ltd. https://doi.org/10.1016/j.smrv.2022.101617

Scott, A. J., Webb, T. L., Martyn-St James, M., Rowse, G., & Weich, S. (2021). Improving sleep quality leads to better mental health: A meta-analysis of randomised controlled trials. In *Sleep Medicine Reviews* (Vol. 60). W.B. Saunders Ltd. https://doi.org/10.1016/j.smrv.2021.101556

Scott, M., Rossell, S. L., Meyer, D., Toh, W. L., & Thomas, N. (2020). Childhood trauma, attachment and negative schemas in relation to negative auditory verbal hallucination (AVH) content. *Psychiatry Research*, *290*. https://doi.org/10.1016/j.psychres.2020.112997

Silver, J. H., Lewton, M., & Lewis, H. W. (2023). Mediators of negative content and voice-related distress in a diverse sample of clinical and non-clinical voice-hearers. *British Journal of Clinical Psychology*, *62*(1), 96–111. https://doi.org/10.1111/bjc.12396

Smith, B., Fowler, D. G., Freeman, D., Bebbington, P., Bashforth, H., Garety, P., Dunn, G., & Kuipers, E. (2006). Emotion and psychosis: Links between depression, self-esteem, negative schematic beliefs and delusions and hallucinations [Article]. *Schizophrenia Research*, *86*(1), 181–188. https://doi.org/10.1016/j.schres.2006.06.018

Sorrell, E., Hayward, M., & Meddings, S. (2010). Interpersonal processes and hearing voices: A study of the association between relating to voices and distress in clinical and non-clinical hearers. *Behavioural and Cognitive Psychotherapy*, *38*(2), 127–140. https://doi.org/10.1017/S1352465809990506

Sun, J. B., Deng, H., Wang, S. Y., Cui, Y. P., Yang, X. J., Wang, C. Y., Chen, Y. H., Yang, Q., Wang, H. N., & Qin, W. (2023). The Feature of Sleep Spindle Deficits in Patients With Schizophrenia With and Without Auditory Verbal Hallucinations. *Biological Psychiatry: Cognitive Neuroscience and Neuroimaging*, *8*(3), 331–342. https://doi.org/10.1016/j.bpsc.2021.07.013

Thomas, N., Farhall, J., & Shawyer, F. (2015). Beliefs about voices and schemas about self and others in psychosis. *Behavioural and Cognitive Psychotherapy*, *43*(2), 209–223.

Thomas, N., McLeod, H. J., & Brewin, C. R. (2009). Interpersonal complementarity in responses to auditory hallucinations in psychosis. *British Journal of Clinical Psychology*, *48*(4), 411–424. https://doi.org/10.1348/014466509X411937

Toh, W. L., Gurvich, C., Thomas, N., Tan, E. J., Neill, E., Van Rheenen, T., Sumner, P. J., Carruthers, S. P., Thomas, E. H., Hughes, M. E., Michie, P. T., & Rossell, S. L. (2020). The influence of gender on emotional aspects of auditory verbal hallucinations. In *Psychiatry Research* (Vol. 284). Elsevier Ireland Ltd. https://doi.org/10.1016/j.psychres.2019.112642

Tsang, A., Bucci, S., Branitsky, A., Kaptan, S., Rafiq, S., Wong, S., Berry, K., & Varese, F. (2021). The relationship between appraisals of voices (auditory verbal hallucinations) and distress in voice-hearers with schizophrenia-spectrum diagnoses: A meta-analytic review. In *Schizophrenia Research* (Vol. 230, pp. 38–47). Elsevier B.V. https://doi.org/10.1016/j.schres.2021.02.013

Vadukapuram, R., Shah, K., Ashraf, S., Srinivas, S., Elshokiry, A. B., Trivedi, C., Mansuri, Z., & Jain, S. (2022). Adverse Childhood Experiences and Their Impact on Sleep in Adults: A Systematic Review. In *Journal of Nervous and Mental Disease* (Vol. 210, Issue 6, pp. 397–410). Lippincott Williams and Wilkins. https://doi.org/10.1097/NMD.0000000000001480

Vaughan, S., & Fowler, D. (2004). The distress experienced by voice hearers is associated with perceived relationship between the voice hearer and the voice. *British Journal of Clinical Psychology*, *43*(2), 143–153. https://doi.org/10.1348/014466504323088024

Ward, T., Lister, R., Fornells-Ambrojo, M., Rus-Calafell, M., Edwards, C. J., O’Brien, C., Craig, T. K. J., & Garety, P. (2022). The role of characterisation in everyday voice engagement and AVATAR therapy dialogue. *Psychological Medicine*, *52*(16), 3846–3853. https://doi.org/10.1017/S0033291721000659

Ward, T., Rus-Calafell, M., Ramadhan, Z., Soumelidou, O., Fornells-Ambrojo, M., Garety, P., & Craig, T. K. J. (2020). AVATAR therapy for distressing voices: A comprehensive account of therapeutic targets. *Schizophrenia Bulletin*, *46*(5), 1038–1044. https://doi.org/10.1093/schbul/sbaa061

Williams, J., Bucci, S., Berry, K., & Varese, F. (2018). Psychological mediators of the association between childhood adversities and psychosis: A systematic review. In *Clinical Psychology Review* (Vol. 65, pp. 175–196). Elsevier Inc. https://doi.org/10.1016/j.cpr.2018.05.009
